# Transcriptional changes of the extracellular matrix in chronic thromboembolic pulmonary hypertension govern right ventricle remodeling and recovery

**DOI:** 10.1038/s44161-025-00672-8

**Published:** 2025-07-04

**Authors:** Leili Jafari, Christoph B. Wiedenroth, Steffen D. Kriechbaum, Dimitri Grün, Prakash Chelladurai, Stefan Guenther, Carsten Kuenne, Alicia M. Späth, Anoop V. Cherian, Christian Troidl, Jochen Wilhelm, Stanislav Keranov, Till Keller, Baktybek Kojonazarov, Ralph T. Schermuly, Stefan Guth, Oliver Dörr, Holger Nef, Mario Boehm, Edda Spiekerkoetter, Przemyslaw Leszek, Zoltan V. Varga, Peter Ferdinandy, Hossein A. Ghofrani, Peter Dorfmüller, Norbert Weißmann, Christian W. Hamm, Eckhard Mayer, Werner Seeger, Christoph Liebetrau, Soni Savai Pullamsetti

**Affiliations:** 1 Max-Planck Institute for Heart and Lung Research, Bad Nauheim, Germany; 2 Department of Internal Medicine I, Justus Liebig University, Giessen, Germany; 3 Department of Thoracic Surgery, Kerckhoff Heart and Lung Center, Bad Nauheim, Germany; 4 Department of Cardiology, Kerckhoff Heart and Lung Center, Bad Nauheim, Germany; 5 Partner Site Rhein-Main, DZHK (German Center for Cardiovascular Research), Frankfurt am Main, Germany; 6 Department of Internal Medicine II/V, Universities of Giessen and Marburg Lung Center (UGMLC), Member of the German Center for Lung Research (DZL), Excellence Cluster Cardio-Pulmonary Institute (CPI), Justus-Liebig University, Giessen, Germany; 7 Institute for Lung Health, Justus Liebig University, Giessen, Germany; 8 Vera Moulton Wall Center for Pulmonary Vascular Disease, Stanford School of Medicine, Stanford University, Stanford, CA USA; 9 Cardiovascular Institute, Stanford University, Stanford, CA USA; 10 Heart Failure and Transplantology Department, Mechanical Circulatory Support and Transplant Department, National Institute of Cardiology, Warsaw, Poland; 11 Department of Pharmacology and Pharmacotherapy, Semmelweis University, Budapest, Hungary; 12 HCEMM-SU Cardiometabolic Immunology Research Group, Department of Pharmacology and Pharmacotherapy, Semmelweis University, Budapest, Hungary; 13 Momentum-SU Cardio-oncology and Cardioimmunology Research Group, Budapest, Hungary; 14 Center for Pharmacology and Drug Research & Development, Semmelweis University, Budapest, Hungary; 15 HUN-REN-SU System Pharmacology Research Group, Department of Pharmacology and Pharmacotherapy, Semmelweis University, Budapest, Hungary; 16 Pharmahungary Group, Szeged, Hungary

**Keywords:** Transcriptomics, Heart failure, Genetic databases, Data processing

## Abstract

Chronic thromboembolic pulmonary hypertension (CTEPH) leads to progressive right ventricular (RV) dysfunction. Pulmonary endarterectomy (PEA) is an established treatment for these patients; however, the molecular mechanisms underlying RV remodeling and recovery remain poorly understood. Here we show that RNA sequencing and histological analysis of RV free wall and septal biopsies from patients with CTEPH reveal extracellular matrix enrichment and cytoskeletal remodeling before PEA. These changes were consistent across an exploratory and confirmatory cohort. Post-PEA samples showed reversal of both histological and transcriptional abnormalities. Key signaling molecules—ANKRD1, IL7R and SERPINE1—were implicated in fibrotic and proliferative pathways, as confirmed in human tissues and experimental models. Our findings identify a reversible gene expression and structural remodeling signature in the RV, linking hemodynamic unloading with molecular recovery. These insights suggest potential therapeutic targets to modulate maladaptive RV remodeling in CTEPH and improve outcomes beyond surgical intervention.

## Abstract (web summary)

Through RNA profiling of right ventricular tissue from patients with chronic thromboembolic pulmonary hypertension, Jafari et al. uncover mechanisms underlying disease severity-associated remodeling, identify key signaling molecules involved in fibrotic and proliferative pathways, and reveal processes driving right ventricular recovery after pulmonary endarterectomy.

## Main

Right ventricular (RV) remodeling is a hallmark of pulmonary hypertension (PH), substantially affecting RV function and clinical outcomes^1,2^. A better understanding of the complex functional and structural adaptive and maladaptive processes is crucial, as RV remodeling may be reversible^3,4^. However, the molecular mechanisms driving the transition between functional and failing RV states remain poorly understood. Recent studies using omics technologies have begun to elucidate transcriptomic profiles associated with RV adaptation in pulmonary arterial hypertension (PAH, group 1 PH)^5–7^.

Chronic thromboembolic pulmonary hypertension (CTEPH), a group 4 PH subtype, arises from unresolved thromboemboli that obstruct pulmonary vessels, promoting vascular remodeling and RV failure if untreated^8–10^. The gold standard treatment for CTEPH is pulmonary endarterectomy (PEA), which is so far the only potential cure for these patients and usually leads to a far-reaching normalization of pulmonary hemodynamics, with recovery of the RV after PEA being observed in the majority of patients^4,11^. Yet, the degree of post-PEA recovery varies^12–14^, and the underlying molecular and structural mechanisms are not fully characterized.

By contrast, reverse remodeling of the left ventricle (LV) in the context of heart failure has been extensively studied. It comprises restoration of LV chamber geometry with a leftward shift of the end-diastolic pressure–volume relationship, linked with normalization of myocardial cell size and changes of various molecular, metabolic and extracellular matrix (ECM)-related properties toward physical homeostasis^15–17^.

CTEPH offers a rare opportunity to study RV remodeling in humans at the intraindividual level, with access to myocardial biopsies before and after surgical intervention^14,18,19^. In our study, RV free wall and septal biopsies from patients with CTEPH undergoing PEA (prePEA) were analyzed to investigate transcriptomic and structural remodeling compared with controls. Patients were stratified by clinical severity based on European Society of Cardiology (ESC) and European Respiratory Society (ERS) criteria, and follow-up post-PEA surgery (postPEA) allowed for intraindividual comparisons of recovery. Furthermore, RV transcriptomic profiles were compared with datasets from human left heart failure and mouse models of pulmonary artery banding (PAB) and de-banding to identify common and distinct molecular features.

## Results

### ESC/ERS-based CTEPH groups show RV structural and gene changes

**Fig. 1:**
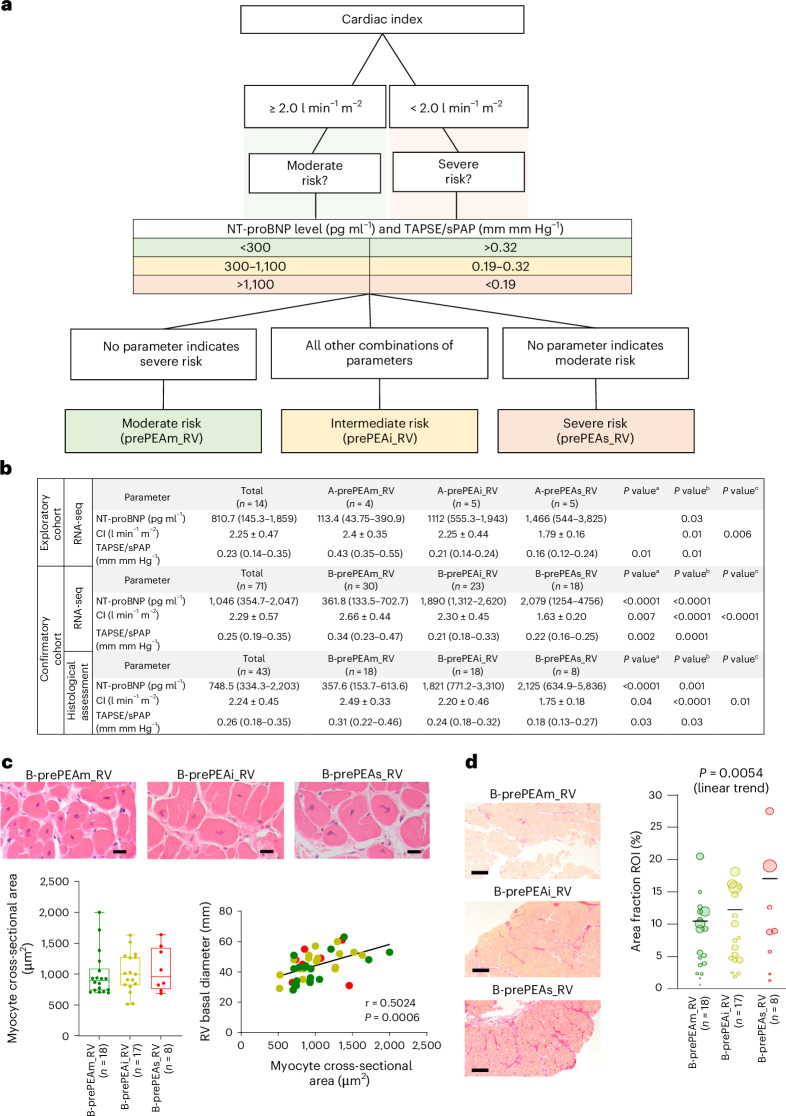
Classification of patients with CTEPH. **a**, The flowchart shows the algorithm for risk stratification in patients with CTEPH. **b**, Classification of patients with CTEPH before PEA based on their clinical parameters and ESC/ERS guidelines in the exploratory cohort (A-prePEA_RV, *n* = 14) and confirmatory cohort for RNA-seq profiling (B-prePEA_RV, *n* = 71) and for histological assessment (B-prePEA_RV, *n* = 43). Data are presented as median (IQR) (NT-proBNP and TAPSE/sPAP) or mean ± s.d. (CI), and the *P* value was calculated using the two-tailed Mann–Whitney test or unpaired two-tailed *t*-test, respectively. The *P* values correspond to the following comparisons: ^a^A-prePEAm_RV versus A-prePEAi_RV/B-prePEAm_RV versus B-prePEAi_RV, ^b^for A-prePEAm_RV versus A-prePEAs_RV/B-prePEAm_RV versus B-prePEAs_RV and ^c^for A-prePEAi_RV versus A-prePEAs_RV/B-prePEAi_RV versus B-prePEAs_RV. **c**, Representative images and quantification of the cross-sectional areas of RV myocytes within H&E-stained tissue sections were analyzed in the confirmatory cohort of patients with CTEPH at prePEA (B-prePEA_RV, *n* = 43). Data are presented as mean ± SEM, and *P* values were calculated by one-way ANOVA followed by Tukey’s multiple-comparison test. Pearson correlation was conducted between the RV basal diameter and myocyte cross-sectional area. The boxes show the IQR (25th to 75th percentile), and the central bands indicate the median. The whiskers extend to 1.5 times the IQR above and below the box. Scale bars, 20 µm. **d**, Representative images and corresponding quantification of Sirius red-stained RV sections depicting the percentage of area fraction (ROI) in different risk groups of patients with CTEPH from the confirmatory cohort. The categorization includes moderate-risk (B-prePEAm_RV, *n* = 18 patients with *n* = 79 fibrosis tissue sections), intermediate-risk (B-prePEAi_RV, *n* = 17 patients with *n* = 68 fibrosis tissue sections) and severe-risk (B-prePEAs_RV, *n* = 8 patients with *n* = 28 fibrosis tissue sections) groups. The differences in the area fractions between the different risk groups were defined with a quasi-binomial model using the weights, and the *P* value was calculated using chi-squared test. Scale bars, 100 µm.

In this study, patients with CTEPH before PEA (prePEA) were categorized into different risk groups in the exploratory (A-prePEA_RV, *n* = 14) and confirmatory (B-prePEA_RV, *n* = 88) cohorts based on their clinical parameters and the ESC/ERS guidelines^8^. Risk stratification was guided by a flowchart-based algorithm incorporating three key parameters—cardiac index (CI), N-terminal pro-brain natriuretic peptide (NT-proBNP) level, and the tricuspid annular plane systolic excursion to systolic pulmonary arterial pressure (TAPSE/sPAP) ratio—along with specific value ranges. Patients were first divided based on a CI threshold of 2.0 l min^−^^1^ m^−^^2^. In the moderate-risk group (prePEAm) (CI ≥ 2.0), neither NT-proBNP nor TAPSE/sPAP values fell within the severe-risk range (NT-proBNP > 1100 pg ml⁻¹ or TAPSE/sPAP <0.19 mm mm Hg⁻¹). In the severe-risk group (prePEAs) (CI  < 2.0), no parameter indicated moderate-risk values (NT-proBNP <300 pg ml⁻¹ or TAPSE/sPAP > 0.32 mm mm Hg⁻¹). The intermediate-risk group (prePEAi) included all other combinations that did not meet the exclusive criteria for either moderate or severe risk (Fig. 1a,b, Table 1, Supplementary Tables 1 and 2, and Methods).

**Table 1:** Clinical characteristics of the patients with CTEPH in the prePEA confirmatory cohort

Parameter	Total (*n* = 71)	B-prePEAm_RV (*n* = 30)	B-prePEAi_RV (*n* = 23)	B-prePEAs_RV (*n* = 18)	*P* value^b^	*P* value^c^	*P* value^d^
**General characteristics**							
Female gender, *n* (%)	22 (30.99)	11 (36.67)	7 (30.43)	4 (22.22)			
Age at PEA (years)	58.7 ± 14.7	57.7 ± 15	59.6 ± 14	59.5 ± 15.8			
BMI (kg m^−^²)	27.81 ± 6.01	28.18 ± 5.25	27.41 ± 7.01	27.71 ± 6.16			
**Laboratory**							
GFR (ml min^−1^ 1.73 m^−^^2^)^a^	80.29 (63.71–92.57)	89.26 (79.40–96.68)	78.33 (62.53–96.86)	71.1 (49.19–82.17)		0.007	
Creatinine (µmol l^−1^)^a^	0.96 (0.82–1.12)	0.9 (0.72–1)	1.03 (0.82–1.2)	1.14 (0.99–1.2)		0.002	
NT-proBNP (pg ml^−1^)^a^	1,046 (354.7–2,047)	361.8 (133.5–702.7)	1,890 (1,312–2,620)	2,079 (1,254–4,756)	<0.0001	<0.0001	
**Functional status**							
6-MWD (m)	415.8 ± 110.13	452.6 ± 90.85	401.7 ± 111.2	336 ± 128.7			
WHO FC, *n* (%)							
I	0 (0)	0 (0)	0 (0)	0 (0)			
II	8 (11.3)	5 (16.6)	4 (17.4)	0 (0)			
III	50 (70.4)	20 (66.6)	16 (69.6)	13 (72.2)			
IV	13 (18.3)	5 (16.7)	3 (13)	5 (27.8)			
VO_2_max (ml min^−1^ kg^−1^)	11.89 ± 4.01	15.21 ± 4.79	10.6 ± 2.8	10.13 ± 2.43	0.008	0.008	
**Hemodynamics**							
sPAP (mm Hg)	72.39 ± 19.33	63.27 ± 19.02	81.48 ± 18.13	76 ± 14.95		0.04	0.001
mPAP (mm Hg)	41.42 ± 10.21	36.07 ± 9.34	45.74 ± 9.88	44.83 ± 8.12	0.001	0.006	
mRAP (mm Hg)	7.4 ± 3.81	6.33 ± 2.63	6.84 ± 4.1	10.71 ± 4.03		0.02	
PVR (WU)	7.54 ± 3.16	5.72 ± 2.59	7.7 ± 1.98	10.33 ± 3.14	0.03	<0.0001	0.008
CI (l min^−1^ m^−2^)	2.29 ± 0.57	2.66 ± 0.44	2.30 ± 0.45	1.63 ± 0.20	0.007	<0.0001	<0.0001
CO (l min^−1^)	4.54 ± 1.24	5.30 ± 1.13	4.45 ± 0.93	3.47 ± 0.78	0.02	<0.0001	0.002
**Echocardiography**							
RV basal diameter (mm)	44.25 ± 8.1	38.83 ± 9.96	47.45 ± 8.4	49.35 ± 11.42	0.002	0.0006	
LVEF (%)^a^	60 (55–60)	60 (55–60)	57 (55–60)	55 (55–60)			
TAPSE (mm)^a^	19 (16–23)	20.5 (19–24.5)	18 (15–22)	15.5 (11.75–18.25)		<0.0001	
TAPSE/sPAP (mm mm Hg^−1^)^a^	0.25 (0.19–0.35)	0.34 (0.23–0.47)	0.21 (0.18–0.33)	0.22 (0.16–0.25)	0.002	0.0001	

Data are presented as *n* (%), median (IQR) or mean ± s.d.

BMI, body mass index; GFR, glomerular filtration rate; 6-MWD, 6-min-walk-test distance; WHO FC, World Health Organization Functional Class; WU, wood units; LVEF, left ventricular ejection fraction.

^a^Median (IQR).

^b^B-prePEAm_RV versus B-prePEAi_RV.

^c^B-prePEAm_RV versus B-prePEAs_RV.

^d^B-prePEAi_RV versus B-prePEAs_RV.

**Extended Data Fig. 1:**
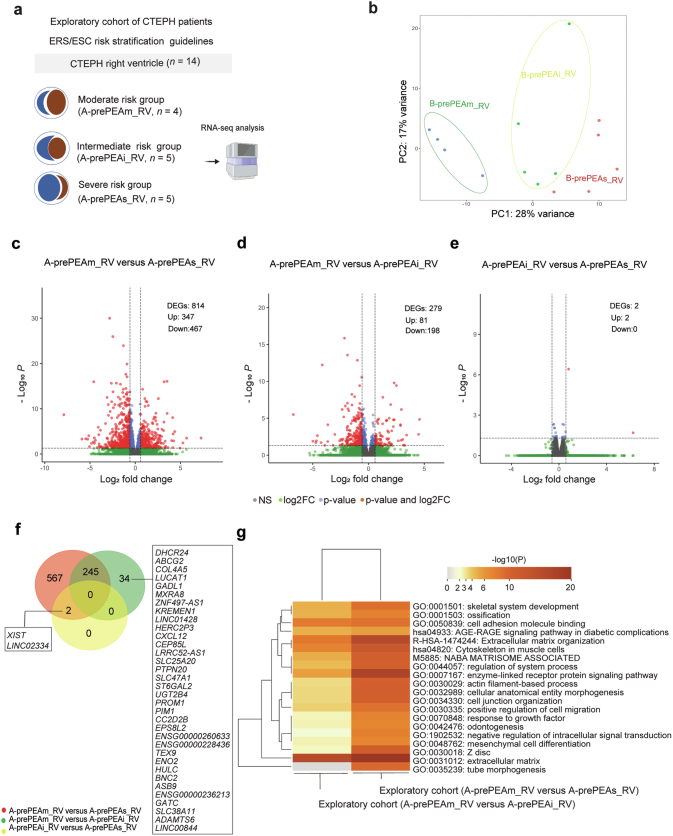
Transcriptomic profiling of the right ventricle in the exploratory cohort of patients with CTEPH. **a**, In the exploratory cohort, RNA-seq analysis was performed on the RV of patients with CTEPH before PEA (A-prePEA_RV, *n* = 14), and patients were classified into three risk groups, including A-prePEAm_RV (*n* = 4), A-prePEAi_RV (*n* = 5), and A-prePEAs_RV (*n* = 5). Illustration created using Biorender.com. **b**, Principal component analysis (PCA) plot depicting the RV transcriptomic profile from 14 samples. The colors represent the distinct status of the RV. Volcano plot showing the distribution of each gene in **c**, A-prePEAm_RV versus A-prePEAs_RV, **d**, A-prePEAm_RV versus A-prePEAi_RV, and **e**, A-prePEAi_RV versus A-prePEAs_RV, of the exploratory cohort of patients with CTEPH. The significant differentially expressed genes (DEGs) ( | log2FC | ≥ 0.585 and *P*_adj_ ≤ 0.05, two-sided) were discriminated based on the *P* value < 0.05 and |log2FC | >0.585 (in red), only *P* value (blue), log2FC (green), and not significant in grey dots. **f**, Venn diagrams of DEGs display the common and distinct DEGs among the three groups of A-prePEAm_RV versus A-prePEAs_RV, A-prePEAm_RV versus A-prePEAi_RV, and A-prePEAi_RV versus A-prePEAs_RV in the exploratory cohort of patients with CTEPH. **g**, Heatmap shows the top 20 overrepresented clusters of terms for all the DEGs between the following comparisons: A-prePEAm_RV versus A-prePEAi_RV and A-prePEAm_RV versus A-prePEAs_RV. Source data

In the confirmatory cohort, RV histology revealed a significant correlation between myocyte cross-sectional area and RV basal diameter (Fig. 1c and Supplementary Table 2). Notably, the severe-risk group showed increased RV fibrosis compared with the intermediate- and moderate-risk groups (Fig. 1d and Supplementary Table 2). To determine whether the ESC/ERS-based subgrouping of patients with CTEPH also correlates with transcriptomic changes in the right ventricle, RNA-sequencing (RNA-seq) was performed on the RV biopsies of the exploratory cohort, that is, A-prePEA_RV before PEA (Extended Data Fig. 1a and Supplementary Table 1). Principal component analysis (PCA) revealed clear separation among the risk groups, indicating transcriptomic divergence (Extended Data Fig. 1b and Supplementary Fig. 1). Differential gene expression analysis identified 814 dysregulated genes in the severe-risk group and 279 in the intermediate-risk group compared with the moderate-risk group (Extended Data Fig. 1c–e). Of these, 34 and 567 genes were uniquely expressed in the intermediate-risk and severe-risk groups, respectively (Extended Data Fig. 1f).

The top 50 differentially expressed genes (DEGs) in the severe-risk and intermediate-risk groups compared with the moderate-risk group include the heart-disease-associated genes, that is, proenkephalin (*PENK*), transforming growth factor beta 2 (*TGFB2*) and natriuretic peptide precursor A (*NPPA*) and B (*NPPB*). ECM-related genes include tenascin-C (*TNC*), periostin (*POSTN*) as a secreted ECM protein and collagen type IX alpha 1 chain *(COL9A1)* (Supplementary Fig. 2a,b). Gene Ontology (GO) enrichment analysis highlighted ECM-related terms among the top 20 overrepresented clusters of terms in the intermediate-risk and severe-risk groups, including ‘cell adhesion molecule binding’, ‘AGE-RAGE signaling pathway’, ‘cytoskeleton in muscle cells’, ‘NABA MATRISOME ASSOCIATED’, ‘enzyme-linked receptor protein signaling pathway’ and ‘extracellular matrix’ (Extended Data Fig. 1g and Supplementary Fig. 3). Additional pathways such as ‘response to estradiol’, ‘response to hypoxia’ and ‘regulation of cytoskeletal organization’ were enriched exclusively in the severe-risk group (Supplementary Fig. 3).

### RV gene changes by risk group in the CTEPH confirmatory cohort

**Fig. 2:**
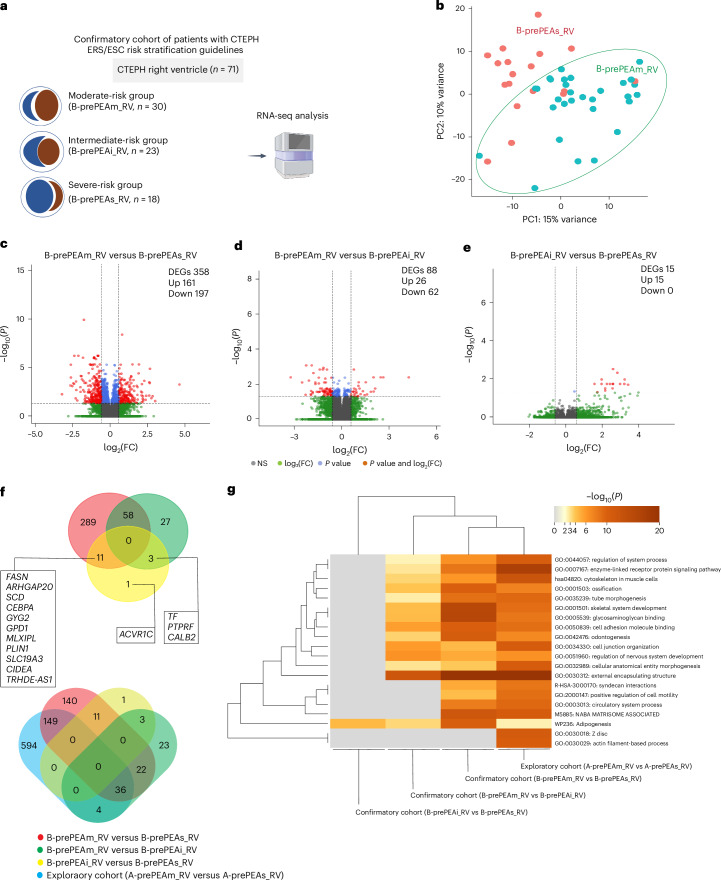
Transcriptomic profiling of the right ventricle in the confirmatory cohorts of patients with CTEPH. **a**, RNA-seq analysis was performed on the RV tissue of patients with CTEPH (*n* = 71) in the confirmatory cohort. Patients were stratified into moderate-risk (B-prePEAm_RV, *n* = 30), intermediate-risk (B-prePEAi_RV, *n* = 23) and severe-risk (B-prePEAs_RV, *n* = 18) groups based on their clinical parameters and the ESC/ERS guidelines. **b**, PCA plot illustrating the RV transcriptomic profiles of patients in the B-prePEAm_RV and B-prePEAs_RV groups. Each patient in the B-prePEAs_RV group is represented by a red circle, and each patient in the B-prePEAm_RV group is represented by a green circle. **c**–**e**, Volcano plots showing the distribution of the genes in B-prePEAm_RV versus B-prePEAs_RV (**c**), B-prePEAm_RV versus B-prePEAi_RV (**d**) and B-prePEAi_RV versus B-prePEAs_RV (**e**). The significant DEGs (base mean ≥ 5; |log_2_(FC)| ≥ 0.585; *P*-adjusted value (*P*_adj_ ) ≤ 0.05, two sided) were discriminated based on *P* < 0.05 and |log_2_(FC)| > 0.585 (red dots), with genes solely significant in *P* value represented by blue dots, those solely significant in log_2_(FC) represented by green dots and non-significant (NS) genes represented by gray dots. **f**, Venn diagrams of DEGs illustrate the common and distinct DEGs among the three groups of B-prePEAm_RV versus B-prePEAs_RV, B-prePEAm_RV versus B-prePEAi_RV and B-prePEAi_RV versus B-prePEAs_RV in the confirmatory cohort of patients with CTEPH. The counts of overlapping and distinct DEGs across four groups (A-prePEAm_RV versus A-prePEAs_RV, B-prePEAm_RV versus B-prePEAs_RV, B-prePEAi_RV versus B-prePEAs_RV and B-prePEAm_RV versus B-prePEAi_RV) are shown. **g**, Heatmap showing the top 20 overrepresented clusters of terms for all the DEGs between the following comparisons: B-prePEAi_RV versus B-prePEAs_RV, B-prePEAm_RV versus B-prePEAi_RV, B-prePEAm_RV versus B-prePEAs_RV and A-prePEAm_RV versus A-prePEAs_RV. Panel **a** created with BioRender.com. Source data

To validate the association between clinical parameters and transcriptional changes observed in the exploratory cohort, we performed RNA-seq on the RV of 71 patients out of 88 in the confirmatory cohort. Patients were again stratified into moderate-risk (B-prePEAm_RV, *n* = 30), intermediate-risk (B-prePEAi_RV, *n* = 23) and severe-risk (B-prePEAs_RV, *n* = 18) groups based on the same scoring system (Figs. 1a,b and 2a, and Table 1). Compared with the moderate-risk group, the intermediate- and severe-risk groups showed increased NT-proBNP, sPAP, mean pulmonary artery pressure (mPAP), pulmonary vascular resistance (PVR) and RV basal diameter, and reduced maximum oxygen consumption (VO_2_max), CI, cardiac output (CO) and TAPSE/sPAP (Table 1).

PCA separated moderate- and severe-risk groups, while the intermediate-risk group showed greater heterogeneity (Fig. 2b). DEG analysis revealed 358 and 88 dysregulated genes in the severe- and intermediate-risk groups, respectively, compared with the moderate-risk group. Only 15 DEGs were found between the intermediate- and severe-risk groups, suggesting limited transcriptomic divergence despite clinical differences (Fig. 2c–e and Table 1).

**Extended Data Fig. 2:**
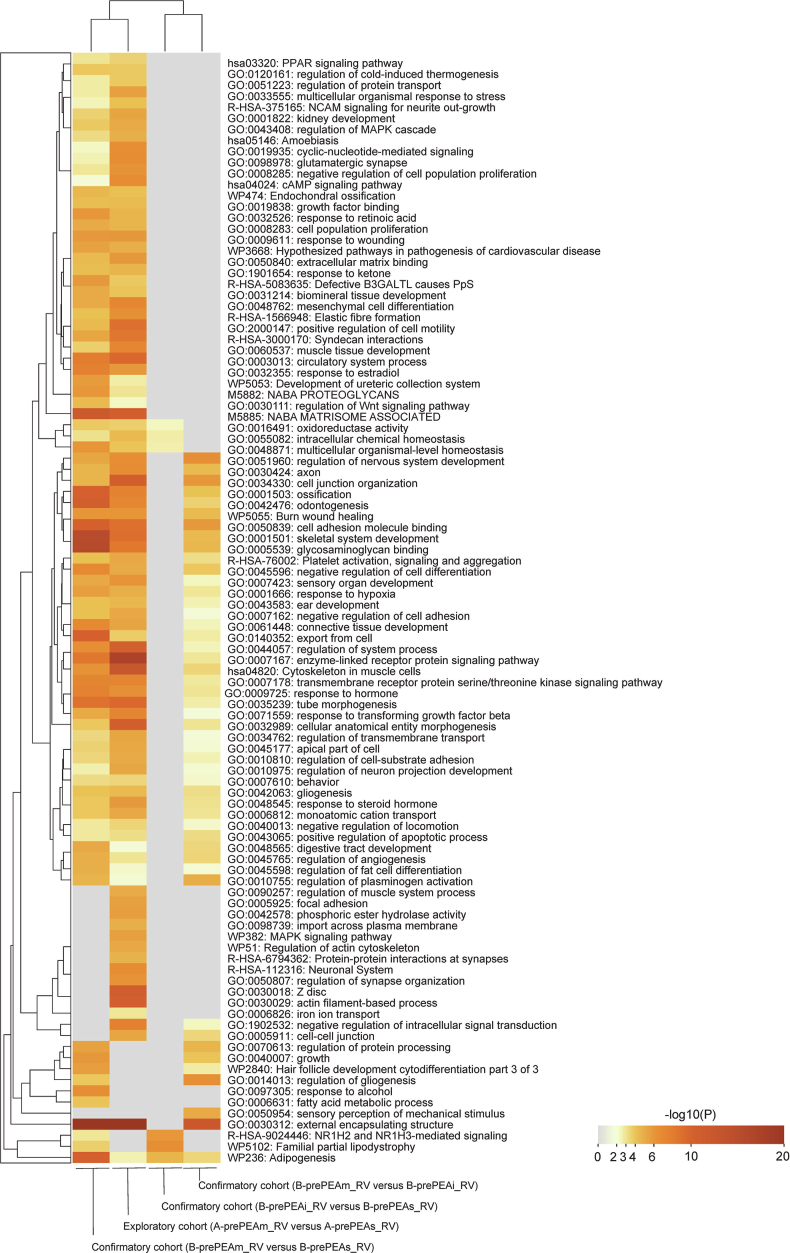
Pathway enrichment analysis in the RV of the exploratory and confirmatory cohorts of patients with CTEPH before PEA. The clustered heatmap shows top 100 overrepresented GO terms and pathways for both cohorts in following comparisons: B-prePEAm_RV versus B-prePEAs_RV, A-prePEAm_RV versus A-prePEAs_RV, B-prePEAi_RV versus B-prePEAs_RV, and B-prePEAm_RV versus B-prePEAi_RV. Source data

Venn diagrams and GO term enrichment analysis for both cohorts showed shared and unique genes and pathways. GO terms enriched in the severe-risk group included ‘enzyme-linked receptor protein signaling pathway’, ‘cytoskeleton’, ‘positive regulation of cell motility’, ‘NABA MATRISOME ASSOCIATED’ and ‘adipogenesis’—consistent with findings from the exploratory cohort (Fig. 2f,g and Extended Data Fig. 2). Several highly regulated genes within these GO terms—including *COMP* (cartilage oligomeric matrix protein), *COLQ* (acetylcholinesterase collagenic tail peptide), *CD38* (cluster of differentiation 38), *SEZ6L* (seizure related 6 homolog like), *CDH11* (cadherin 11), *CCN2* (cellular communication network factor 2), *CCDC80* (coiled-coil domain-containing protein 80), *CRACD* (capping protein inhibiting regulator of actin dynamics), *SERPINE1* (serpin family E member 1), *LTBP2* (latent transforming growth factor beta binding protein 2), *NPPB* and *NPPA*—show an association with disease severity.

### CTEPH versus control and LHF reveals RV failure-related genes

**Fig. 3:**
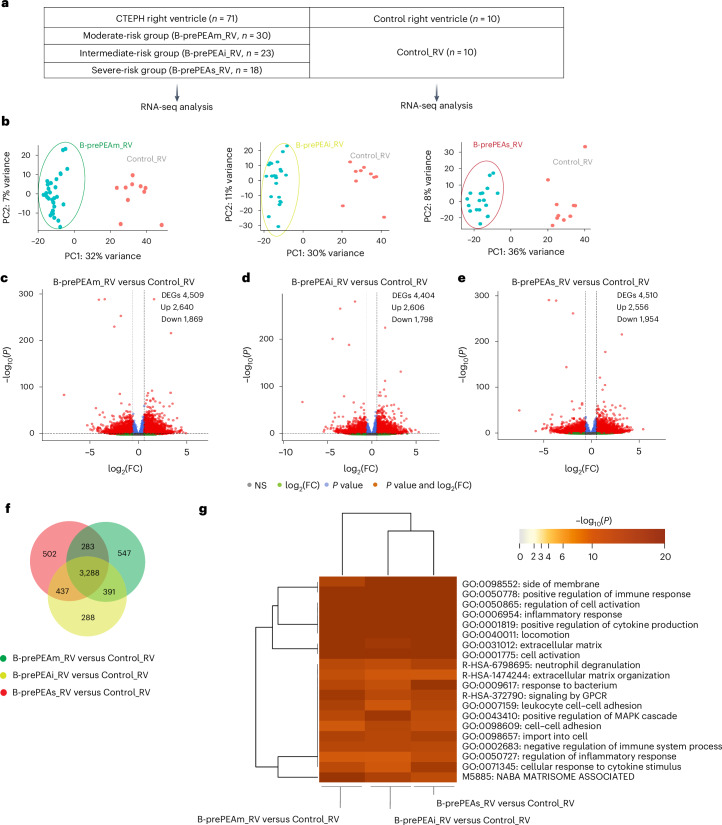
Comparing the transcriptomic profiling of the right ventricle in patients with CTEPH of the confirmatory cohort with that of the control right ventricle. **a**, In the confirmatory cohort of patients with CTEPH, RNA-seq was performed on RV tissue (B-prePEA_RV, *n* = 71). The patients at prePEA were classified based on their clinical parameters and ESC/ERS guidelines into B-prePEAm_RV (*n* = 30), B-prePEAi_RV (*n* = 23) and B-prePEAs_RV (*n* = 18). In addition, RNA-seq analysis was performed on Control_RV (*n* = 10). **b**, PCA plots depicting the RV transcriptomic profiles in the following groups: B-prePEAm_RV (*n* = 30) and Control_RV (*n* = 10), B-prePEAi_RV (*n* = 23) and Control_RV (*n* = 10), and B-prePEAs_RV (*n* = 18) and Control_RV (*n* = 10) for each comparison. In each PCA plot, the encircled blue dots represent the right ventricles of patients with CTEPH, while the Control_RVs are shown with red dots. **c**–**e**, Volcano plot representing the gene distribution for different comparisons: B-prePEAm_RV versus Control_RV (**c**), B-prePEAi_RV versus Control_RV (**d**) and B-prePEAs_RV versus Control_RV (**e**). The significant DEGs were selected based on the following criteria: base mean ≥ 5; |log_2_(FC)| ≥ 0.585; *P*_adj_ ≤ 0.05, two-sided. Classification of the significant DEGs was performed based on *P* < 0.05 and |log_2_(FC)| > 0.585 (indicated by red dots), only *P* value (indicated by blue dots), log_2_(FC) (indicated by green dots) and not significant (indicated by gray dots). **f**, Venn diagram showing the overlap and distinct DEGs between three distinct groups: B-prePEAm_RV versus Control_RV, B-prePEAi_RV versus Control_RV and B-prePEAs_RV versus Control_RV. **g**, Heatmap visualizing the top 20 overrepresented clusters of terms among various comparisons: B-prePEAm_RV versus Control_RV, B-prePEAi_RV versus Control_RV and B-prePEAs_RV versus Control_RV. Source data

To explore molecular mechanisms of RV remodeling in CTEPH, transcriptomic profiles of the B-prePEA_collective RV cohort (*n* = 71; moderate *n* = 30, intermediate *n* = 23 and severe *n* = 18) were compared with those of a normal control group (Control_RV, *n* = 10) (Fig. 3a). PCA plots showed clear separation between all prePEA subgroups and Control_RV (Fig. 3b). Global gene expression correlations are shown in Supplementary Fig. 4a–c. RNA-seq revealed over 4,000 DEGs in all prePEA subgroups compared with controls (Fig. 3c–e). Venn diagrams showed both shared and unique DEGs in each group (Fig. 3f and Supplementary Fig. 5a–c), with 547, 288 and 502 genes uniquely regulated in the moderate-, intermediate- and severe-risk groups, respectively.

**Extended Data Fig. 3:**
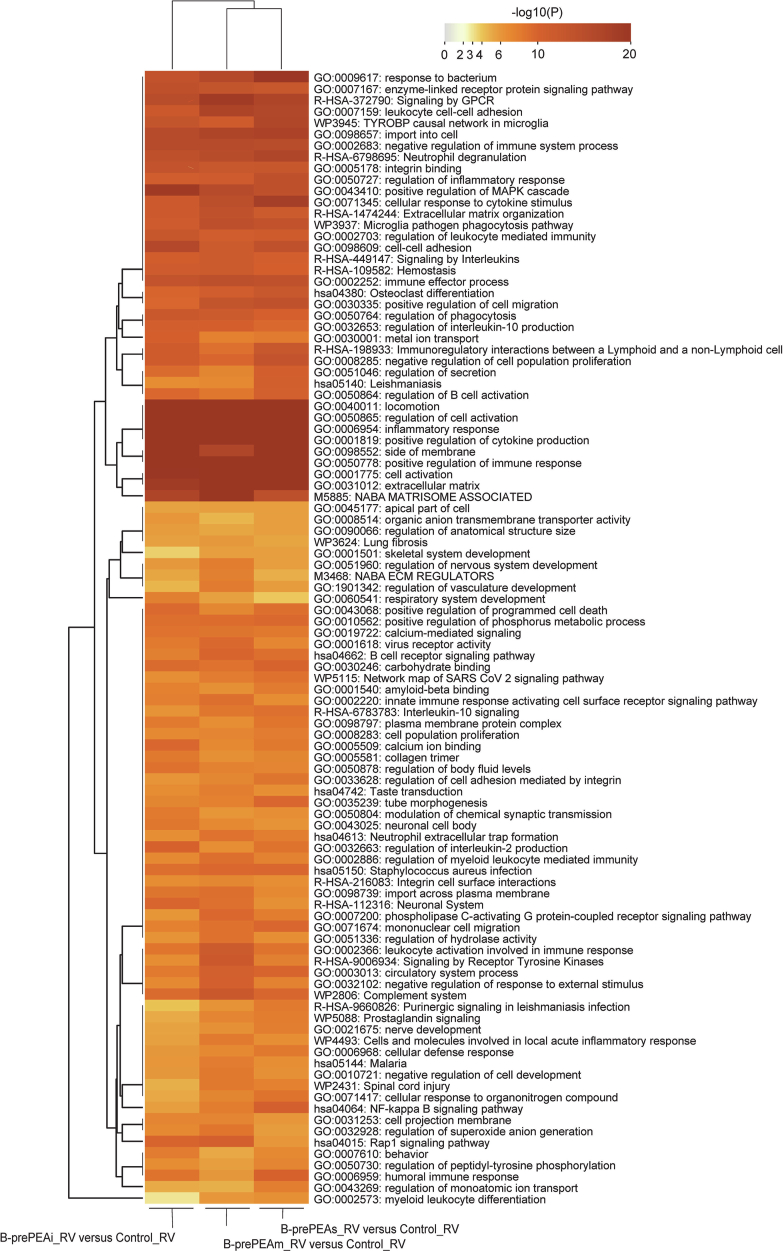
Pathway enrichment analysis in the CTEPH RV of the confirmatory cohort versus control RV. The clustered heatmap displays the top 100 overrepresented GO terms and pathways resulting from the comparisons between B-prePEAi_RV versus Control_RV, B-prePEAm_RV versus Control_RV, and B-prePEAs_RV versus Control_RV. Source data

GO analysis identified enriched terms such as ‘NABA MATRISOME ASSOCIATED’, ‘extracellular matrix’, ‘regulation of cell activation’, ‘positive regulation of immune response’, ‘cellular response to cytokine stimulus’ and ‘neutrophil degranulation’ in prePEA subgroups compared with Control_RV (Fig. 3g and Extended Data Fig. 3). Notably, inflammatory-related GO terms showed increased log fold changes (FCs) with disease severity.

To assess RV failure-specific transcriptional signatures, data from two public heart failure studies were incorporated. From GSE11625020 (ref. ^20^), we reanalyzed normalized counts from LV tissue of patients with dilated (*n* = 37) or ischemic cardiomyopathy (*n* = 13) versus non-failing controls (*n* = 14) using R package limma^21^ with the default setting. These were compared with RV samples from the confirmatory cohort (B-prePEAs_RV versus B-prePEAm_RV), processed identically. Gene expression profiles showed a modest but significant correlation (*r* = 0.39 log_2_(FC), *P* < 0.0001), with 57 DEGs overlapping (≥2-fold, false discovery rate (FDR) ≤ 5%) and 4 (7%) regulated in opposite directions (Supplementary Fig. 6a).

In addition, comparison with a meta-analysis of 16 left heart failure (LHF) studies^22^ revealed that 143 genes (∼40%) overlapped with right ventricular failure/right ventricular hypertrophy (RVF/RVH) DEGs, indicating that right heart failure (RHF) transcriptional responses are largely distinct from those in LHF (Supplementary Fig. 6b).

### Septum shows molecular alterations pre- and post-PEA

**Fig. 4:**
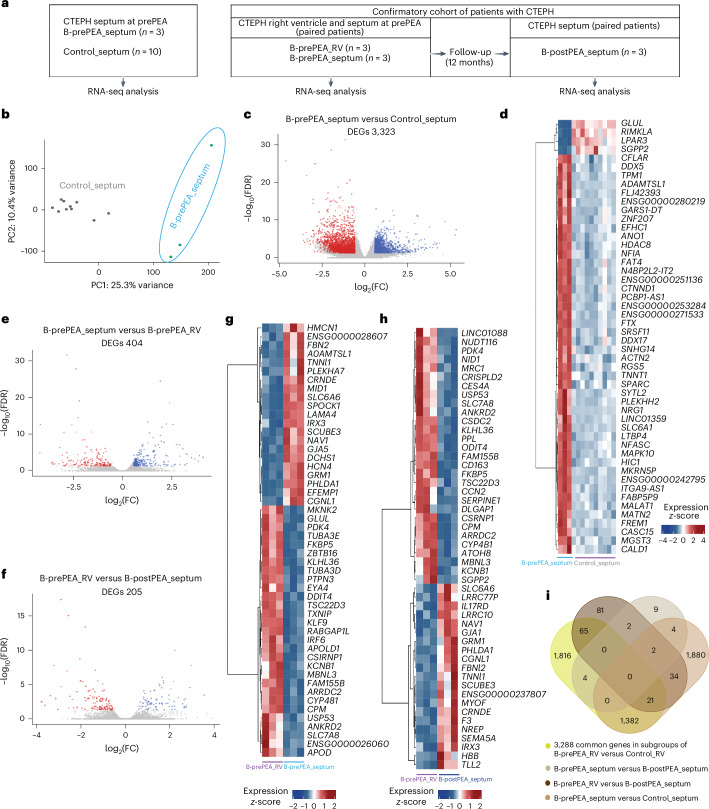
Analysis of the transcriptomic profile of the septum before PEA and comparing it with the control septum and after PEA. **a**, In the confirmatory cohort, RNA-seq analysis was performed on the septum of patients with CTEPH before PEA (B-prePEA_septum, *n* = 3) and the control septum (Control_septum, *n* = 10). Furthermore, RV biopsies were obtained before PEA (B-prePEA_RV, *n* = 3) and septum biopsies (B-postPEA_septum, *n* = 3) 12 months after PEA from the same patients. **b**, PCA plot illustrating the septum before PEA (B-prePEA_septum) and the control septum (Control_septum). **c**, Volcano plot showing significant DEGs (base mean ≥ 5; |log_2_(FC)| ≥ 0.585; FDR ≤ 0.05) in the B-prePEA_septum (red) versus the Control_septum (blue). **d**, Heatmap showing the top 50 significant DEGs in B-prePEA_septum versus Control_septum. The scaled *z*-score of normalized counts is shown. **e**,**f**, Volcano plot showing the significant DEGs (base mean ≥ 5; |log_2_(FC)| ≥ 0.585; FDR ≤ 0.05) in B-prePEA_septum (red) versus B-prePEA_RV (blue) (**e**) and B-prePEA_RV (red) versus B-postPEA_septum (blue) (**f**). **g**,**h**, Heatmap showing the top 50 significant DEGs in B-prePEA_septum versus B-prePEA_RV (**g**) and B-prePEA_RV versus B-postPEA_septum (**h**). The scaled *z*-score of normalized counts is shown. **i**, Venn diagram illustrating the common and unique DEGs between the four groups (B-prePEA_RV versus Control_RV, B-prePEA_septum versus B-postPEA_septum, B-prePEA_RV versus B-postPEA_septum and B-prePEA_septum versus Control_septum). Source data

To determine the effect of PEA on the gene expression profile of the septum, septal biopsies were taken using catheters placed in the RV cavity of three pre-PEA patients of the confirmatory cohort. The expression profiles of these samples (B-prePEA_septum) were compared with those of control septa (Control_septum, *n* = 10) (Fig. 4a). PCA revealed clear separation between B-prePEA_septum and Control_septum. RNA-seq identified 3,323 DEGs in the B-prePEA_septum versus controls (Fig. 4b–d), indicating interventricular septal remodeling due to RV pressure overload in CTEPH.

Next, we compared (1) septum and RV free wall gene expression at baseline, (2) septum changes pre- versus post-PEA and (3) overall RV and septal remodeling. We analyzed RNA-seq data from matched septal and RV biopsies (Supplementary Table 3). There were 404 DEGs between B-prePEA_septum and B-prePEA_RV, and 205 DEGs between B-prePEA_RV and B-postPEA_septum (Fig. 4e,f). The top 50 DEGs for each comparison are shown in Fig. 4g,h. A Venn diagram revealed 1,400 co-regulated genes out of 3,288 in the RV and septum at baseline compared with the control (Fig. 4i).

**Extended Data Fig. 4:**
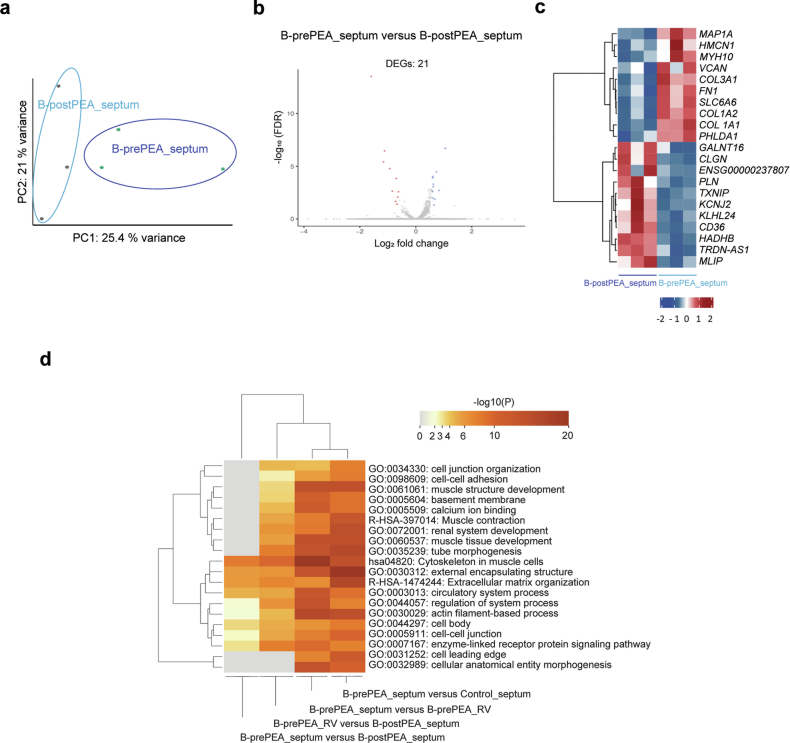
Analysis of the transcriptomic profile of the septum before and after PEA in the confirmatory cohort of patients with CETPH. **a**, PCA plot illustrates the septum before PEA (B-prePEA_septum, *n* = 3) and after PEA (B-postPEA_septum, *n* = 3). **b**, Volcano plot displays the significant DEGs (base mean ≥5; |log2FC | ≥ 0.585; FDR ≤ 0.05) in B-prePEA_septum (red) versus B-postPEA_septum (blue). **c**, Heatmap shows the top significant DEGs in B-prePEA_septum versus B-postPEA_septum. The scaled z-score of normalized counts is shown. **d**, The heatmap illustrates the top 20 overrepresented clusters of terms associated with DEGs in the following comparisons: B-prePEA_septum versus B-postPEA_septum, B-prePEA_RV versus B-postPEA_septum, B-prePEA_septum versus B-prePEA_RV, and B-prePEA_septum versus Control_septum. Source data

**Extended Data Fig. 5:**
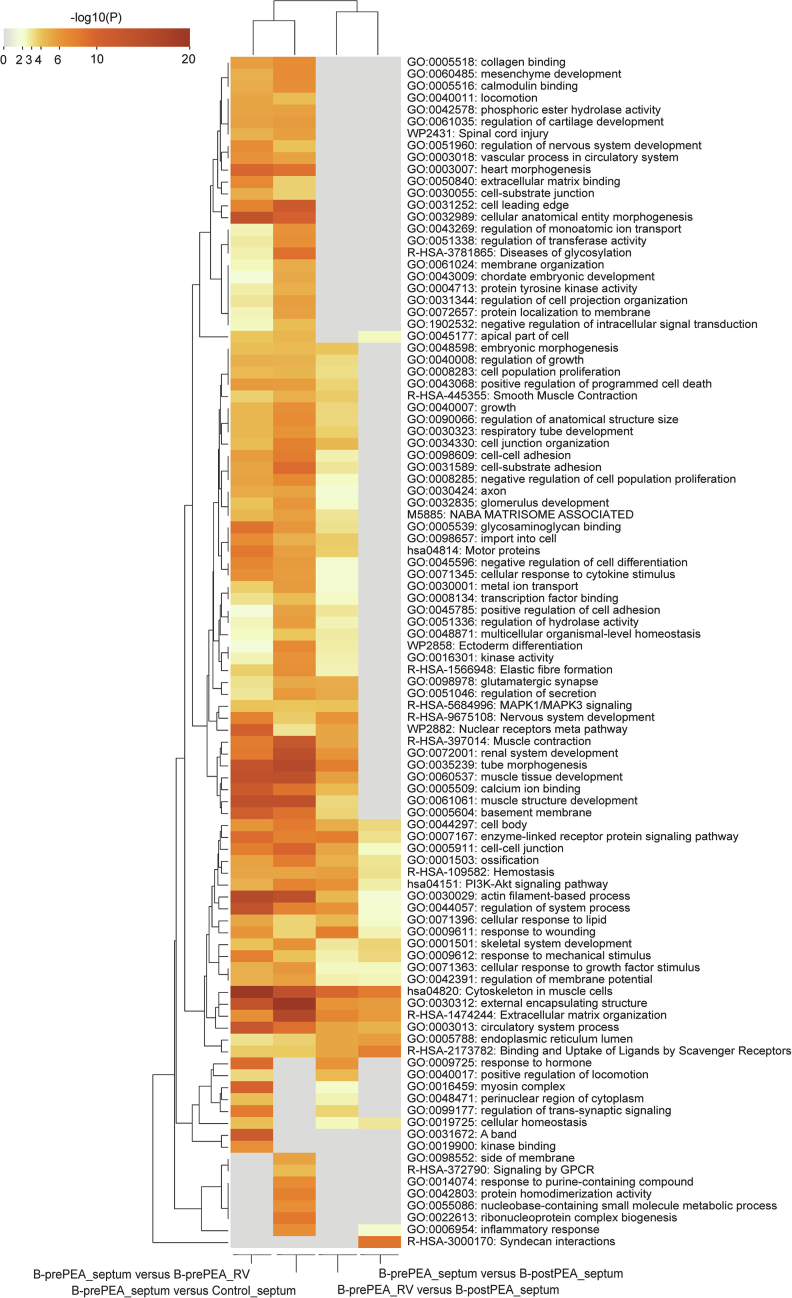
Pathway enrichment analysis in the CTEPH septum of the confirmatory cohort versus control septum. The clustered heatmap displays the top 100 overrepresented GO terms and pathways resulting from the comparisons between B-prePEA_septum versus B-prePEA_RV, B-prePEA_septum versus Control_septum, B-prePEA_RV versus B-postPEA_septum, B-prePEA_septum versus B-postPEA_septum. Source data

Post-PEA, 21 DEGs were found in the septum versus pre-PEA, with changes in ECM and cytoskeletal terms, but not in muscle contraction or cell adhesion pathways (Extended Data Fig. 4a–d). Pathway analysis indicated strong enrichment of ECM-related and cardiac muscle development pathways in both the septum and right ventricle before PEA, including actin filament processes, cytoskeleton organization and muscle contraction (Extended Data Figs. 4d and 5).

### Intraindividual RNA-seq reveals ECM and immune changes post-PEA

**Fig. 5:**
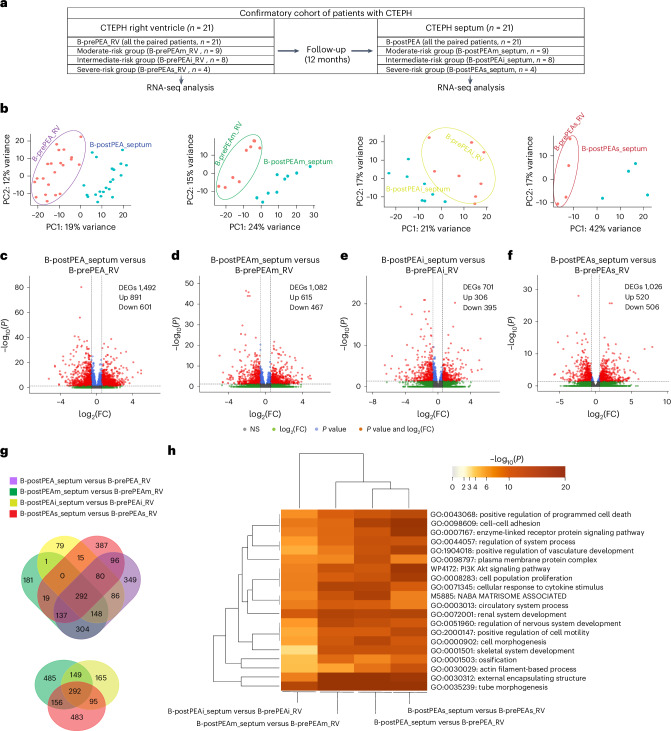
Comparing the transcriptomic profiling of the right ventricle and septum in the confirmatory cohort of patients with CTEPH before and after PEA. **a**, In the confirmatory cohort, gene expression profiles of paired B-prePEA_RV and B-postPEA_septum samples were compared. RNA-seq analysis was performed on 21 RV and septum tissues of patients with CTEPH. Patients at prePEA were categorized into moderate-risk (B-prePEAm_RV, *n* = 9), intermediate-risk (B-prePEAi_RV, *n* = 8) and severe-risk (B-prePEAs_RV, *n* = 4) groups based on their clinical parameters and ESC/ERS guidelines. After 12 months, postPEA_septum biopsies were obtained from the septum tissues of the same patients, including B-postPEAm_septum (*n* = 9), B-postPEAi_septum (*n* = 8) and B-postPEAs_septum (*n* = 4). **b**, PCA plots depicting the RV and septum transcriptomic profiles of patient-paired samples in the following groups: B-prePEA_RV (*n* = 21) and B-postPEA_septum (*n* = 21), B-prePEAm_RV (*n* = 9) and B-postPEAm_septum (*n* = 9), B-prePEAi_RV (*n* = 8) and B-postPEAi_septum (*n* = 8), and B-prePEAs_RV (*n* = 4) and B-postPEAs_septum (*n* = 4). The encircled red dots represent the distinct status of the right ventricle, while the corresponding septum is shown using blue dots. **c**–**f**, Volcano plots showing the distribution of each gene in B-postPEA_septum versus B-prePEA_RV (**c**), B-postPEAm_septum versus B-prePEAm_RV (**d**), B-postPEAi_septum versus B-prePEAi_RV (**e**) and B-postPEAs_septum versus B-prePEAs_RV (**f**). The significant DEGs (base mean expression ≥ 5; |log_2_(FC)| ≥ 0.585; *P*_adj_ ≤ 0.05, two sided) were classified based on *P* < 0.05 and |log_2_(FC)| > 0.585 (red dots), only *P* value (blue dots), log_2_(FC) (green dots) and not significant (gray dots). **g**, Top: Venn diagram illustrating the shared and distinct DEGs in four sets as follows: B-postPEA_septum versus B-prePEA_RV, B-postPEAm_septum versus B-prePEAm_RV, B-postPEAi_septum versus B-prePEAi_RV, and B-postPEAs_septum versus B-prePEAs_RV. Bottom: the common and distinct DEGs among three groups—B-postPEAm_septum and B-prePEAm_RV, B-postPEAi_septum and B-prePEAi_RV, and B-postPEAs_septum and B-prePEAs_RV—are highlighted. **h**, Heatmap illustrating the top 20 overrepresented clusters of terms for all the DEGs between several comparisons: B-postPEAi_septum versus B-prePEAi_RV, B-postPEAm_septum versus B-prePEAm_RV, B-postPEA_septum versus B-prePEA_RV, and B-postPEAs_septum versus B-prePEAs_RV. Source data

As due to clinical restrictions a direct comparison between prePEA and postPEA septal biopsies could be performed only in three patients with CTEPH, we extended this study to another 21 patients with CTEPH in whom the RV free wall biopsy was taken before PEA (B-prePEA_RV) and the septum biopsy was taken 12 months after PEA (B-postPEA_septum). PEA significantly reduced mPAP, PVR, sPAP, RV basal diameter and NT-proBNP, while the TAPSE/sPAP ratio and CO increased (Fig. 5a and Supplementary Table 4).

A global gene expression correlation matrix is shown in Supplementary Fig. 7. Patients were stratified into moderate-, intermediate- and severe-risk groups per ESC/ERS guidelines (Fig. 5a). A PCA showed clear separation between the right ventricle and septum by PC1 (Fig. 5b). RNA-seq revealed 1,492 DEGs between B-postPEA_septum and B-prePEA_RV (Fig. 5c). A comparison of postPEA septum samples with prePEA RV samples identified 1,082, 701 and 1,026 DEGs in the moderate-, intermediate- and severe-risk groups, respectively (Fig. 5d–f). Venn diagrams show 292 commonly regulated DEGs across all subgroups (Fig. 5g), with the top 50 DEGs shown in Supplementary Fig. 8a–d.

**Extended Data Fig. 6:**
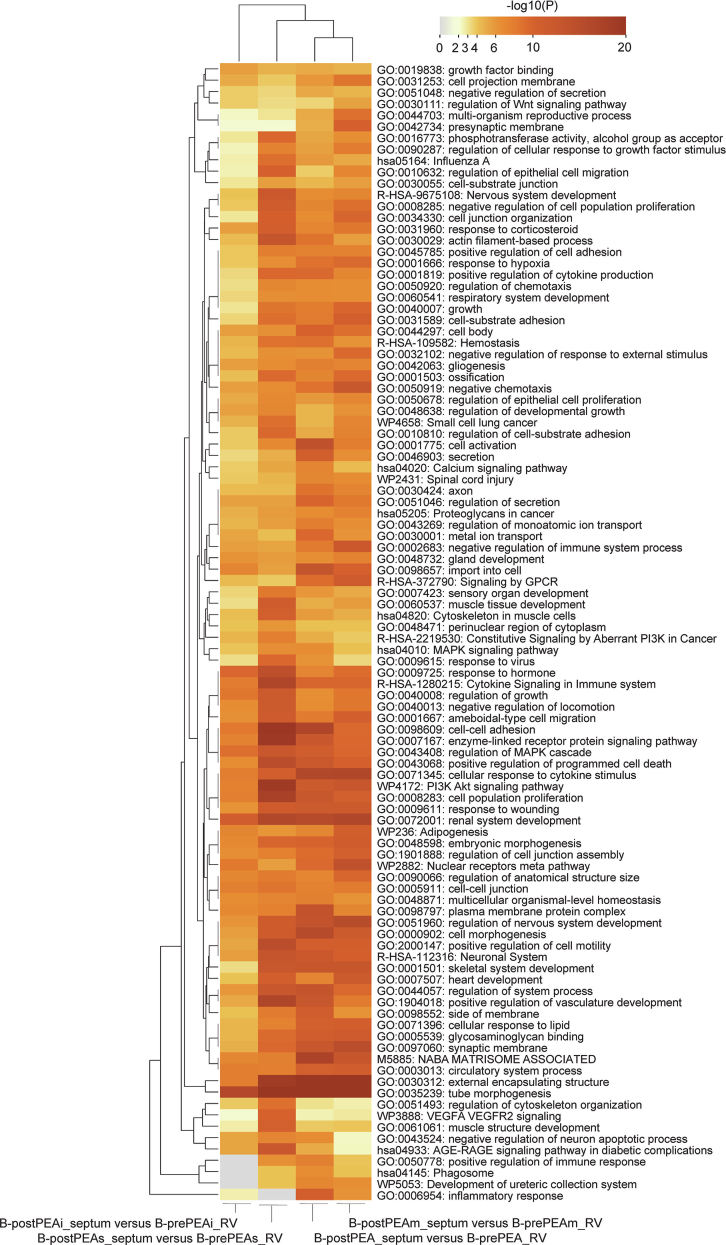
Pathway enrichment analysis in the confirmatory cohort of CTEPH patients before and after PEA. The clustered heatmap shows the top 100 overrepresented GO terms and pathways resulting from four comparisons, including: B-postPEAi_septum versus B-prePEAi_RV, B-postPEAs_septum versus B-prePEAs_RV, B-postPEA_septum versus B-prePEA_RV, and B-postPEAm_septum versus B-prePEAm_RV. Source data

Pathway enrichment in B-postPEA_septum versus B-prePEA_RV revealed upregulation of tube morphogenesis, ECM remodeling, cell motility, actin filament processes, PI3K-Akt signaling and positive regulation of cell death. By contrast, immune-related pathways such as phagosome and inflammatory response were absent in B-postPEAi_septum versus B-prePEAi_RV (Fig. 5h and Extended Data Fig. 6), suggesting gene expression changes consistent with reverse remodeling after PEA.

**Extended Data Fig. 7:**
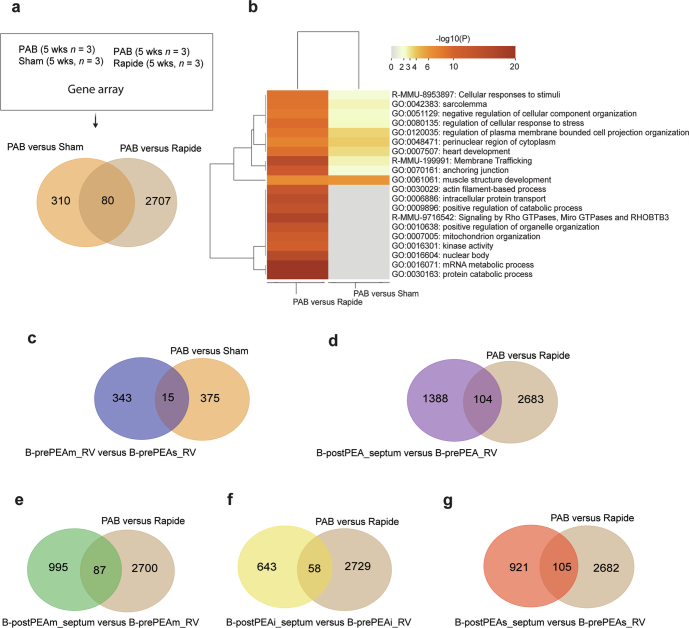
Gene expression profile in the RV of the PAB banding and de-banding mouse model. **a**, Venn diagram shows the common and specific genes in PAB versus Sham and PAB versus Rapide. **b**, Heatmap visualizes the top 20 overrepresented clusters of terms for all differentially expressed genes among PAB versus Sham and PAB versus Rapide. **c-g**, Venn diagrams showing the common and specific genes among (**c**) B-prePEAm_RV versus B-prePEAs_RV and PAB versus Sham, (**d**) B-postPEA_septum versus B-prePEA_RV and PAB versus Rapide, (**e**) B-postPEAm_septum versus B-prePEAm_RV and PAB versus Rapide, (**f**) B-postPEAi_septum versus B-prePEAi_RV and PAB versus Rapide, and, (**g**) B-postPEAs_septum versus B-prePEAs_RV and PAB versus Rapide. The source data for the re-analyzed data is from ref. ^23^. Source data

To explore whether reverse remodeling is linked to pressure unloading, we compared these data with mouse models of PAB and de-banding. Mice underwent sham surgery (Sham, *n* = 3), PAB with chronic RV pressure overload (*n* = 3) or gradual pressure unloading (De-PAB or Rapide, *n* = 3)^23^. Shared and specific DEGs and pathways between PAB versus Sham and PAB versus Rapide are shown in Extended Data Fig. 7a,b and Supplementary Fig. 9 (|log_2_(FC)| ≥ 2, *P* ≤ 0.01). Muscle structure development was enriched in both comparisons. However, 2,707 DEGs in PAB versus Rapide revealed GO terms related to organelle organization, mRNA metabolism and mitochondrial and nuclear catabolism, indicating broad metabolic changes during unloading. Comparing these mouse data with human CTEPH datasets revealed that ~8–10% of DEGs were shared post-PEA and post-de-banding (Extended Data Fig. 7c–g).

### Intraindividual histology shows reverse remodeling post-PEA

**Extended Data Fig. 8:**
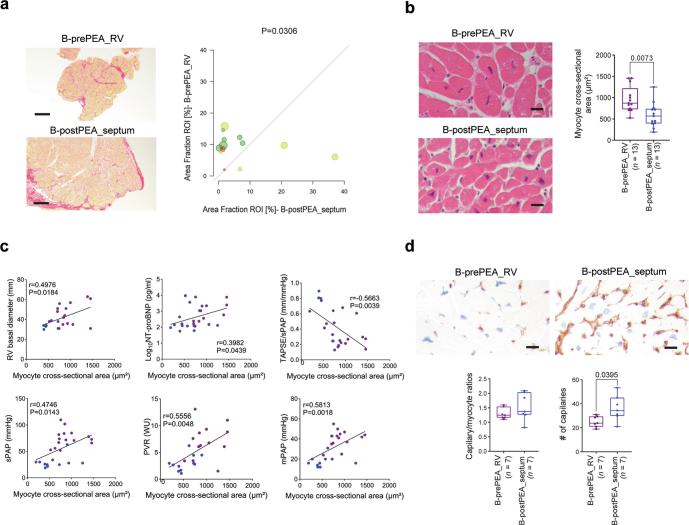
Hypertrophy and fibrosis in the confirmatory cohort of patients with CTEPH at prePEA_RV and postPEA_septum. **a**, Representative images and corresponding quantification of Sirius red-stained tissue sections of RV (B-prePEA_RV, *n* = 13 patients with a total of 47 fibrosis tissue sections) and septum (B-postPEA_septum, *n* = 13 patients with a total of 13 fibrosis tissue sections) in paired patient samples of the confirmatory cohort of patients with CTEPH. The area fractions were compared using a paired-sample t-test on the log fractions, using the total tissue area per patient as weights. Scale bar, 100 µm. **b**, Representative images and corresponding quantification of the cross-sectional view of the myocytes on H&E staining of RV (B-prePEA_RV, *n* = 13) and septum (B-postPEA_septum, *n* = 13) in paired patient samples from the confirmatory cohort of patients with CTEPH are displayed. Data are presented as mean ± SEM and *P* value was calculated by two-tailed paired t-test. Scale bar, 20 µm. **c**, Pearson correlation coefficient (r), and its associated two-tailed *P* value was performed between myocyte cross-sectional area and various clinical parameters. **d**, In the confirmatory cohort of CTEPH patients, paired tissue samples from the RV (B-prePEA_RV, *n* = 7) and septum (B-postPEA_septum, *n* = 7) were examined. Representative images and quantification of capillary/myocyte ratios, as well as the number of capillaries, are presented for both regions. Data are presented as mean ± SEM and *P* value was calculated by two-tailed paired t-test. Scale bar: 20 µm. Boxes show the interquartile range (IQR, 25^th^ to 75^th^ percentile), and the central bands indicate the median. The whiskers extend to 1.5 times the IQR above and below the box.

As RNA-seq data revealed transcriptomic differences in the septum of patients with CTEPH that change after PEA, we assessed whether these are reflected in histological remodeling. Hypertrophy, fibrosis and vascularization were analyzed in B-prePEA_RV samples (*n* = 13) and septal biopsies 12 months post-PEA (*n* = 13) (Supplementary Table 5). Fibrosis was significantly reduced post-PEA (Extended Data Fig. 8a), and the myocyte cross-sectional area was decreased in paired post-PEA septum samples (Extended Data Fig. 8b). This area significantly correlated with RV basal diameter, NT-proBNP, TAPSE/sPAP, sPAP, PVR and mPAP (Extended Data Fig. 8c). In addition, capillary density relative to myocardial volume was significantly higher in post-PEA septum samples compared with pre-PEA RV biopsies (Extended Data Fig. 8d and Supplementary Table 6), supporting reverse structural remodeling after PEA.

### *SERPINE1*, *IL7R* and *ANKRD1* link to RV recovery and PEA response

**Fig. 6:**
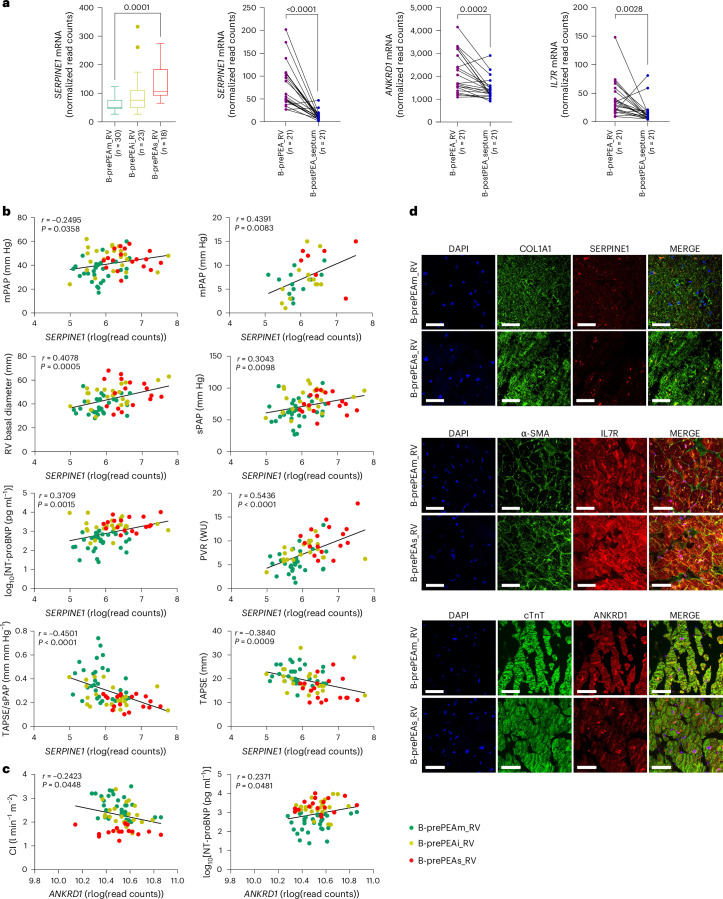
Expression levels of *SERPINE1*, *IL7R* and *ANKRD1* in the right ventricle and septum, and their correlation with the clinical parameters of the confirmatory cohort of patients with CTEPH. **a**, The mRNA expression levels (normalized read counts) of *SERPINE1* in CTEPH right ventricle at prePEA (B-prePEAm_RV, *n* = 30; B-prePEAi_RV, *n* = 23; B-prePEAs_RV, *n* = 18) and the mRNA expression levels of *SERPINE1*, *ANKRD1* and *IL7R* before PEA (B-prePEA_RV, *n* = 21) and after PEA (B-postPEA_septum, *n* = 21) in the confirmatory cohort of patients with CTEPH are shown. Data are presented as mean ± SEM, and *P* values were calculated using one-way ANOVA followed by Tukey’s multiple-comparison tests for expression of *SERPINE1* at prePEA and using a Wilcoxon test for the prePEA versus postPEA comparison. The boxes show the IQR (25th to 75th percentile), and the central bands indicate the median. The whiskers extend to 1.5 times the IQR above and below the box. **b**,**c**, The correlation between rlog(read counts) of *SERPINE1* (**b**) and *ANKRD1* (**c**), with clinical parameters of the patients before PEA (B-prePEAm_RV, B-prePEAi_RV and B-prePEAs_RV) is shown. Pearson’s correlation coefficient (*r*) and its associated two-tailed *P* value are shown in each graph. **d**, Representative immunofluorescent images of the right ventricle from the confirmatory cohort of patients with CTEPH before PEA are shown. The RV tissues from B-prePEAm_RV and B-prePEAs_RV patients underwent dual staining with SERPINE1 and COL1A1 (B-prePEAm_RV, *n* = 4, and B-prePEAs_RV, *n* = 3), IL7R and α-SMA (B-prePEAm_RV, *n* = 4, and B-prePEAs_RV, *n* = 3) and ANKRD1 and cTnT (B-prePEAm_RV, *n* = 4, and B-prePEAs_RV, *n* = 4) antibodies. Scale bars, 50 µm. Source data

Three genes—serpin family E member 1 (*SERPINE1*), interleukin 7 receptor (*IL7R*) and ankyrin repeat domain 1 (*ANKRD1*)—were selected to investigate whether their expression is altered post-PEA and how this regulation correlates with clinical parameters. *SERPINE1* is one of the genes that play an important role in the top-regulated ECM-related terms and pathways. *SERPINE1* is associated with the external encapsulation structure, extracellular matrix, collagen-containing extracellular matrix, matrisome and tube morphogenesis. *SERPINE1* expression was higher in the severe-risk group than in the moderate-risk group and correlated significantly with the clinical parameters of patients with CTEPH before PEA (Fig. 6a,b). *ANKRD1* is mainly associated with the major terms and pathways related to cardiac muscle, including actin filament-based processes and actin cytoskeleton organization, as well as muscle and heart development, particularly in comparisons between B-prePEA_septum and both Control_septum and B-prePEA_RV. The expression of *ANKRD1* was significantly correlated with the CI and NT-proBNP (Fig. 6a,c). The *IL7R* gene plays a crucial role in key signaling pathways, including the PI3K-Akt and PI3K-Akt-mTOR pathways, which are essential for focal adhesion and cell morphogenesis. Gene expression of *ANKRD1*, *SERPINE1* and *IL7R* was found to be significantly lower expressed in B-postPEA_septum samples than in B-prePEA_RV samples (Fig. 6a), with the expression changes being correlated with the hemodynamic changes induced by PEA in these patients (Supplementary Fig. 10a–c).

### *SERPINE1*, *IL7R* and *ANKRD1* respond to RV pressure overload

To identify the cell types expressing *SERPINE1*, *IL7R* and *ANKRD1*, the Human Cardiac Cell Atlas (v2) was analyzed^24^. Uniform manifold approximation and projection showed the distribution of these genes across 704,296 individual cells spanning 12 cardiac cell types (Supplementary Fig. 11a). In addition, human heart atlas data using Visium technology mapped the expression of these genes in three normal RV tissues (>55 years, 5,039 cells) and four normal septal tissues (>45 years, 8,643 cells) (Supplementary Fig. 11b,c)^25^. Correlation matrices of *SERPINE1*, *IL7R* and *ANKRD1* with virtual reference transcripts across myocardial cell types were generated using Human Protein Atlas data (Supplementary Fig. 12a–c). Expression of these genes and cell-type markers in cardiac single-cell clusters, along with interaction networks from the IntAct protein–protein interaction database, are shown in Supplementary Fig. 13a,b (ref. ^26^).

Across datasets, *SERPINE1* was mainly expressed in fibroblasts, ventricular cardiomyocytes, smooth muscle cells (cluster 3) and endothelial cells (cluster 6). *IL7R* appeared in lymphoid and myeloid cells, smooth muscle cells, endothelial cells and cardiomyocytes (cluster 1). *ANKRD1* was predominantly expressed in ventricular and atrial cardiomyocytes, and in some myeloid cells (Supplementary Figs. 11–13).

To confirm localization and upregulation, immunofluorescence staining was performed on RV tissue from patients with CTEPH in the B-prePEAm_RV and B-prePEAs_RV subgroups. On the basis of atlas data, RV samples were double stained for SERPINE1 and collagen type I alpha 1 chain (COL1A1), IL7R and alpha-smooth muscle actin (αSMA), ANKRD1 and cardiac troponin T (cTNT). Increased staining intensity and co-localization of SERPINE1 in fibroblasts, IL7R in smooth muscle cells and ANKRD1 in cardiomyocytes were observed in B-prePEAs_RV compared with B-prePEAm_RV (Fig. 6d).

To investigate what induces *SERPINE1*, *IL7R* and *ANKRD1*, primary cardiac cells were stimulated with TGF-β1, tumor necrosis factor (TNF) or hypoxia—factors relevant to compensated and decompensated RV states^7^ (Supplementary Figs. 14 and 15). In human cardiac fibroblasts (HCFs), *SERPINE1* and *ANKRD1* mRNA were significantly upregulated by TGF-β1 and TNF, while *IL7R* increased with TNF (Supplementary Fig. 14a). In human cardiac microvascular endothelial cells (HCMECs), *IL7R* was significantly increased by TNF, and all three genes were more highly expressed with TNF versus serum stimulation (Supplementary Fig. 14b). Under hypoxia, only *ANKRD1* showed increased expression in HCMECs after 24 h and 48 h compared with normoxia (Supplementary Fig. 15a,b).

**Extended Data Fig. 9:**
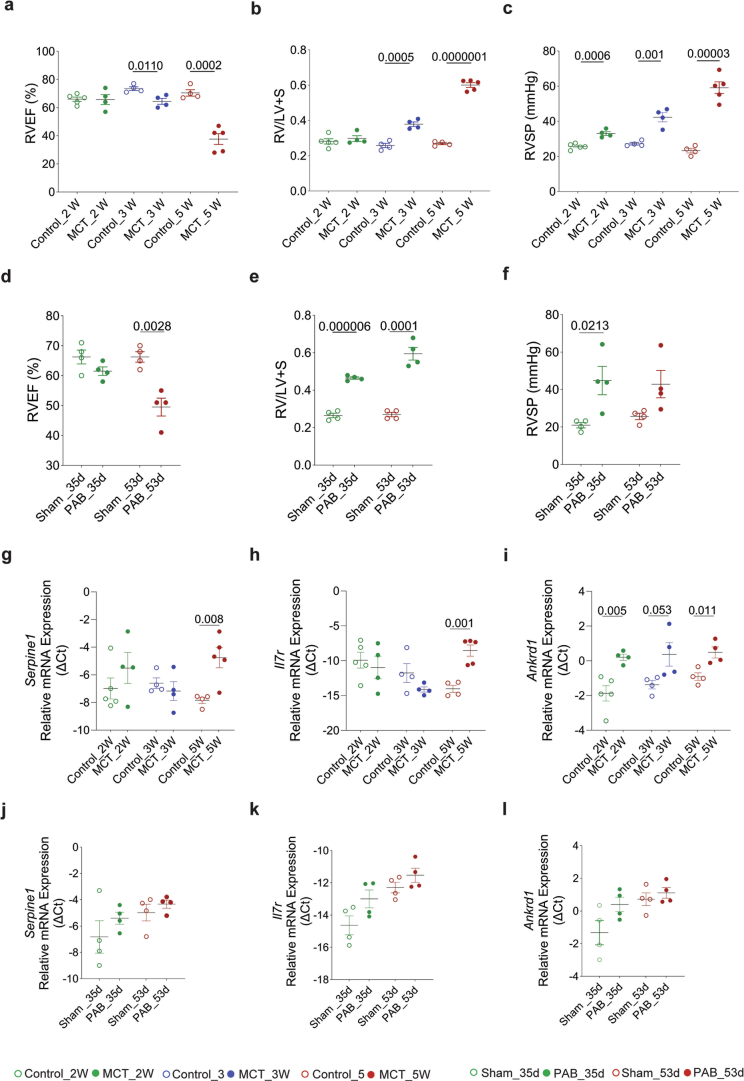
Assessment of hemodynamics parameters and RV function in MCT-induced PH and PAB rat models and *Serpine1*, *Il7r*, and A*nkrd1* gene expression validation in corresponding RV tissues. **a**, RV ejection fraction (RVEF), **b**, RV hypertrophy (the ratio of RV to LV+ septum), and **c**, RV systolic pressure (RVSP) in MCT-induced PH rats are presented for 2 W (weeks) (Control_2W, *n* = 5, and MCT _2W, *n* = 4), for 3 W rats (Control_3W, *n* = 4 and MCT _3W, *n* = 4), and 5 W rats (Control_5W, *n* = 4 and MCT_5W, *n* = 5). **d**, RVEF, **e**, RV/LV + S, and **f**, RVSP in PAB rat models are shown for two time points: 35 d (days) (Sham_35d, *n* = 4 and PAB _35d, *n* = 4), and for 53 d (Sham_53d, *n* = 4 and PAB _53d, *n* = 4). The relative mRNA expression levels of *Serpine1* (**g**), *Il7r* (**h**), and *Ankrd1*(**i**) in the RV of rats suffering from MCT-induced PH are shown. Additionally, the relative mRNA expression of *Serpine1*, *Il7r*, and *Ankrd1* in the RV of PAB rats are presented in panels **j**, **k**, and **l**, respectively. The mRNA levels were measured by qPCR, and *Gapdh* was used as an endogenous control. Data are presented as mean ± SEM and statistical significance was assessed by unpaired two-tailed t-test. (**g**, **h**, **i**): *n* (Control_2W) = 5, *n* (MCT_2W) = 4, *n* (Control_3W) = 4, *n* (MCT_3W) = 4, *n* (Control_5 W) = 4, *n* (MCT_5W-g, h) = 5, and *n* (MCT-5W-i) = 4. (**j**, **k**, and **l**): *n* (Sham_35d) = 4, *n* (PAB_35d) = 4, *n* (Sham_53d) = 4, *n* (PAB_53d) = 4.

To validate these findings in vivo, SERPINE1, IL7R and ANKRD1 expression was examined in two RV dysfunction models: monocrotaline (MCT) and PAB. Hemodynamic and echocardiographic measurements confirmed progressive RV dysfunction at 2, 3 and 5 weeks post-MCT treatment (Extended Data Fig. 9a–c) and at 35 days and 53 days post-PAB (Extended Data Fig. 9d–f). In MCT rats, *Serpine1*, *Il7r* and *Ankrd1* mRNA was significantly elevated at week 5 (Extended Data Fig. 9g–i). In PAB rats, *Serpine1*, *Il7r* and *Ankrd1* were increased (not significantly) (Extended Data Fig. 9j–l). *Serpine1* expression correlated with right ventricular ejection fraction (RVEF) and the ratio of RV mass to the combined LV and septal mass (RV/LV + S) in both models (Supplementary Fig. 16a). *Il7r* expression correlated with RVEF in MCT rats and right ventricular systolic pressure (RVSP) in PAB models (Supplementary Fig. 16b). *Ankrd1* expression correlated with RVSP, RVEF and RV/LV + S in both models (Supplementary Fig. 16c). Together, these findings suggest that *Serpine1*, *Il7r* and *Ankrd1* are key genes in RV remodeling. The downregulation of *Ankrd1* in de-banding mouse models further supports its regulation by pressure unloading.

### ANKRD1, SERPINE1 and IL7R regulate cardiac remodeling in vitro

**Fig. 7:**
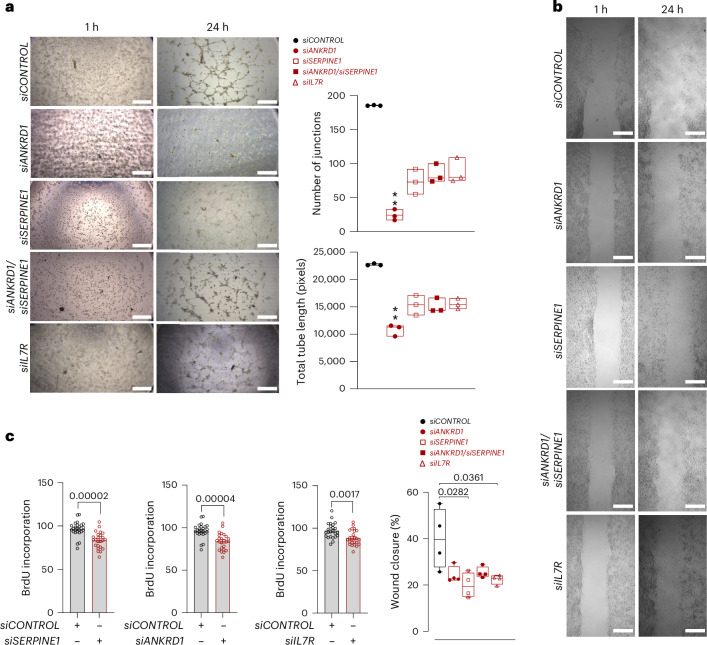
Inhibition of *SERPINE1*, *IL7R* and *ANKRD1* in HCFs and HCMECs. **a**, HCMECs were transfected with siRNA for 48 h, followed by a 24 h tube formation assay. The representative images were taken at 1 h and 24 h. The number of junctions and total tube length were analyzed and quantified using ImageJ software. Data are presented as mean ± SEM. (*n* = 3 biological replicates; Kruskal–Wallis test, **P = 0.0021 compared with scrambled siRNA). Scale bars, 1 mm. The boxes show the IQR (25th to 75th percentile), and the central bands indicate the median. The whiskers extend to 1.5 times the IQR above and below the box. **b**, HCFs were transfected with siRNA for 48 h, followed by a 24 h tube migration assay. Representative images were taken at 1 h and 24 h, and the percentage of wound closure was analyzed and quantified using ImageJ software with the wound healing analyzer plugin. A scrambled siRNA pool was used as a negative control. The data are presented as mean ± SEM. (*n* = 3 biological replicates with only one having one technical replicate; two-tailed unpaired *t*-test). Scale bars, 1 mm. The boxes show the IQR (25th to 75th percentile), and the central bands indicate the median. The whiskers extend to 1.5 times the IQR above and below the box. **c**, HCFs were transfected with siRNA for 48 h, and the proliferation was measured using BrdU incorporation assay. Data are presented as mean ± SEM. (*n* = 4 biological replicates and 6 technical replicates; two-tailed unpaired *t*-test). Black empty circles represent cells transfected with siCONTROL, and red empty circles represent cells transfected with target siRNA.

In vitro studies were performed on HCFs and HCMECs using transient transfection of small interfering RNA (siRNA) SMARTpool to investigate the functional role of ANKRD1, SERPINE1 and IL7R in modulating migration, tube formation and cell proliferation. As shown in Supplementary Fig. 17a,b, siRNAs targeting *ANKRD1*, *SERPINE1* and *IL7R* lead to a significant knockdown of the expression of the target genes in HCFs and HCMECs. As *SERPINE1* and *ANKRD1* are the major targets of SMAD/YAP/TAZ target genes^27^, the double knockdown of *ANKRD1* and *SERPINE1* suggests that *SERPINE1* and *ANKRD1* have extensive cross-talk in cardiac cells, especially with the greater effect of double knockout of both *SERPINE1* and *ANKRD1* (Supplementary Fig. 18a,b). The number of junctions, total tube length, total mesh area and total segment length were significantly reduced in cells transfected with *siANKRD1* compared with *siCONTROL* in HCMECs (Fig. 7a and Supplementary Fig. 19a). Notably, the effect on migration, that is, the percentage of wound closure, was significantly reduced in *siSERPINE1* and *siIL7R* compared with that in *siCONTROL* (Fig. 7b). In addition, single knockdown of *IL7R*, *ANKRD1* and *SERPINE1* and double knockdown of *ANKRD1* and *SERPINE1* significantly reduced the proliferation of HCFs and HCMECs (Fig. 7c and Supplementary Fig. 19b,c).

## Discussion

**Extended Data Fig. 10:**
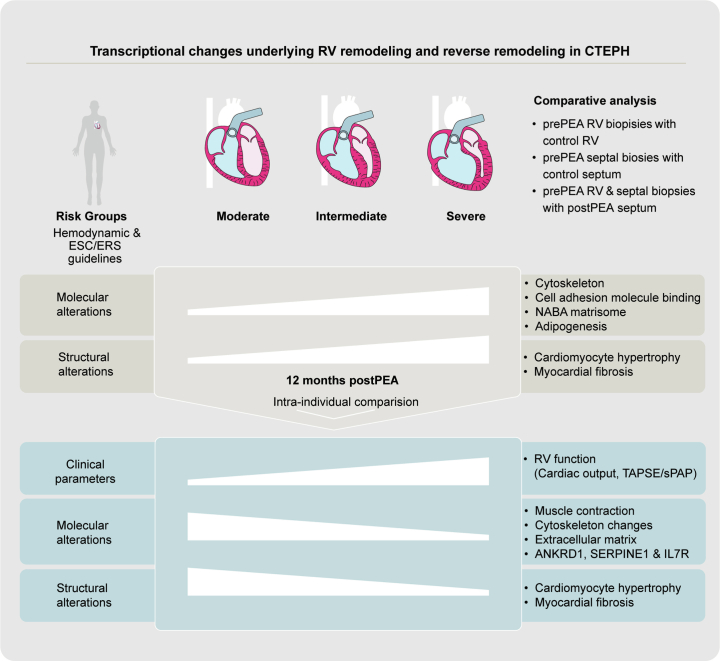
Schematic representation of the major findings. RV, right ventricular; CTEPH, chronic thromboembolic pulmonary hypertension; PEA, pulmonary endarterectomy; ESC/ERS, European Society of Cardiology/European Respiratory Society; TAPSE, tricuspid annular plane systolic excursion; sPAP, systolic pulmonary artery pressure; ANKRD1, Ankyrin Repeat Domain 1; SERPINE1, Serpin Family E Member 1; IL7R, Interleukin 7 Receptor. Created by Adobe Illustrator.

Characterization of RNA profiles from fresh biopsies of the RV free wall and septum of patients with CTEPH and assessment of the dynamics of these profiles in relation to disease severity and in response to PEA provided valuable information on the RV remodeling and reverse remodeling mechanism in CTEPH. The following are the most important results: first, similar to hemodynamics, RV imaging and biomarkers such as natriuretic peptides, RV free wall RNA profiles differ substantially between patients with CTEPH and controls and between moderate- and severe-CTEPH-risk groups. Second, the RV free wall transcriptomic profiles obtained in a smaller exploratory cohort were validated in a larger confirmatory cohort, providing a solid basis for future use of RNA profiling for in-depth characterization of RV function in patients with PH. Third, histological and molecular analyses of septal tissue show that the RV free wall remodeling is accompanied by extensive septal remodeling, with 40–45% overlap of transcriptomic changes. At both sites, myocyte cytoskeletal changes are accompanied by major matrisomal changes in intermediate- and severe-risk groups. Fourth, intraindividual comparison of histological and gene expression profiles (septal to septal comparison or free wall comparison to the overlapping septal transcriptome) before and after PEA shows far-reaching reversal of the myocyte cytoskeletal and matrisomal changes. Similarly, reversal of transcriptomic changes was partly found in an experimental PA banding and de-banding model. Fifth, various signaling molecules and pathways were identified (ANKRD1, IL7R, SERPINE1), correlating with disease severity (hemodynamics, RV function) and reverse remodeling, putatively offering for RV-focused preventive or therapeutic strategies (Extended Data Fig. 10).

The current PH guidelines recommend a multidimensional diagnostic approach for individual risk stratification. Calculating risk scores from the three-strata ESC and ERS risk stratification model using parameters focused on the right heart (CI, TAPSE/sPAP and natriuretic peptides) for the CTEPH cohorts allowed us to define patients at moderate, intermediate or severe risk^8^. Similar to other CTEPH cohorts, patients in our study had markedly impaired pulmonary hemodynamics associated with elevated natriuretic peptides^11,28^. In addition to the functional status of the right ventricle, patients also differed from controls in terms of transcriptional characteristics of the RV free wall, indicating molecular, cellular and structural changes underlying the development of functional RV phenotypes. Importantly, the transcriptomic profiles of the RV free wall obtained in a smaller exploratory cohort were validated in a larger confirmatory cohort, providing a solid foundation for the future use of RNA profiling for in-depth characterization of RV function in PH patients.

Comparison of the RNA profiles in the different risk classes in both the exploratory and confirmatory cohorts to normal RV control tissue revealed enrichment of various GO terms such as enzyme–receptor–protein signaling pathway, cytoskeleton, cell adhesion molecule binding, NABA MATRISOME ASSOCIATED and adipogenesis. These transcriptomic changes increased with risk class severity, and in particular, changes in highly regulated ECM-related genes were found to be correlated with indicators of disease severity such as RV basal diameter, mean right arterial pressure (mRAP), CI, PVR, mPAP, NT-proBNP, sPAP and TAPSE/sPAP. In companion with the increased fibrotic changes in the histologic sections of free RV wall biopsies, these findings are well in line with previous studies, documenting increased fibrosis in cardiac tissue originating from patients with idiopathic PAH or CTEPH^29^. This fibrotic remodeling observed in the CTEPH high-risk group might represent a maladaptive response, linked with increased diastolic stiffness, disrupted cardiomyocyte excitation–contraction coupling and impaired myocardial contraction coordination^30,31^.

Adipogenesis was consistently enriched in intermediate- and severe-risk groups in both cohorts. This aligns with findings that increased epicardial adipose tissue volume is associated with RV end-diastolic volume and NT-proBNP in PAH, and linked to reduced long-term event-free survival in patients with PAH^32^. Similarly, lipid accumulation and fatty acid metabolism alterations have been reported in the right ventricle of patients with PAH^33,34^. While the role of adipose tissue and lipotoxicity in maladaptive RV remodeling is not fully understood, their impact on diastolic function and pro-fibrotic signaling—such as TGF-β activation—should be considered^35^.

GO terms related to inflammation—such as regulation of inflammatory response, cytokine signaling and neutrophil degranulation—were enriched in CTEPH RV tissue compared with controls, increasing with disease severity. This indicates progressive inflammatory changes in the right ventricle. RV inflammation has previously been reported in various PH forms, including acute pulmonary embolism and HIV-associated, systemic-sclerosis-associated and idiopathic PAH^7,30,36^. Although not directly studied in CTEPH, RNA-seq data from decompensated PAH right ventricles showed increased inflammatory gene signatures, with elevated CD68^+^ macrophages and IL-1β levels, compared with controls^7,30^. These findings, together with our current data, suggest that inflammation is a key contributor to adverse RV remodeling under chronic pressure overload in CTEPH^36^.

In summary, a sequence of transcriptomic, cardiomyocyte-phenotypic and matrisomal changes appear to underlie the transition from an adaptive to a maladaptive response of the right ventricle under CTEPH conditions. For the selection of therapeutic targets specifically focusing on these RV sequence of events, it is of interest that only 10–40% of the RV transcriptomic changes overlap with transcriptomic changes reported to underlie left ventricular failure^20,22^. Whether these results can be extrapolated to PH groups other than CTEPH requires further integrated bioinformatic and clinical correlation analyses in non-CTEPH cohorts. Previous studies from our and other groups^6,7^ in RV-omics in PH suggest that despite some common regulatory pathways (for example, signaling cascades related to ECM, cell cycle, energy metabolism and inflammation) in different PH groups, distinct and uniquely enriched pathways (for example, epithelial–mesenchymal transition in right ventricles in PAH, increased oxidative stress in right ventricles in CTEPH) may exist, which require further in-depth investigation.

Although changes in septal thickness and motion, correlating with RV afterload, are well known for PH constellations including CTEPH^37,38^, there are only scarce data on detailed histological and molecular profiling of septal biopsies under these conditions. We observed septal histomorphological (cardiomyocyte hypertrophy and increased fibrosis) changes largely corresponding to those in the free RV wall, as well as enrichment of GO terms associated with cytoskeletal changes of the cardiomyocytes and the ECM. Interestingly, however, the transcriptomic profiles of the remodeled septum overlaid not fully, but around 40–45%, with the transcriptome of the remodeled RV free wall. Septal remodeling may thus have some specific features, possibly related to its origin of both first and second heart fields^39^, whereas the RV free wall originates from the second heart field, or to its positioning between the right and the left ventricle, receiving biomechanical forces from both sides.

The therapy of choice in patients with CTEPH is surgical PEA. For inoperable patients, an interventional balloon pulmonary angioplasty is recommended^9^. Both therapies improve pulmonary hemodynamics and allow right heart recovery, which is illustrated by invasive hemodynamic measurements, imaging findings and biomarker dynamics. Comparable to other cohorts, the patients with CTEPH in our study showed markedly improved pulmonary hemodynamics, decreased natriuretic peptides and an improved right heart function after therapy^28,40–47^. Our study, however, performs an intraindividual comparison of heart biopsies before and after PEA in these patients with CTEPH. This approach shows a reduction in hypertrophy and fibrosis and an improvement in vascularization relative to RV myocardial tissue volume in response to the afterload relief, correlating with improved clinical parameters (RV basal diameter, TAPSE/sPAP and PVR) 12 months after PEA. A comparison of RNA profiles between RV free wall and septal biopsies before and after PEA, focusing on overlapping transcriptomic changes between these two sites, revealed alterations of terms related to muscle contraction, cytoskeleton changes and ECM, corresponding to most of the pathways that were markedly altered in intermediate- and severe-risk patients with CTEPH, suggesting reverse remodeling of the RV septal molecular, cellular and structural changes in response to PEA. However, residual myocardial fibrosis in septal biopsies and the identification of GO terms associated with ECM changes in patients with CTEPH 12 months after PEA suggest still incomplete reversal of fibrosis. A comparison of these human reverse-remodeling gene expression datasets with the RV tissue gene arrays of the PA banding–de-banding mouse model^23^ unexpectedly shows only 10% overlap between the human and the mouse system. Imperfect mimicry of the CTEPH and PEA constellation by the banding and de-banding procedure, differences in observation periods and species specificities might explain these differences. However, reversal of the afterload-induced RV transcriptomic changes was also shown in the mouse model.

Three DEGs associated with reverse remodeling—*ANKRD1/CARP*, *SERPINE1* and *IL7R*—showed significant correlations between B-postPEA_septum and B-prePEA_RV expression levels and patient hemodynamics. Public databases (human cardiac cell atlas, heart atlas, protein atlas) and double immunofluorescence staining linked SERPINE1 to fibroblasts, IL7R to smooth muscle cells and ANKRD1 to cardiomyocytes. Experimental data suggest roles in cell proliferation, migration, wound healing and angiogenesis. These genes were upregulated by hypertrophic stimuli and pathways associated with decompensated right ventricles, such as TNF and TGF-β (ref. ^7^). ANKRD1 expression has been linked to reduced contractility and compliance in end-stage heart failure^48^ and modulates remodeling via the ERK/GATA4 pathway^49,50^. SERPINE1/PAI-1 may either repress fibrosis or promote ECM accumulation and scarring^51^. IL7R upregulation suggests inflammatory cell recruitment and chemokine expression in maladaptive right ventricles. Further studies are needed to clarify their roles in RV failure and recovery under CTEPH and PEA conditions.

Taken together, comprehensive transcriptomic profiles from RV and septal biopsies of living patients with CTEPH not only provide valuable information on the molecular mechanisms underlying RV and septal remodeling during increased afterload and its reversibility upon unloading, but also offer targets for therapeutic intervention.

## Limitations

In this study, RNA-seq analysis was performed in patients with CTEPH undergoing PEA. However, the small number of consecutively enrolled patients in the study cohort was a limitation that needed to be considered. However, the data were sufficient to show considerable differences in RNA profiles between moderate- and severe-risk patients with CTEPH and could be validated in a second confirmatory cohort. This study used a right-heart-focused risk stratification model adapted from the ESC/ERS guidelines for PH. This model has not yet been validated in a clinical outcome study. However, all recommended diagnostic aspects (hemodynamics, biomarkers, imaging) of the right heart examination were considered. Furthermore, as a human model of RV remodeling and reverse remodeling after PEA, CTEPH provides an ideal intra-observation opportunity compared with other datasets derived from transplanted hearts. Despite the different locations of biopsies between the RV free wall and septum, the timing of the biopsy removal and the effect of the surgical procedure, our data clearly show several overlaps between the RV wall and septum, emphasizing the relevance of the results.

Additional limitations of the study lie in the animal models used to validate the transcriptome results of a few genes (*SERPINE1*, *IL7R* and *ANKRD1*). We used RV tissues from the MCT and PAB models. Although we expect the same changes in the in vivo CTEPH model, the question of whether the molecular changes are mainly caused by pressure unloading remains to be validated in the in vivo CTEPH model. Although we have confirmed the regulation of some genes at the protein level, not all RNA changes can be translated into changes in protein expression, so a proteomic study of this valuable human cohort is warranted. As the in vitro experiments were performed on cardiac cells from the LV and the LV and right ventricle are developmentally distinct, additional experiments with cells isolated from the right ventricle are important.

## Methods

### Ethics in human studies

The study of CTEPH cohorts was approved by the ethics board of the Justus Liebig University of Giessen (AZ 44/14, 144/11, 145/11, 146/11, 199/15) and is in accordance with the declaration of Helsinki, and all the patients with CTEPH enrolled in the study gave written informed consent. For the healthy control participants, all experimental procedures were conducted in accordance with the ethical standards of the responsible institutional and national committee on human experimentation, as outlined in the Helsinki Declaration (1975). Written informed consent was obtained from all control participants involved in the study according to the protocol approved by the Local Ethics Committees of the National Institute of Cardiology, Warsaw, Poland (approval number: IK-NPIA-0021-14/1426/18).

### Ethics in animal studies

All the animal experiments were conducted in accordance with the National Institute of Health Guidelines on the Use of Laboratory Animals. The study protocols were approved by the University Animal Care Committee and the Federal Authorities for Animal Research of the Regierungspräsidium Giessen (GI 20/10 Nr G92/2017 RP Giessen), Hessen, Germany.

### Patient cohorts and clinical assessment

This study included 96 patients with CTEPH, who were treated by PEA at the Kerckhoff Heart and Thorax Centre between 2016 and 2020. The standard periprocedural management of such patients was recently published^40^. In brief, clinical examination, echocardiography, 12-lead electrocardiogram, laboratory tests, 6-min walk test, ventilation–perfusion scan, computed tomography angiography, right-heart catheterization (RHC) and pulmonary angiography were assessed for all patients before PEA (prePEA). The final diagnosis of CTEPH was made according to the guidelines in symptomatic patients after 3 months of effective anticoagulation, with an mPAP ≥ 25 mm Hg at rest and typical obstructive pulmonary vascular lesions on imaging diagnostics^52^. All patients were presented in an interdisciplinary CTEPH conference to define the therapeutic concept and approved feasibility for surgical PEA as the first-line therapy. Routinely, a standardized follow-up assessment of patients was performed 12 months after PEA (postPEA).

### PEA

The performance of PEA was conducted according to the standardized procedure^40^. In brief, a median sternotomy was followed by the connection of patients to the heart–lung machine for extracorporeal circulation, with subsequent cooling to a core temperature of 18 °C. PEA was performed in repetitive phases of deep hypothermic circulatory arrest to avoid collateral blood backflow from systemic arteries and to optimize visualization. The removal of scar tissue from the pulmonary vasculature was accomplished by creating an endarterectomy plane between the intima and media of the vessel wall, with the aim of achieving complete excision. Following the endarterectomy, patients were rewarmed and weaned from bypass at a body temperature of 36 °C. The standard postoperative care includes a stay at the intensive care unit for another 2 days, extubation on the second postoperative day and discharge after 12 days (ref. ^40^).

### RHC

As a component of the diagnostic protocol, patients underwent RHC at before PEA and 12 months after PEA. The RHC was routinely performed via the right internal jugular vein using a 6-F sheath and a standard Swan–Ganz catheter. The medication of the patients was not modified before or during RHC; in particular, no vasoactive agents were administered.

### Human RV and septum acquisition and classification of patients with CTEPH

This prospective study enrolled a total of 96 patients with CTEPH, divided into two distinct cohorts: an exploratory cohort and a confirmatory cohort. RV biopsies were obtained from the right ventricular free wall of the patients who underwent the PEA procedure (prePEA_RV) from two cohorts: the exploratory cohort (A-prePEA_RV, *n* = 14) and the confirmatory cohort (B-prePEA_RV, *n* = 88). The patients in each cohort were risk stratified based on their clinical parameters and ESC/ERS guidelines using a three-strata model into moderate-, intermediate- and severe-risk groups^8^. In addition, the study included 26 postPEA septum biopsies collected 12 months after PEA, and 21 were matched with prePEA counterparts. In addition, in the confirmatory cohort, in the subgroup of 3 patients, with the biopsies before PEA from the right ventricle (B-prePEA_RV, *n* = 3) and 12 months after PEA from the septum (B-postPEA_septum, *n* = 3), the biopsy was obtained from the septum before PEA (B-prePEA_septum, *n* = 3). The clinical parameters of all the patients with CTEPH are shown in Table 1 and Supplementary Tables 1–6. All the specimens were processed in a standardized manner by experienced staff, who were blinded to clinical data. Out of 88 RV biopsy samples, RNA-seq was performed on 71 samples and histological analysis on 43 samples, with 26 samples overlapping between the two datasets. The allocation to RNA-seq or histology was not based on any pre-selection, bias, or stratification criteria, but rather determined solely by the amount of tissue available from each biopsy.

Tissue samples from healthy control participants were collected from the same patient (*n* = 10) for both the right ventricle and septum. The tissue samples for control were collected in the Department of Heart Failure and Transplantology, National Institute of Cardiology, Warsaw, Poland. Healthy human hearts were obtained from organ donor patients (control, *n* = 10) whose hearts were not used for transplantation owing to technical reasons (for example, donor and recipient incompatibility). The donors did not have any relevant previous cardiological history or any abnormalities in electrocardiography and echocardiography (LV dimensions and contractility within normal ranges). The tissue samples from the ventricular free wall were taken (avoiding scarred, fibrotic or adipose tissue, endocardium, epicardium or coronary vessels).

### Total RNA extraction from human heart tissue

We used two distinct RNA extraction methods to isolate the total RNA of human heart tissue of patients with CTEPH. In the bead beating homogenization method, tissue homogenization was performed using a BeadBug 3 homogenizer (Biozym). Subsequently, RNA isolation was performed using a miRNeasy Micro Kit (Qiagen, 217084) according to the manufacturer’s instructions. RNA isolation was performed using this method on the RV tissue of the exploratory cohort of patients with CTEPH (A-prePEA_RV, *n* = 14).

The dry cryopulverization method was performed to isolate the RNA from the right ventricle and septum tissue from the confirmatory cohort of patients with CTEPH including B-prePEA_RV (*n* = 71), and septum tissue of B-postPEA_septum (*n* = 21) and B-prePEA_septum (*n* = 3). The biopsy samples were transferred to pre-chilled TT05 culture tubes and processed using a CP02 cryoPREP Automated Dry Pulverizer 110 V (Covaris). The remaining RNA isolation steps were performed using the miRNeasy Micro Kit (Qiagen, 217084) according to the manufacturer’s protocol. Total RNA was extracted from the right ventricle and septum of the healthy control participants, using the RNeasy PowerLyzer Tissue & Cells Kit (Qiagen) according to the manufacturer’s instructions outlined in the RNeasy handbook. To prevent genomic DNA contamination, isolated RNA was treated with on-column DNase digestion (DNase-Free DNase Set, Qiagen).

### Library preparation and RNA sequencing data analysis

RNA and library preparation integrity were verified with LabChip Gx Touch 24 (Perkin Elmer). For library preparation, approximately 100 ng to 1 µg of total RNA was used as input for the SMARTer Stranded Total RNA Sample Prep Kit - HI Mammalian (Takara Bio). Sequencing was performed on a NextSeq 2000 (Illumina) using P2 flow cell with v3 chemistry with 1 × 72 bp single-end setup or NextSeq500 (Illumina) using v2 chemistry with 1 × 75 bp single-end setup. The resulting raw reads were assessed for quality, adapter content and duplication rates with FastQC^53^. Trimmomatic version 0.39 was applied to trim reads after a quality drop below a mean of Q15 and Q18 in a window of five nucleotides. Afterward, only filtered reads longer than 15 nucleotides were kept^54^. Only reads above 30 nucleotides were cleared for further analyses. The reads were aligned versus Ensembl human genome version hg38 using Ensembl release 101 for the control cohort and release 104 for the CTEPH cohorts. The alignment was performed with STAR, using version 2.7.9a for the control cohort and version 2.7.10a for the CTEPH cohort. The parameter ‘--outFilterMismatchNoverLmax 0.1’ was set to increase the maximum ratio of mismatches to mapped length to 10% (ref. ^55^). Aligned reads were filtered to remove duplicates with Picard 2.27.1 (Picard Toolkit, 2019, Broad Institute, GitHub repository, https://broadinstitute.github.io/picard/; Broad Institute; RRID: SCR_006525), multi-mapping, ribosomal or mitochondrial reads. The gene counts were established with the featureCounts 2.0.2 tool from the Subread package^56^. Only reads mapped to the exon and aggregated per gene were included for analysis. The reads with overlapping multiple genes were excluded. DEGs were identified using DESeq2 version 1.30.0 applied to the control cohort and 1.30.1 applied to the CTEPH cohort^57^. Based on the raw count matrix, the contrasts were created with DESeq2.

### Downstream analysis of RNA-seq data

Significantly differentially expressed genes were identified using a threshold of mean read count ≥5 and a maximum Benjamini–Hochberg corrected *P* value of ≤0.05, and |log_2_(FC)| ≥ 0.585. The annotations were enriched with UniProt data based on Ensemble gene identifiers (activities at the Universal Protein Resource (UniProt)). All the downstream analysis was performed based on the normalized gene count matrix. A global clustering heatmap of the samples was generated based on the Euclidean distance of regularized log-transformed gene counts. PCA as a dimension reduction analysis was performed on regularized logarithm-transformed counts using R package^58^. Volcano plots were generated to visualize the DEGs based on DESeq2 normalized counts. For further analysis, R (version 4.2.0) and RStudio (version 2023.03.0, Build 386) were used in conjunction with several specialized R packages including DESeq2 (version 1.38.3) for the differential gene expression analysis, sva (version 3.46.0), ComBat_seq for surrogate variable analysis and batchcorrection^59^ and Complexheatmap (version 2.14.0) for Heatmaps^60,61^.

Pathway enrichment analyses were conducted using Metascape (www.metascape.org). Pathway and process enrichment analysis was performed with the default settings, using KEGG Pathway, GO Biological Processes, GO Cellular Components, GO Molecular Functions, Reactome Gene Sets, Canonical Pathways, CORUM, WikiPathways, PANTHER Pathway and all genes in the genome as the enrichment background. Terms with a *P* value <0.01, a minimum count of 3 and an enrichment factor >1.5 were collected and clustered based on their membership similarities. *P* values were calculated based on the cumulative hypergeometric distribution, and *q* values were computed using the Benjamini–Hochberg procedure to account for multiple testing. Kappa scores were used as the similarity metric when performing hierarchical clustering on the enriched terms, and sub-trees with a similarity of >0.3 were considered a cluster. The most statistically significant term within a cluster was selected to exemplify the cluster. For multiple gene lists, these were merged into a single list named ‘_FINAL’. If the terms were enriched in several individual gene lists and/or the _FINAL gene list, the best *P* value was selected as the final *P* value^62^.

### Statistical analysis

Several tests were used for statistical analysis using GraphPad Prism version 9.1.0 (GraphPad Software). For normally distributed variables, comparison between two groups was performed using unpaired *t*-tests, while paired *t*-tests were used where applicable. Non-normally distributed variables are presented as median (interquartile range (IQR)). The comparison between the two groups was performed using the Mann–Whitney *U*-test, while the Wilcoxon matched-pairs signed-rank test was used for the paired data. For the comparisons involving more than two groups, one-way analysis of variance (ANOVA) was used for the normally distributed variables. For the non-normally distributed variables, the Kruskal–Wallis test was used. In addition, categorical variables are shown as counts and percentages and Fisher’s exact test was performed to compare the categorical variables. Pearson’s correlation analysis was used to show the relationship between the regularized logarithm transformation (rlog) read counts of the selected genes and the clinical parameters of the patients.

### Histopathological, immunohistochemical and immunofluorescence analyses

For investigation of fibrosis and hypertrophy in patients with CTEPH, RV and septum tissue were sectioned into 5-µm-thick slices from embedded paraffin tissue. To assess fibrosis, Sirius red staining was performed, while hematoxylin–eosin (H&E) staining was used to evaluate hypertrophy. To quantify the fibrosis in the right ventricle and septum of patients with CTEPH, the cellSens Standard software version 4.3 (Evident Scientific) was used. For the analysis of fibrotic area fractions, a variable number of sections per patient were analyzed to give a tissue area and the corresponding areas of the region of interest (ROI). Areas were summed up per patient, and the area fraction of the ROI per patient was calculated by dividing the summed areas (ROI area/tissue area). Differences in the area fractions between the different risk groups were analyzed in R 4.1.1 (ref. ^63^) using a weighted generalized linear model of the quasi-binomial family, using the total tissue areas per patient as weights. The area fractions in the right ventricle and septum were compared using a paired-sample *t*-test on the log fractions, using the total tissue area per patient as weights.

The cross-sectional area of cardiomyocytes was morphometrically assessed using Olympus CellSens Entry 2.3 software by selecting the best-preserved cardiomyocytes with centrally located nuclei, ensuring accurate and reliable measurements.

For the assessment of vascularization, the heart tissue sections were stained according to a standardized protocol (Bond Max staining protocol) using the monoclonal CD34 antibody Novocastra (Liquid Mouse Monoclonal Antibody Endothelial Cell Marker, number NCL-L-END, Leica Biosystems Newcastle). The BOND Polymer Refine Detection system (number DS9800, Leica Biosystems Newcastle) was applied for visualization. The tissue sections were also counterstained with hematoxylin, resulting in endothelial cells appearing brown and cell nuclei appearing blue to violet. Quantification was performed using Olympus CellSens Entry 2.3 software.

For immunofluorescence analysis, RV tissue from subgroups of patients with CTEPH at prePEA including B-prePEAm_RV and B-prePEAs_RV were embedded in OCT and sectioned into 8-µm slices. Tissue sections were washed with PBS before undergoing a 10-min incubation with a permeabilization buffer containing 0.5% Triton X-100 (Carl Roth) at room temperature. Following an additional set of PBS washes, a blocking buffer, PBS supplemented with 5% bovine serum albumin (Carl Roth), was applied and allowed to incubate for 1 h at room temperature. Subsequently, the slides were incubated with two primary antibodies including PAI1 monoclonal antibody (SERPINE1, 1:200, number MA1-40224, Thermo Fisher) and COL1A1 polyclonal antibody (1:500, number PA5-29569, Thermo Fisher), CD127 polyclonal antibody (IL7R, 1:200, number PA5-102399, Thermo Fisher) and alpha-smooth muscle actin antibody (αSMA, 1:2,000, number NB300-978, Novus), CARP polyclonal antibody (ANKRD1, 1:400, number PA5-101170, Thermo Fisher) and cardiac troponin T monoclonal antibody (13-11) (cTnT, 1:1,000, number MA5-12960, Thermo Fisher) diluted in PBS for co-staining, and incubated overnight at 4 °C. The following day, tissue sections were washed with PBS, and slides were incubated with corresponding secondary antibodies (anti-mouse 594, 1:500, number A21203, Thermo Fisher; anti-rabbit 488, 1:500, number A11008, Thermo Fisher; anti-rabbit 594, 1:500, number A32740, Thermo Fisher; anti-goat 488, 1:500, number A21467, Thermo Fisher; anti-mouse 488, 1:1,000, number A11029, Thermo Fisher) for 1 h at room temperature. Afterward, sections were stained with DAPI (Thermo Scientific) at a dilution of 1:1,000 in PBS, followed by a 10-min incubation and a final set of PBS washes. Ultimately, slides were mounted and covered with coverslips, then stored at 4 °C, or prepared for immediate fluorescent image acquisition with an LSM 710 confocal microscope.

### PAB and MCT rat modes

In animal studies, Sprague Dawley male rats (*Rattus norvegicus*) aged 8–10 weeks (PAB group) and 10–12 weeks (MCT group) were obtained from Charles River and subjected to the treatment. PAB rats were anesthetized with a subcutaneous injection of analgesic buprenorphine hydrochloride (Temgesic, 0.1 mg kg^−1^, Sigma-Aldrich) and maintained with 3–4% isoflurane mixed with oxygen via inhalation. Following intubation, the rats were connected to a ventilator (MiniVent type 845 Hugo Sachs Elektronik), and the respiratory rate and the stork volume were adjusted according to animal weight. The left thoracic wall of the rats was depilated and disinfected with Braunoderm (Braun). A small skin incision was made at the axillary level followed by careful separation of the thoracic muscles to open the chest cavity through a small incision in the second intercostal space. This exposed the pulmonary artery, aorta and left atrium. The pulmonary artery was then carefully separated from the aorta and left atrium, and an L-shaped blunted needle was placed around it, secured tightly against the needle. A titanium ligating clip (Hemoclip, Edward Weck) was placed around the pulmonary artery to produce 65–70% constriction. The chest cavity was then closed, and the muscles were repositioned to their original place. In the sham groups, the pulmonary artery was dissected using an L-shaped needle, but no ligating clip was applied, and the chest cavity was immediately closed.

MCT rats were injected with either a single subcutaneous MCT dose (60 mg kg^−1^ body weight) or an equal volume of saline for the control group.

The hemodynamics measurements were performed after 2, 3 and 5 weeks of MCT injection in rats and, for the PAB rats, on day 35 and 53 after the PAB and sham operation. The hemodynamic parameters include cardiac contrast-enhanced microscopic computed tomography (µCT), RHC and RV hypertrophy measurements as described^64^.

### Treatment of human cardiac cells

The HCFs were purchased from ScienCell Research Laboratories (catalog number 6300) and cultured on poly-L-lysine-coated dishes in fibroblast medium as recommended by the supplier. HCMECs were purchased from PromoCell (catalog number C-12285) and maintained in Endothelial Cell Basal Medium MV2, supplemented with SupplementPack Endothelial Cell GM MV2. The cells were cultured at 37 °C in a humidified chamber with 5% CO_2_. HCF cells and HCMECs were treated with different PH stimuli, including TGFβ1 (10 ng; Peprotech) and TNF (10 ng; Peprotech), for 48 h. For hypoxia (Hox) treatment, HCF cells and HCMECs were maintained in a humidified hypoxia chamber with 5% CO_2_ and 1% O_2_ at 37 °C for 6, 24 and 48 h. By contrast, the normoxia (Nox) condition was used as a control, and the cells were maintained with the same medium in a cell culture incubator with 5% CO_2_ and 21% O_2_ at 37 °C.

### RNA interference

Transfection of HCF cells and HCMECs was performed using isoform-specific SMARTpool siRNAs against ANKRD1, SERPINE1 and IL7R with Lipofectamine 3000 (Invitrogen) as the transfection reagent, according to the manufacturer’s instructions. siRNAs were purchased from Dharmacon, Horizon Discovery, and a non-targeting siRNA was used as a negative control (Supplementary Table 7).

### Bromodeoxyuridine cell proliferation assay

Measurements of cell proliferation in HCFs and HCMECs were conducted using a Cell Proliferation ELISA, bromodeoxyuridine (BrdU) (colorimetric) kit (Roche), following the manufacturer’s protocol. HCF cells and HCMECs were seeded in 48-well plates at 48 h after siRNA transfection; cells were then serum starved for 24 h, followed by a 2-h incubation with BrdU labeling solution. Cells were washed with PBS and fixed with FixDenat solution, followed by incubation with tetramethylbenzidine (TMB) solution until color development. Absorbance was measured at a wavelength of 370 nm with reference at 492 nm using a TECAN Infinite M200 PRO microplate reader.

### Wound healing assay

The HCFs were transiently transfected with siRNA for 48 h, then a cell suspension (5 × 10^5^ cells ml^−1^) from siRNA-transfected cells was added to each well of the Culture-Inserts 2 Well (Ibidi). The cells were incubated at 37 °C and 5% CO_2_ for 24 h. The images were captured at 1 h and 24 h post-insert removal using a ×4 objective lens on a Leica DM 6000B microscope. The wound area was oriented vertically during image acquisition. The microscopic pictures were analyzed using ImageJ software with the wound healing plugin.

### Endothelial cell tubulogenesis assay

HCMECs were transfected with siRNA for 48 h. Before cell seeding, the Matrigel Basement Membrane Matrix Growth Factor Reduced (Corning) was applied to each well. The cell suspension (2 × 10^5^ cells ml^−1^) from siRNA-transfected cells was added to each well of µ-slide. Images were collected at different time points (1 h and 24 h) using a ×4 objective lens on a Leica DM 6000B microscope. The microscopic pictures were analyzed using ImageJ software with the angiogenesis analyzer plugin.

### RNA isolation from the cardiac cells and rat RV tissue, and RT-qPCR

Total RNA was extracted from cardiac cells (HCFs and HCMECs) and right ventricles of rat animal models (PAB and MCT) using the RNeasy Mini Kit (Qiagen) following the manufacturer’s instructions. The cDNA synthesis was performed on equal amounts of RNA using the High-Capacity cDNA Reverse Transcription Kit with RNase Inhibitor (Thermo Fisher Scientific) according to the manufacturer’s instructions. RT-qPCR was performed using iTaq Universal SYBR Green Supermix (BioRad) in the CFX96 Real-Time PCR detection system (BioRad). Gene expression was determined using the ∆Ct method as previously described^65^. Hypoxanthine phosphoribosyltransferase 1 (HPRT1) and glyceraldehyde 3-phosphate dehydrogenase (GAPDH) were used as a reference gene (endogenous control). Intron-spanning human and rat-specific primers were designed using sequence information from the NCBI database and were purchased from Metabion International AG, Sigma-Aldrich and Merck KGaA, respectively (Supplementary Tables 8 and 9).

### Reporting summary

Further information on research design is available in the Nature Portfolio Reporting Summary linked to this article.

## Supplementary information

Supplementary Information Supplementary Figs. 1–19.

Reporting Summary

Supplementary Tables Supplementary Tables 1–9.

## Source data

Source Data Fig. 2 Statistical source data.

Source Data Fig. 3 Statistical source data.

Source Data Fig. 4 Statistical source data.

Source Data Fig. 5 Statistical source data.

Source Data Fig. 6 Statistical source data.

Source Data Extended Data Fig. 1 Statistical source data.

Source Data Extended Data Fig. 2 Statistical source data.

Source Data Extended Data Fig. 3 Statistical source data.

Source Data Extended Data Fig. 4 Statistical source data.

Source Data Extended Data Fig. 5 Statistical source data.

Source Data Extended Data Fig. 6 Statistical source data.

Source Data Extended Data Fig. 7 Statistical source data.

## Data Availability

The RNA-seq data of human CTEPH cohorts and the control participants have been submitted to the Gene Expression Omnibus under accession number GSE249697. All data related to the findings of this study are available in the Article and Supplementary Information. Source data are provided with this paper.

## Code availability

The code for this study is available via GitHub at https://github.com/loosolab/Code_Jafari_et_al_2024_cteph_rv_reverse_remodeling.

## References

[1] Benza, R. L. et al. Predicting survival in pulmonary arterial hypertension: insights from the Registry to Evaluate Early and Long-Term Pulmonary Arterial Hypertension Disease Management (REVEAL). *Circulation* **122**, 164–172 (2010). 20585012 10.1161/CIRCULATIONAHA.109.898122

[2] Voelkel, N. F. et al. Right ventricular function and failure: report of a National Heart, Lung, and Blood Institute working group on cellular and molecular mechanisms of right heart failure. *Circulation* **114**, 1883–1891 (2006). 17060398 10.1161/CIRCULATIONAHA.106.632208

[3] Iino, M., Dymarkowski, S., Chaothawee, L., Delcroix, M. & Bogaert, J. Time course of reversed cardiac remodeling after pulmonary endarterectomy in patients with chronic pulmonary thromboembolism. *Eur. Radiol.* **18**, 792–799 (2008). 18094973 10.1007/s00330-007-0829-1

[4] Berman, M. et al. Right ventricular reverse remodeling after pulmonary endarterectomy: magnetic resonance imaging and clinical and right heart catheterization assessment. *Pulm. Circ.* **4**, 36–44 (2014). 25006419 10.1086/674884 PMC4070764

[5] di Salvo, T. G. et al. Right ventricular myocardial biomarkers in human heart failure. *J. Card. Fail.* **21**, 398–411 (2015). 25725476 10.1016/j.cardfail.2015.02.005 PMC6482959

[6] Boucherat, O. et al. Identification of LTBP-2 as a plasma biomarker for right ventricular dysfunction in human pulmonary arterial hypertension. *Nat. Cardiovasc. Res.* **1**, 748–760 (2022). 39196085 10.1038/s44161-022-00113-w

[7] Khassafi, F. et al. Transcriptional profiling unveils molecular subgroups of adaptive and maladaptive right ventricular remodeling in pulmonary hypertension. *Nat. Cardiovasc. Res.* **2**, 917–936 (2023). 39196250 10.1038/s44161-023-00338-3 PMC11358157

[8] Humbert, M. et al. 2022 ESC/ERS Guidelines for the diagnosis and treatment of pulmonary hypertension: developed by the task force for the diagnosis and treatment of pulmonary hypertension of the European Society of Cardiology (ESC) and the European Respiratory Society (ERS). Endorsed by the International Society for Heart and Lung Transplantation (ISHLT) and the European Reference Network on rare respiratory diseases (ERN-LUNG). *Eur. Heart J.* **43**, 3618–3731 (2022). 36821743 10.1093/eurheartj/ehad005

[9] McCabe, C. et al. Right ventricular dysfunction in chronic thromboembolic obstruction of the pulmonary artery: a pressure–volume study using the conductance catheter. *J. Appl. Physiol.* **116**, 355–363 (2014). 24356516 10.1152/japplphysiol.01123.2013 PMC3921352

[10] Hardziyenka, M. et al. Right ventricular failure following chronic pressure overload is associated with reduction in left ventricular mass: evidence for atrophic remodeling. *J. Am. Coll. Cardiol.* **57**, 921–928 (2011). 21329838 10.1016/j.jacc.2010.08.648

[11] Mayer, E. et al. Surgical management and outcome of patients with chronic thromboembolic pulmonary hypertension: results from an international prospective registry. *J. Thorac. Cardiovasc. Surg.* **141**, 702–710 (2011). 21335128 10.1016/j.jtcvs.2010.11.024

[12] Maschke, S. K. et al. MRI-derived regional biventricular function in patients with chronic thromboembolic pulmonary hypertension before and after pulmonary endarterectomy. *Acad. Radiol.* **25**, 1540–1547 (2018). 29730148 10.1016/j.acra.2018.04.002

[13] Surie, S. et al. Effect of pulmonary endarterectomy for chronic thromboembolic pulmonary hypertension on stroke volume response to exercise. *Am. J. Cardiol.* **114**, 136–140 (2014). 24819907 10.1016/j.amjcard.2014.04.016

[14] Reesink, H. J. et al. Reverse right ventricular remodeling after pulmonary endarterectomy in patients with chronic thromboembolic pulmonary hypertension: utility of magnetic resonance imaging to demonstrate restoration of the right ventricle. *J. Thorac. Cardiovasc. Surg.* **133**, 58–64 (2007). 17198781 10.1016/j.jtcvs.2006.09.032

[15] Maybaum, S. et al. Cardiac improvement during mechanical circulatory support: a prospective multicenter study of the LVAD Working Group. *Circulation* **115**, 2497–2505 (2007). 17485581 10.1161/CIRCULATIONAHA.106.633180

[16] Barbone, A. et al. Comparison of right and left ventricular responses to left ventricular assist device support in patients with severe heart failure: a primary role of mechanical unloading underlying reverse remodeling. *Circulation* **104**, 670–675 (2001). 11489773 10.1161/hc3101.093903

[17] Kim, G. H., Uriel, N. & Burkhoff, D. Reverse remodelling and myocardial recovery in heart failure. *Nat. Rev. Cardiol.* **15**, 83–96 (2018). 28933783 10.1038/nrcardio.2017.139

[18] Frederiksen, C. A. et al. Reverse remodeling of tricuspid valve morphology and function in chronic thromboembolic pulmonary hypertension patients following pulmonary thromboendarterectomy: a cardiac magnetic resonance imaging and invasive hemodynamic study. *BMC Cardiovasc. Disord.* **21**, 450 (2021). 34535073 10.1186/s12872-021-02248-3 PMC8447771

[19] D’Armini, A. M. et al. Reverse right ventricular remodeling after pulmonary endarterectomy. *J. Thorac. Cardiovasc. Surg.* **133**, 162–168 (2007). 17198805 10.1016/j.jtcvs.2006.08.059

[20] Sweet, M. E. et al. Transcriptome analysis of human heart failure reveals dysregulated cell adhesion in dilated cardiomyopathy and activated immune pathways in ischemic heart failure. *BMC Genomics* **19**, 812 (2018). 30419824 10.1186/s12864-018-5213-9 PMC6233272

[21] Ritchie, M. E. et al. limma powers differential expression analyses for RNA-sequencing and microarray studies. *Nucleic Acids Res.* **43**, e47 (2015). 25605792 10.1093/nar/gkv007 PMC4402510

[22] Ramirez Flores, R. O. et al. Consensus transcriptional landscape of human end-stage heart failure. *J. Am. Heart Assoc.* **10**, e019667 (2021). 33787284 10.1161/JAHA.120.019667 PMC8174362

[23] Boehm, M. et al. Delineating the molecular and histological events that govern right ventricular recovery using a novel mouse model of pulmonary artery de-banding. *Cardiovasc. Res.* **116**, 1700–1709 (2020). 31738411 10.1093/cvr/cvz310 PMC7643543

[24] Litviňuková, M. et al. Cells of the adult human heart. *Nature* **588**, 466–472 (2020). 32971526 10.1038/s41586-020-2797-4 PMC7681775

[25] Kanemaru, K. et al. Spatially resolved multiomics of human cardiac niches. *Nature* **619**, 801–810 (2023). 37438528 10.1038/s41586-023-06311-1 PMC10371870

[26] Uhlén, M. et al. Proteomics. Tissue-based map of the human proteome. *Science* **347**, 1260419 (2015). 25613900 10.1126/science.1260419

[27] Lüönd, F. et al. Hierarchy of TGFβ/SMAD, Hippo/YAP/TAZ, and Wnt/β-catenin signaling in melanoma phenotype switching. *Life Sci. Alliance* **5**, e202101010 (2022). 34819356 10.26508/lsa.202101010 PMC8616544

[28] Delcroix, M. et al. Long-term outcome of patients with chronic thromboembolic pulmonary hypertension: results from an International Prospective Registry. *Circulation* **133**, 859–871 (2016). 26826181 10.1161/CIRCULATIONAHA.115.016522

[29] Andersen, S., Nielsen-Kudsk, J. E., Vonk Noordegraaf, A. & de Man, F. S. Right ventricular fibrosis. *Circulation* **139**, 269–285 (2019). 30615500 10.1161/CIRCULATIONAHA.118.035326

[30] Al-Qazazi, R. et al. Macrophage-NLRP3 activation promotes right ventricle failure in pulmonary arterial hypertension. *Am. J. Respir. Crit. Care Med.* **206**, 608–624 (2022). 35699679 10.1164/rccm.202110-2274OC PMC9716901

[31] Alabed, S. et al. Myocardial T1-mapping and extracellular volume in pulmonary arterial hypertension: a systematic review and meta-analysis. *Magn. Reson. Imaging* **79**, 66–75 (2021). 33745961 10.1016/j.mri.2021.03.011

[32] Chen, Y. et al. Impact of epicardial adipose tissue on right cardiac function and prognosis in pulmonary arterial hypertension. *Chest* **165**, 1211–1223 (2024). 38040053 10.1016/j.chest.2023.11.039

[33] Brittain, E. L. et al. Fatty acid metabolic defects and right ventricular lipotoxicity in human pulmonary arterial hypertension. *Circulation* **133**, 1936–1944 (2016). 27006481 10.1161/CIRCULATIONAHA.115.019351 PMC4870107

[34] Iacobellis, G. Epicardial adipose tissue in contemporary cardiology. *Nat. Rev. Cardiol.* **19**, 593–606 (2022). 35296869 10.1038/s41569-022-00679-9 PMC8926097

[35] Koepp, K. E., Obokata, M., Reddy, Y. N. V., Olson, T. P. & Borlaug, B. A. Hemodynamic and functional impact of epicardial adipose tissue in heart failure with preserved ejection fraction. *JACC Heart Fail.* **8**, 657–666 (2020). 32653449 10.1016/j.jchf.2020.04.016 PMC7395878

[36] Sun, X. Q., Abbate, A. & Bogaard, H. J. Role of cardiac inflammation in right ventricular failure. *Cardiovasc. Res.* **113**, 1441–1452 (2017). 28957536 10.1093/cvr/cvx159

[37] Saito, T. et al. Effects of pulmonary endarterectomy on pulmonary hemodynamics in chronic thromboembolic pulmonary hypertension, evaluated by interventricular septum curvature. *Pulm. Circ.* **10**, 2045894019897502 (2020). 32206304 10.1177/2045894019897502 PMC7074512

[38] Sugiura, T. et al. Role of 320-slice CT imaging in the diagnostic workup of patients with chronic thromboembolic pulmonary hypertension. *Chest* **143**, 1070–1077 (2013). 23100061 10.1378/chest.12-0407

[39] Franco, D. et al. Left and right ventricular contributions to the formation of the interventricular septum in the mouse heart. *Dev. Biol.* **294**, 366–375 (2006). 16677630 10.1016/j.ydbio.2006.02.045

[40] Lankeit, M. et al. Pulmonary endarterectomy in chronic thromboembolic pulmonary hypertension. *J. Heart Lung Transplant.* **37**, P250–P258 (2017). 10.1016/j.healun.2017.06.011 28750932

[41] Lang, I. M., Dorfmüller, P. & Vonk Noordegraaf, A. The pathobiology of chronic thromboembolic pulmonary hypertension. *Ann. Am. Thorac. Soc.* **13**, S215–S221 (2016). 27571003 10.1513/AnnalsATS.201509-620AS

[42] Kriechbaum, S. D. et al. N-terminal pro-B-type natriuretic peptide for monitoring after balloon pulmonary angioplasty for chronic thromboembolic pulmonary hypertension. *J. Heart Lung Transplant.* **37**, 639–646 (2018). 29329761 10.1016/j.healun.2017.12.006

[43] Kriechbaum, S. D. et al. Cardiac biomarkers as indicators of right ventricular dysfunction and recovery in chronic thromboembolic pulmonary hypertension patients after balloon pulmonary angioplasty therapy—a cardiac magnetic resonance imaging cohort study. *Pulm. Circ.* **11**, 20458940211056500 (2021). 34917333 10.1177/20458940211056500 PMC8669885

[44] Kriechbaum, S. D. et al. Mid-regional pro-atrial natriuretic peptide and copeptin as indicators of disease severity and therapy response in CTEPH. *ERJ Open Res.* **6**, 00356–02020 (2020). 33263045 10.1183/23120541.00356-2020 PMC7682678

[45] Wiedenroth, C. B. et al. Riociguat and balloon pulmonary angioplasty improve prognosis in patients with inoperable chronic thromboembolic pulmonary hypertension. *J. Heart Lung Transplant.* **42**, 134–139 (2023). 36257870 10.1016/j.healun.2022.08.011

[46] Roller, F. C. et al. Correlation of native T1 mapping with right ventricular function and pulmonary haemodynamics in patients with chronic thromboembolic pulmonary hypertension before and after balloon pulmonary angioplasty. *Eur. Radiol.* **29**, 1565–1573 (2019). 30159622 10.1007/s00330-018-5702-x

[47] Roller, F. C. et al. Effects of BPA on right ventricular mechanical dysfunction in patients with inoperable CTEPH—a cardiac magnetic resonance study. *Eur. J. Radiol.* **147**, 110111 (2022). 34952330 10.1016/j.ejrad.2021.110111

[48] Bogomolovas, J. et al. Induction of Ankrd1 in dilated cardiomyopathy correlates with the heart failure progression. *Biomed. Res. Int.* **2015**, 273936 (2015). 25961010 10.1155/2015/273936 PMC4415747

[49] Ling, S. S. M., Chen, Y.-T., Wang, J., Richards, A. M. & Liew, O. W. Ankyrin repeat domain 1 protein: a functionally pleiotropic protein with cardiac biomarker potential. *Int. J. Mol. Sci.* **18**, 1362 (2017). 28672880 10.3390/ijms18071362 PMC5535855

[50] Murphy, N. P., Lubbers, E. R. & Mohler, P. J. Advancing our understanding of AnkRD1 in cardiac development and disease. *Cardiovasc. Res.* **116**, 1402–1404 (2020). 32186710 10.1093/cvr/cvaa063 PMC7314634

[51] Ghosh, A. K. & Vaughan, D. E. PAI-1 in tissue fibrosis. *J. Cell. Physiol.* **227**, 493–507 (2012). 21465481 10.1002/jcp.22783 PMC3204398

[52] Galiè, N. et al. 2015 ESC/ERS Guidelines for the diagnosis and treatment of pulmonary hypertension: The Joint Task Force for the Diagnosis and Treatment of Pulmonary Hypertension of the European Society of Cardiology (ESC) and the European Respiratory Society (ERS): endorsed by: Association for European Paediatric and Congenital Cardiology (AEPC), International Society for Heart and Lung Transplantation (ISHLT). *Eur. Heart J.* **37**, 67–119 (2016). 26320113 10.1093/eurheartj/ehv317

[53] Andrews, S. FastQC: a quality control tool for high throughput sequence data. https://www.bioinformatics.babraham.ac.uk/projects/fastqc/ (2010).

[54] Bolger, A. M., Lohse, M. & Usadel, B. Trimmomatic: a flexible trimmer for Illumina sequence data. *Bioinformatics* **30**, 2114–2120 (2014). 24695404 10.1093/bioinformatics/btu170 PMC4103590

[55] Dobin, A. et al. STAR: ultrafast universal RNA-seq aligner. *Bioinformatics* **29**, 15–21 (2013). 23104886 10.1093/bioinformatics/bts635 PMC3530905

[56] Liao, Y., Smyth, G. K. & Shi, W. featureCounts: an efficient general purpose program for assigning sequence reads to genomic features. *Bioinformatics* **30**, 923–930 (2014). 24227677 10.1093/bioinformatics/btt656

[57] Love, M. I., Huber, W. & Anders, S. Moderated estimation of fold change and dispersion for RNA-seq data with DESeq2. *Genome Biol.* **15**, 550 (2014). 25516281 10.1186/s13059-014-0550-8 PMC4302049

[58] Lê, S., Josse, J. & Husson, F. FactoMineR: an R package for multivariate analysis. *J. Stat. Softw.* **25**, 1–18 (2008).

[59] Leek, J. T., Johnson, W. E., Parker, H. S., Jaffe, A. E. & Storey, J. D. The sva package for removing batch effects and other unwanted variation in high-throughput experiments. *Bioinformatics* **28**, 882–883 (2012). 22257669 10.1093/bioinformatics/bts034 PMC3307112

[60] Gu, Z., Eils, R. & Schlesner, M. Complex heatmaps reveal patterns and correlations in multidimensional genomic data. *Bioinformatics* **32**, 2847–2849 (2016). 27207943 10.1093/bioinformatics/btw313

[61] Gu, Z. Complex heatmap visualization. *Imeta* **1**, e43 (2022). 38868715 10.1002/imt2.43 PMC10989952

[62] Zhou, Y. et al. Metascape provides a biologist-oriented resource for the analysis of systems-level datasets. *Nat. Commun.* **10**, 1523 (2019). 30944313 10.1038/s41467-019-09234-6 PMC6447622

[63] R Core Team R: A Language and Environment for Statistical Computing (R Foundation for Statistical Computing, 2024); http://www.R-project.org/

[64] Kojonazarov, B. et al. Single- versus multiple-beat measurement of right ventricular function in rodents. *Am. J. Respir. Cell Mol. Biol.* **71**, 133–135 (2024). 38949324 10.1165/rcmb.2023-0407LE

[65] Pullamsetti, S. S. et al. Lung cancer-associated pulmonary hypertension: role of microenvironmental inflammation based on tumor cell-immune cell cross-talk. *Sci. Transl. Med.* **9**, eaai9048 (2017). 29141888 10.1126/scitranslmed.aai9048

