# Supplementary material for: Transcriptional changes of the extracellular matrix in chronic thromboembolic pulmonary hypertension govern right ventricle remodeling and recovery
Source: Nat Cardiovasc Res. 2025 Jul 4;4(7):857–75. doi: 10.1038/s44161-025-00672-8 (PMC12259468; doi:10.1038/s44161-025-00672-8)
Supplement: Supplementary file 1 — Supplementary Figs. 1–19. [file 44161_2025_672_MOESM1_ESM.pdf]

nature cardiovascular research

Supplementary information https://doi.org/10.1038/s44161-025-00672-8

Transcriptional changes of the extracellular matrix in chronic thromboembolic pulmonary hypertension govern right ventricle remodeling and recovery

In the format provided by the authors and unedited

Supplementary Figure 1.	Gene expression correlation between all the RV samples in the exploratory of patients with CTEPH
Supplementary Figure 2.	Heatmap of top significant DEGs in the exploratory cohort of patients with CTEPH prior to PEA
Supplementary Figure 3.	Pathway enrichment analysis in the RV of the exploratory cohort of patients with CTEPH
Supplementary Figure 4.	Gene expression correlation between the RV of CTEPH patients in the confirmatory cohort and the control RV
Supplementary Figure 5.	Heatmap of top-50 DEGs in the RV of CTEPH patients and controls
Supplementary Figure 6.	Gene expression profile in RV of patients with CTEPH and Left ventricle
Supplementary Figure 7.	Gene expression correlation between all RV and septum samples in the confirmatory cohorts of CTEPH patients
Supplementary Figure 8.	Heatmap of top-50 DEGs in the RV and septum of CTEPH patients
Supplementary Figure 9.	Pathway enrichment analysis in the RV of the PAB versus Sham and PAB versus Rapide
Supplementary Figure 10.	Correlation of *SERPINE1*, *ANKRD1*, and *IL7R* expression in the RV and septum, with the clinical parameters of the confirmatory cohort of patients with CTEPH
Supplementary Figure 11.	*SERPINE1*, *IL7R,* and *ANKRD1* were visualized on single-cell RNA sequencing data from the Human Heart Atlas, version 2
Supplementary Figure 12.	The Heatmaps of correlation matrix with reference transcripts in heart muscle
Supplementary Figure 13.	Expression of *SERPINE1*, *IL7R*, and *ANKRD1* and their protein-protein interactions
Supplementary Figure 14.	Screening of *SERPINE1*, *IL7R*, and *ANKRD1* expression in cardiac cells exposed to pro-fibrotic and pro-inflammatory stimuli
Supplementary Figure 15.	Screening of *SERPINE1*, *IL7R*, and *ANKRD1* expression in cardiac cells exposed to hypoxic conditions
Supplementary Figure 16.	Correlations between *Serpine1*, *Il7R*, and *Ankrd1* mRNA levels and the RV hemodynamics parameters in rat models of MCT-induced PH and PAB
Supplementary Figure 17.	siRNA transfection attenuates the expression of *SERPINE1*, *IL7R*, and *ANKRD1* in HCFs and HCMECs
Supplementary Figure 18.	Effects of siRNA-mediated knockdown of *SERPINE1* and *ANKRD1* in HCFs and HCMECs
Supplementary Figure 19.	Effects of siRNA-mediated knockdown of *SERPINE1, ANKRD1*, and *IL7R* on tube formation and proliferation in HCFs and HCMECs

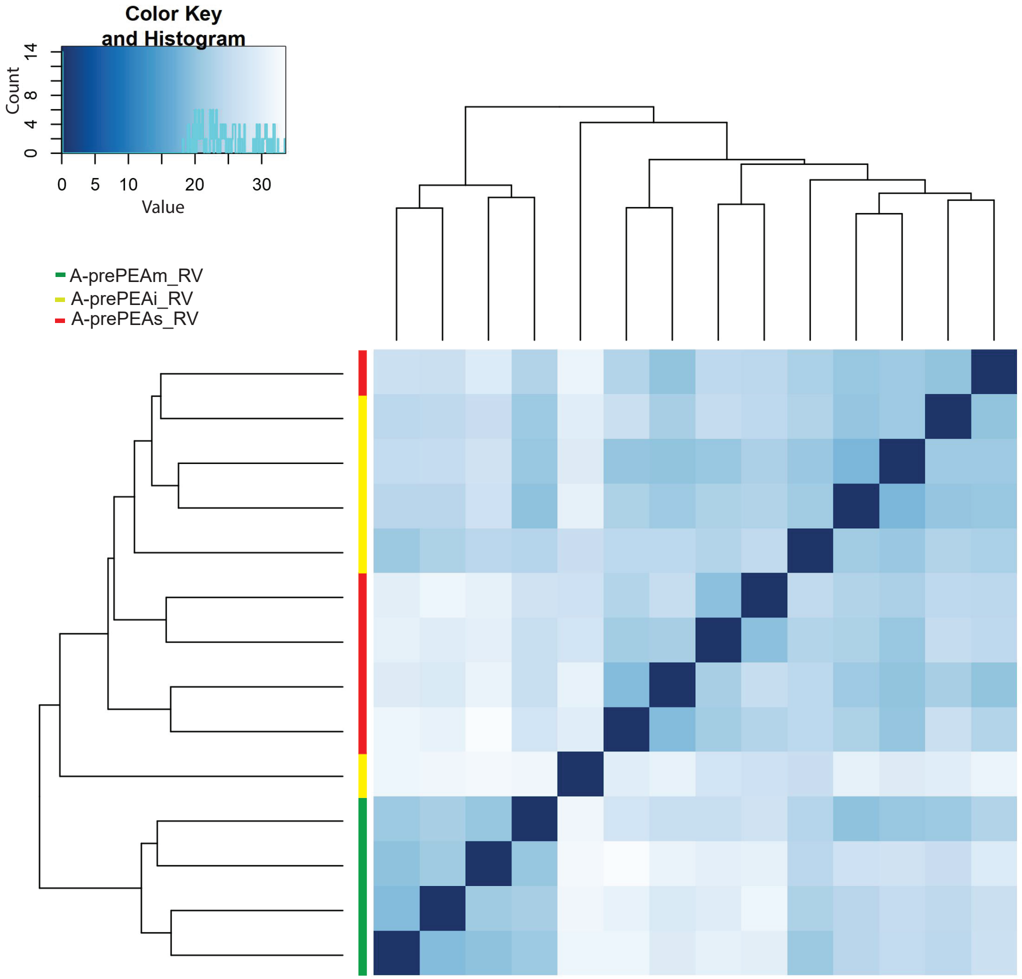

Supplementary Figure 1. Gene expression correlation between all the RV samples in the exploratory cohort of patients with CTEPH. A general overview of the sample similarity for RV samples in the exploratory cohort (A-prePEAm_RV, n = 4, A-prePEAi_RV, n = 5, and A-prePEAs_RV, n = 5) are displayed. DESeq2-normalized counts were regularized, log-transformed, and clustered hierarchically to generate the heatmap.

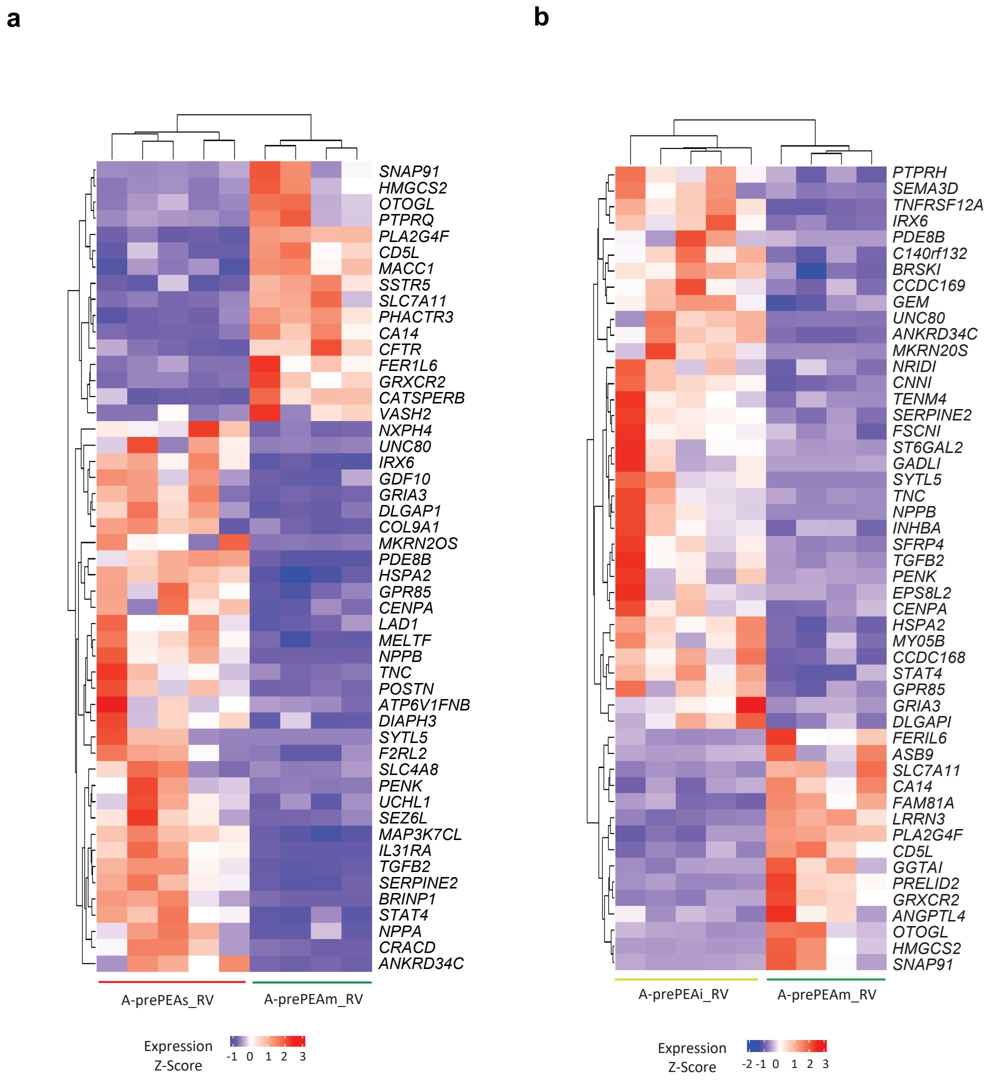

Supplementary Figure 2. Heatmap of top significant DEGs in the exploratory cohort of patients with CTEPH prior to PEA. Heatmaps represent the top 50 significant DEGs (filtered by |log2FC|≥ 0.585; FDR≤ 0.05) of **a**, A-prePEAm_RV versus A-prePEAs_RV, (**b**) A-prePEAm_RV versus A-prePEAi_RV in the exploratory cohort of patients with CTEPH. The heatmaps display the scaled z-score of normalized counts.

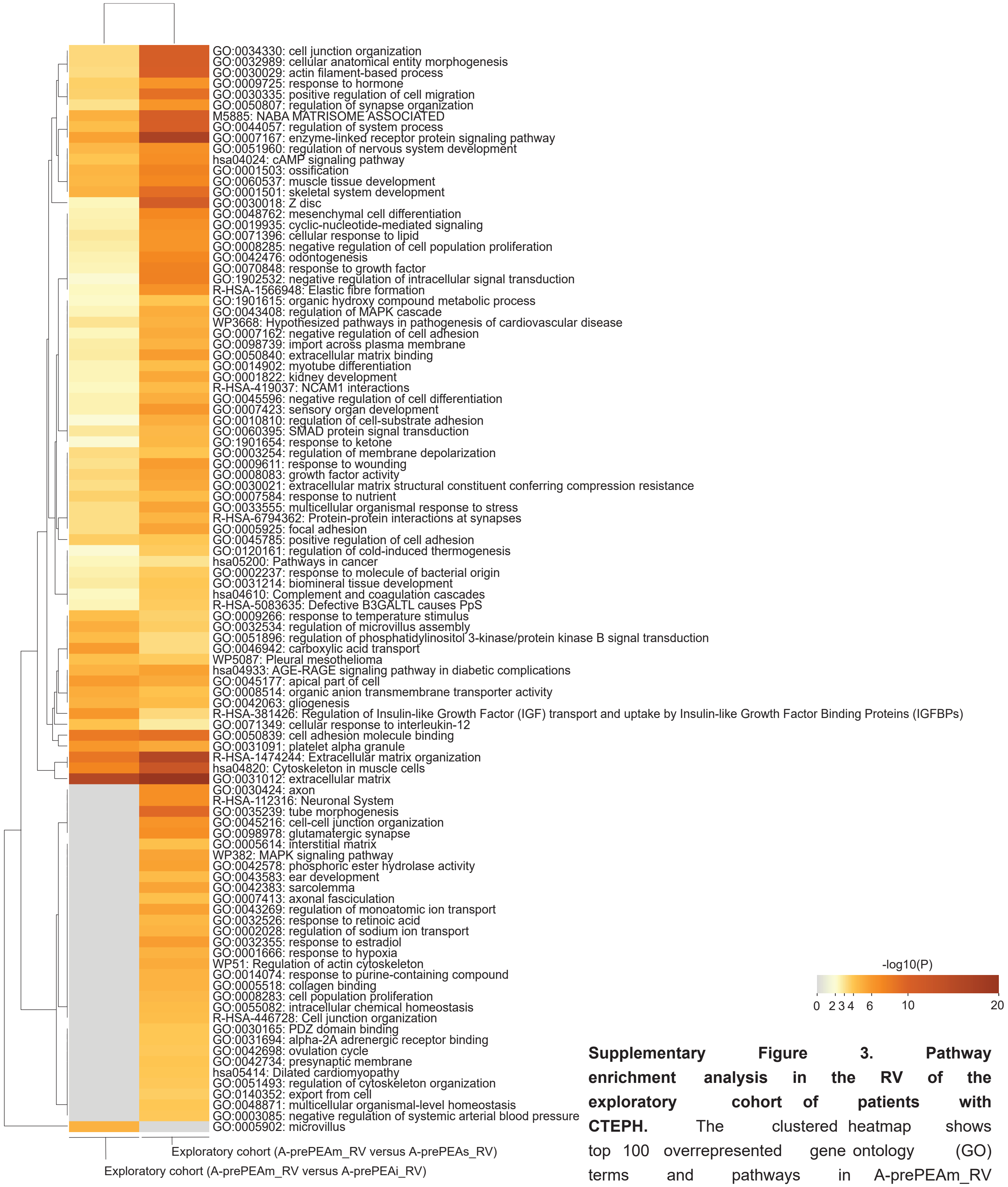

Supplementary Figure 3. Pathway enrichment analysis in the RV of the exploratory cohort of patients with CTEPH. The clustered heatmap shows top 100 overrepresented gene ontology (GO) terms and pathways in A-prePEAm_RV versus A-prePEAi_RV and A-prePEAm_RV versus A-prePEAs_RV.

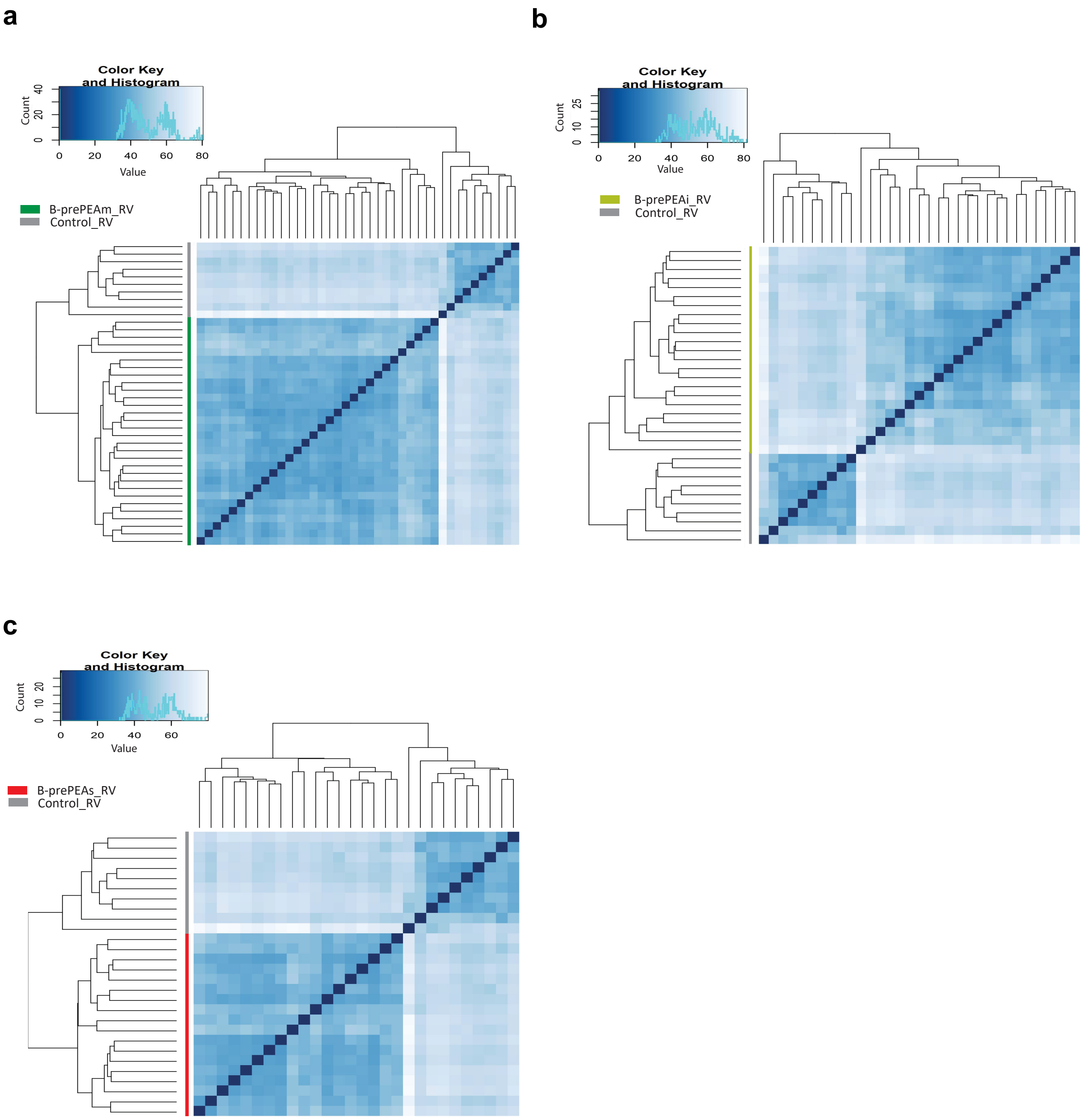

Supplementary Figure 4. Gene expression correlation between the RV of CTEPH patients in the confirmatory cohort and the control RV. Global overview of the sample similarity for the RV of CTEPH patients before PEA and control RV are displayed and each exhibiting high in-group similarity: **a**, B-prePEAm_RV (*n* = 30) and Control_RV (*n* = 10), **b**, B-prePEAi_RV (*n* = 23) and Control_RV (*n* = 10), **c**, B-prePEAs_RV (*n* = 18) and Control_RV (*n* = 10).

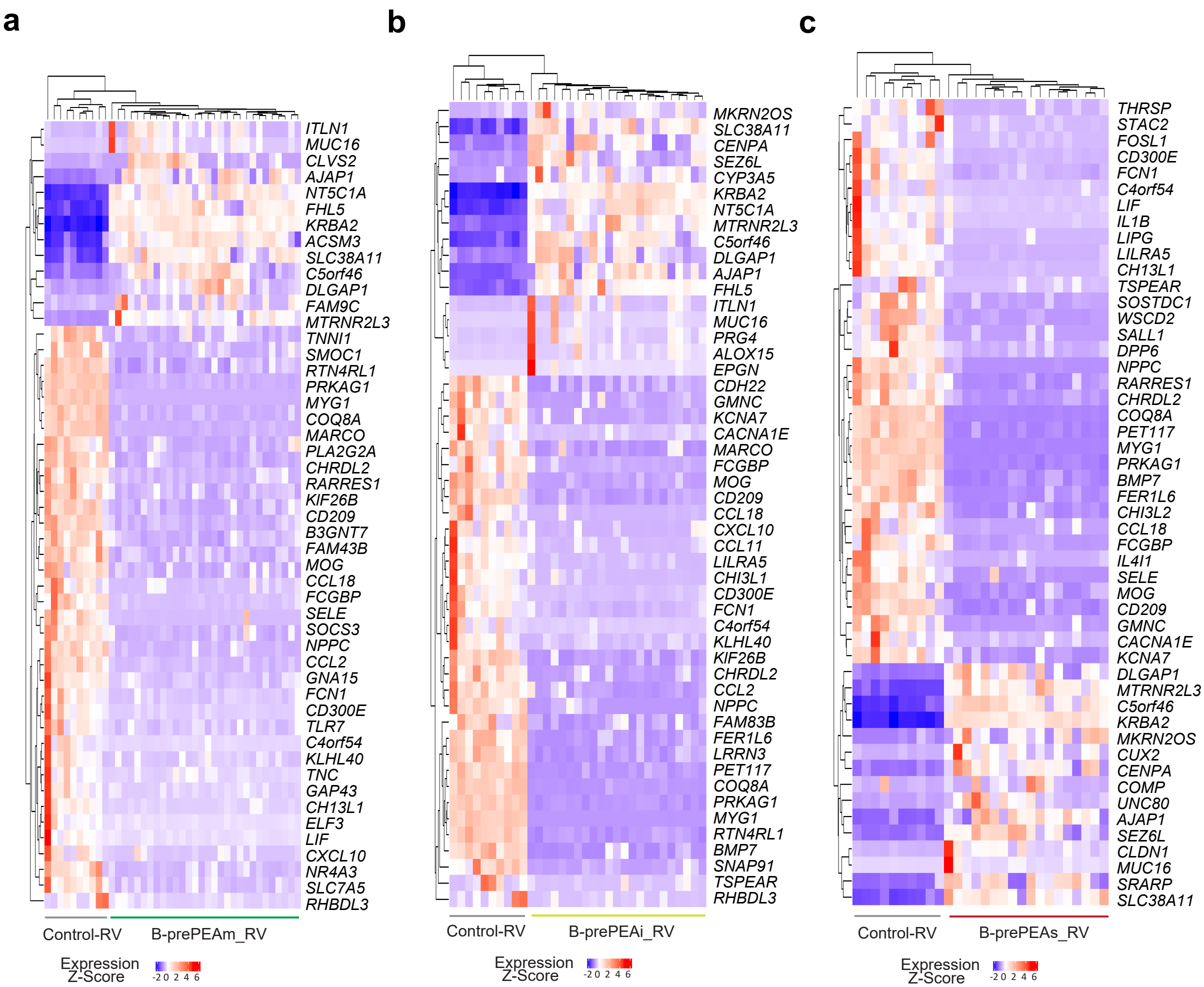

Supplementary Figure 5. Heatmap of top-50 DEGs in the RV of CTEPH patients and controls. Heatmaps display the top 50 significant DEGs (base mean expression≥5; |log2FC|≥ 0.585; FDR≤ 0.05) in **a**, B-prePEAm_RV versus Control_RV, **b**, B-prePEAi_RV versus Control_RV, and **c**, B-prePEAs_RV versus Control_RV. The scaled z-score of normalized counts is shown.

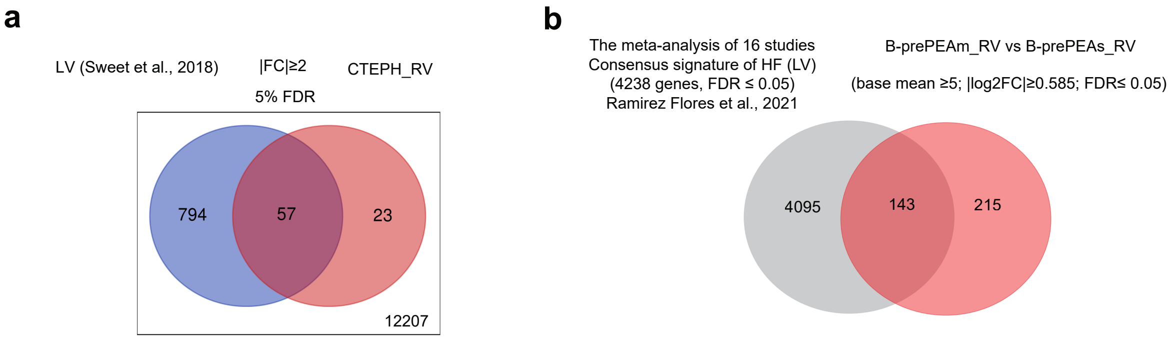

Supplementary Figure 6. Gene expression profile in RV of patients with CTEPH and Left ventricle. a, The gene expression profile of RV in the confirmatory cohort of patients with CTEPH (B-prePEAs_RV versus B-prePEAm_RV) was compared with left ventricle (LV) heart failure [ischemic cardiomyopathy (ICM), and dilated cardiomyopathy (DCM) versus non-failing (NF)]specific genes. In this comparison, B-prePEAm_RV was used as a reference versus B-prePEAs_RV. **b**, The HF-specific genes of the left ventricle (LV) from the meta-analysis were compared to the RV of patients with CTEPH (B-prePEAm_RV versus B-prePEAs_RV).

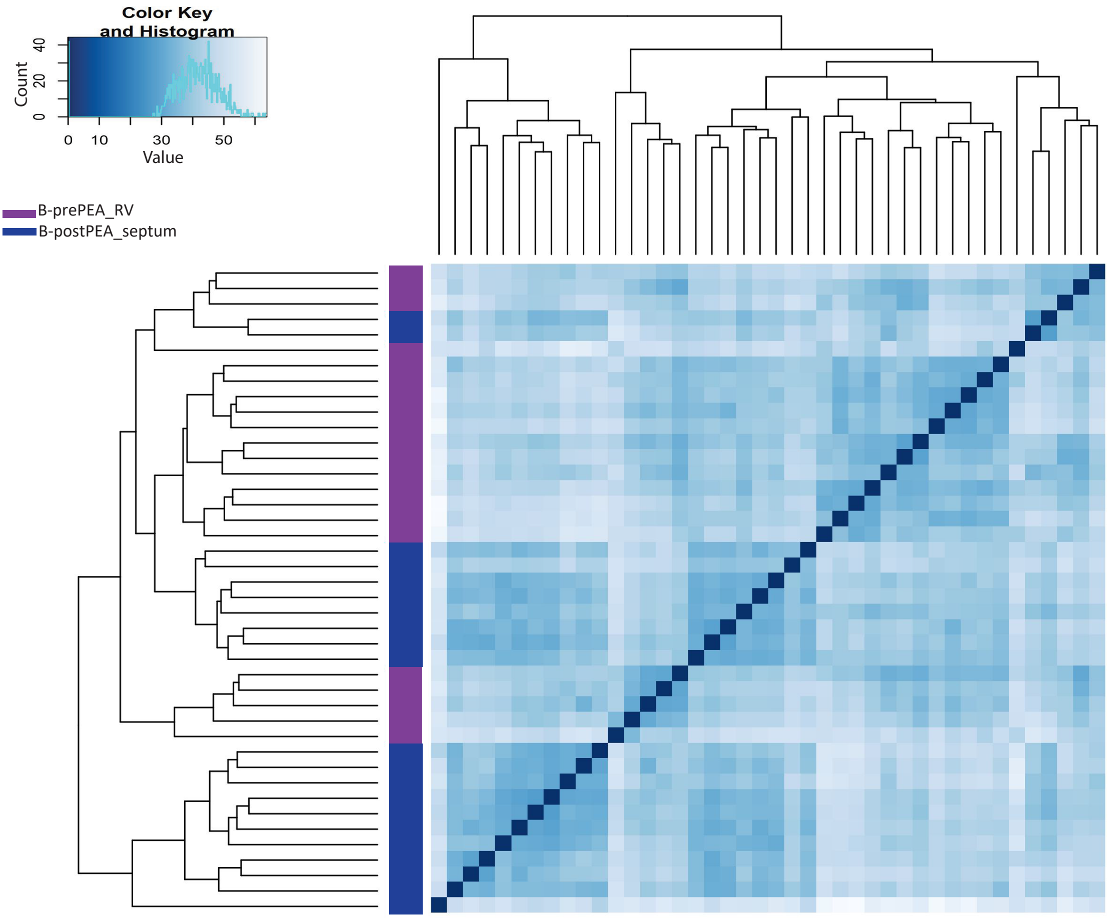

Supplementary Figure 7. Gene expression correlation between all RV and septum samples in the confirmatory cohorts of CTEPH patients. A general overview of the sample similarity between RV samples in the confirmatory cohort (B-prePEA_RV, *n* = 21 and B-postPEA_septum, *n* = 21) are displayed. DESeq2-normalized counts were regularized, log-transformed, and clustered hierarchically to generate the heatmap.

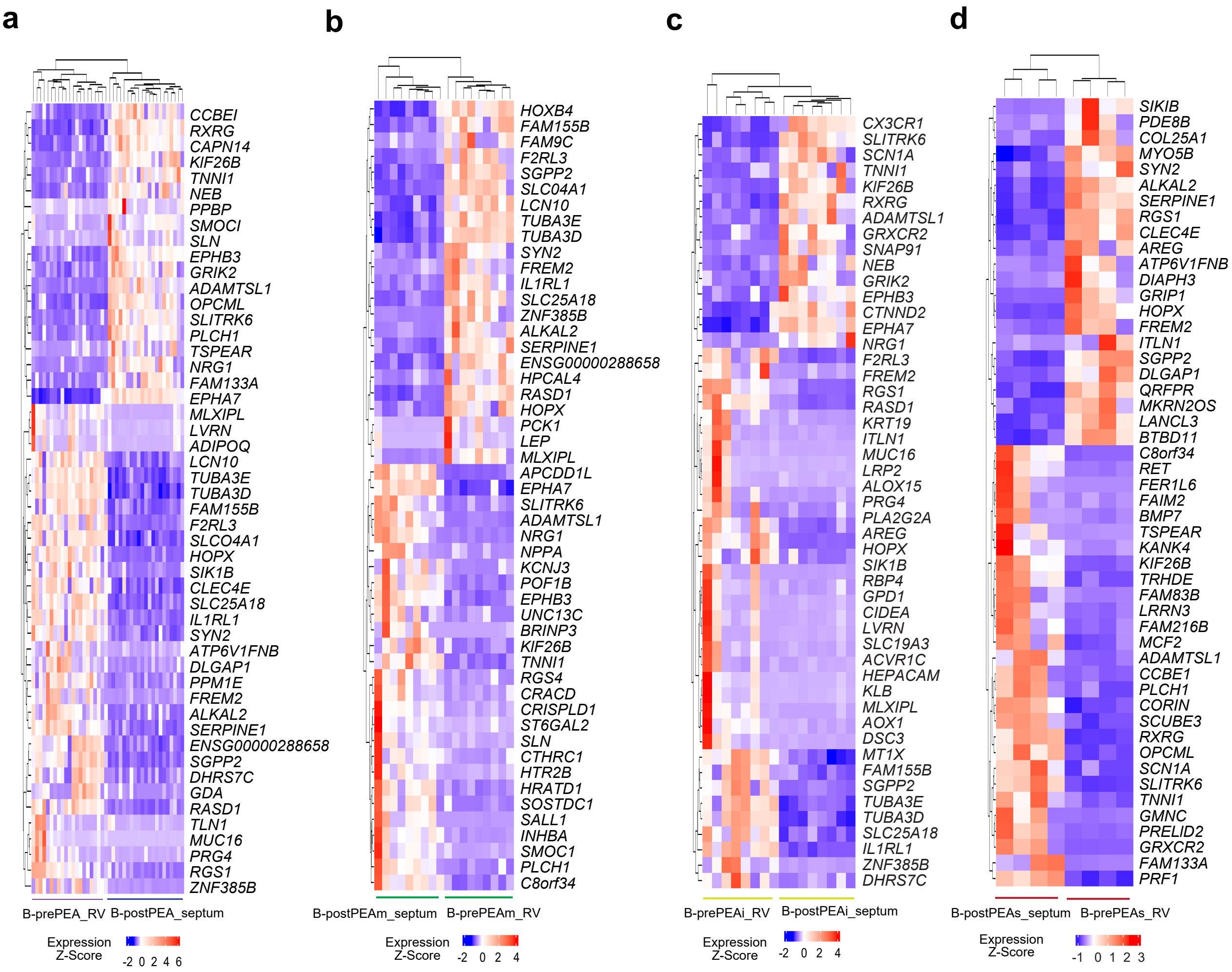

Supplementary Figure 8. Heatmap of top-50 DEGs in the RV and septum of CTEPH patients. Heatmaps represent the top 50 significant DEGs (filtered by base mean ≥5; |log2FC|≥ 0.585; FDR≤ 0.05) in (**a**) B-postPEA_septum versus B-prePEA_RV, (**b**) B-postPEAm_septum versus B-prePEAm_RV, (**c**) B-postPEAi_septum versus B-prePEAi_RV, and (**d**) B-postPEAs_septum versus B-prePEAs_RV of the confirmatory cohort of CTEPH patients. The heatmaps show the scaled z-score of normalized counts..

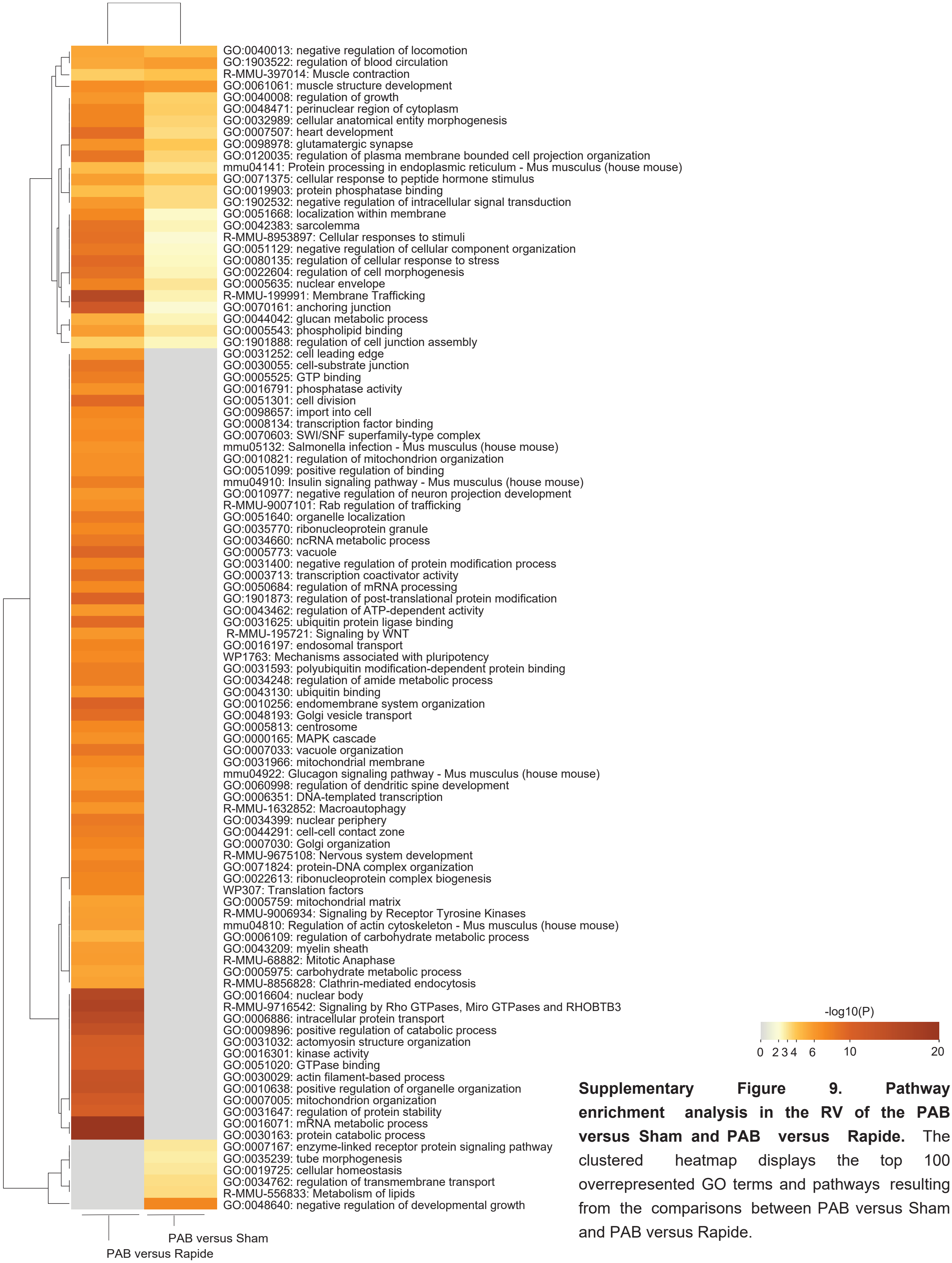

Supplementary Figure 9. Pathway enrichment analysis in the RV of the PAB versus Sham and PAB versus Rapide. The clustered heatmap displays the top 100 overrepresented GO terms and pathways resulting from the comparisons between PAB versus Sham and PAB versus Rapide.

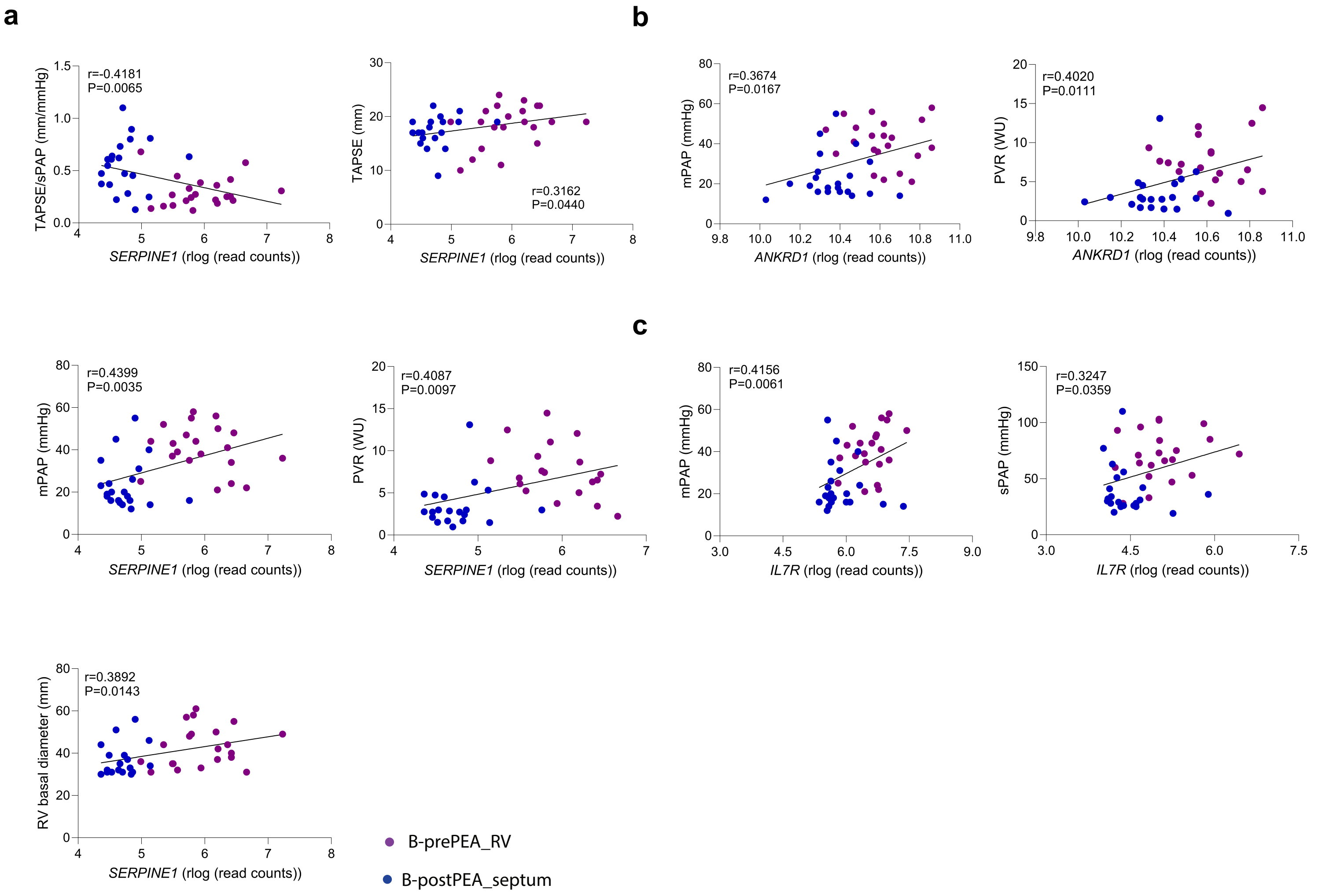

Supplementary Figure 10. Correlation of *SERPINE1*, *ANKRD1*, and *IL7R* expression in the RV and septum, with the clinical parameters of the confirmatory cohort of patients with CTEPH. The correlation between rlog (readcounts) of (**a**) *SERPINE1*, (**b**) *ANKRD1*, and (**c**) *IL7R* with clinical parameters of the patients before PEA (B-prePEA_RV, n = 21) and postPEA (B-postPEA_septum, n = 21) are shown. Pearson's correlation coefficient (r) and its associated two-tailed P value are shown in each graph. Abbreviations: mPAP, mean pulmonary artery pressure; sPAP, systolic pulmonary artery pressure; PVR, pulmonary vascular resistance; TAPSE, Tricuspid annular plane systolic excursion.

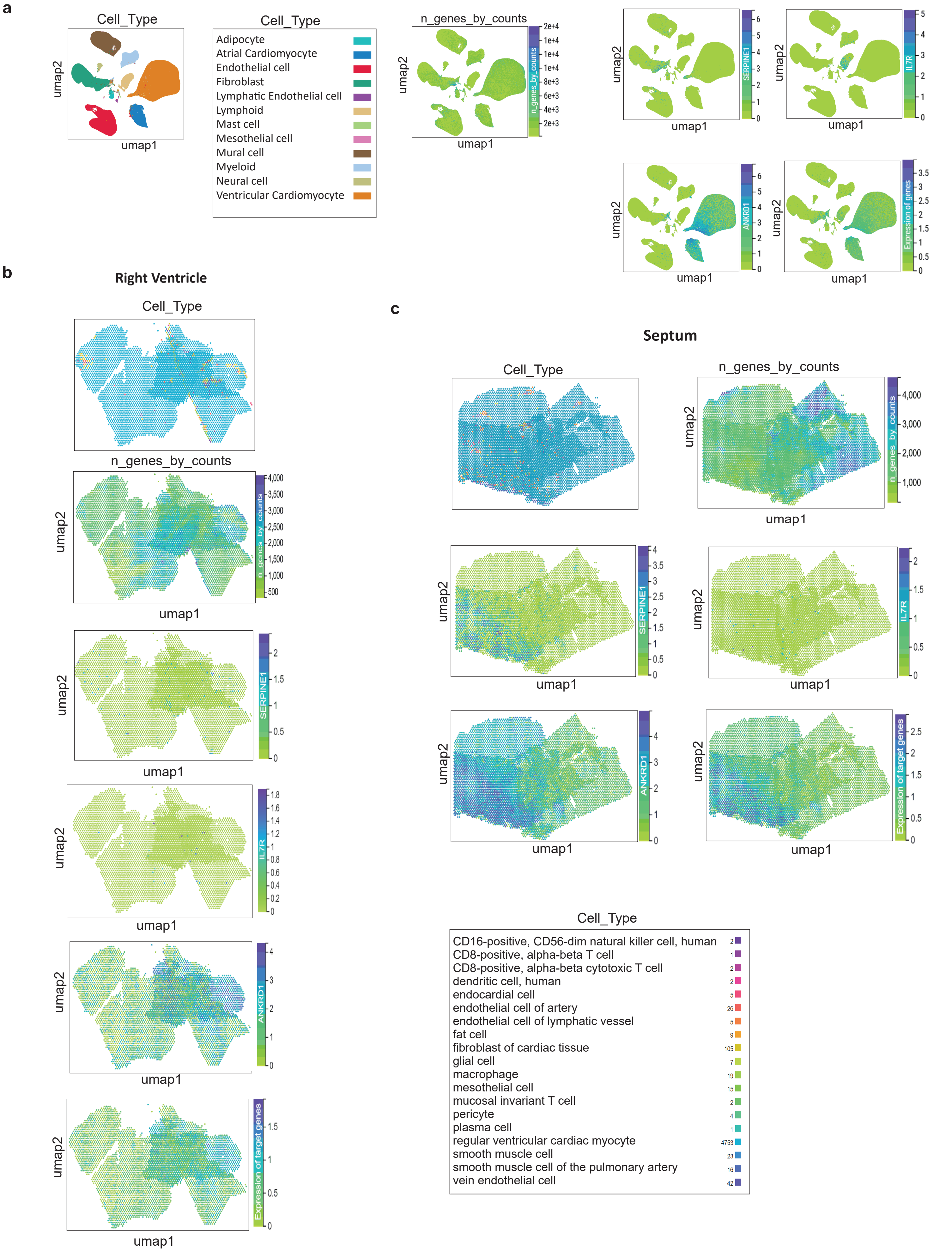

Supplementary Figure 11. *SERPINE1*, *IL7R*, and *ANKRD1* were visualized on single-cell RNA sequencing data from the Human Heart Atlas, version 2. a, The Uniform manifold approximation and projection (UMAP) shows the expression of *SERPINE1*, *IL7R*, and *ANKRD1* across the 704,296 individual cardiac cells. **b**, The expression of *SERPINE1*, *IL7R*, and *ANKRD1* was mapped in three normal human RV tissues (> 55 years old) across the 5039 cells and **c**, four normal human septum tissues (>45 years old) across the 8643 cells. (https://www.heartcellatlas.org/index.html)[24,25].

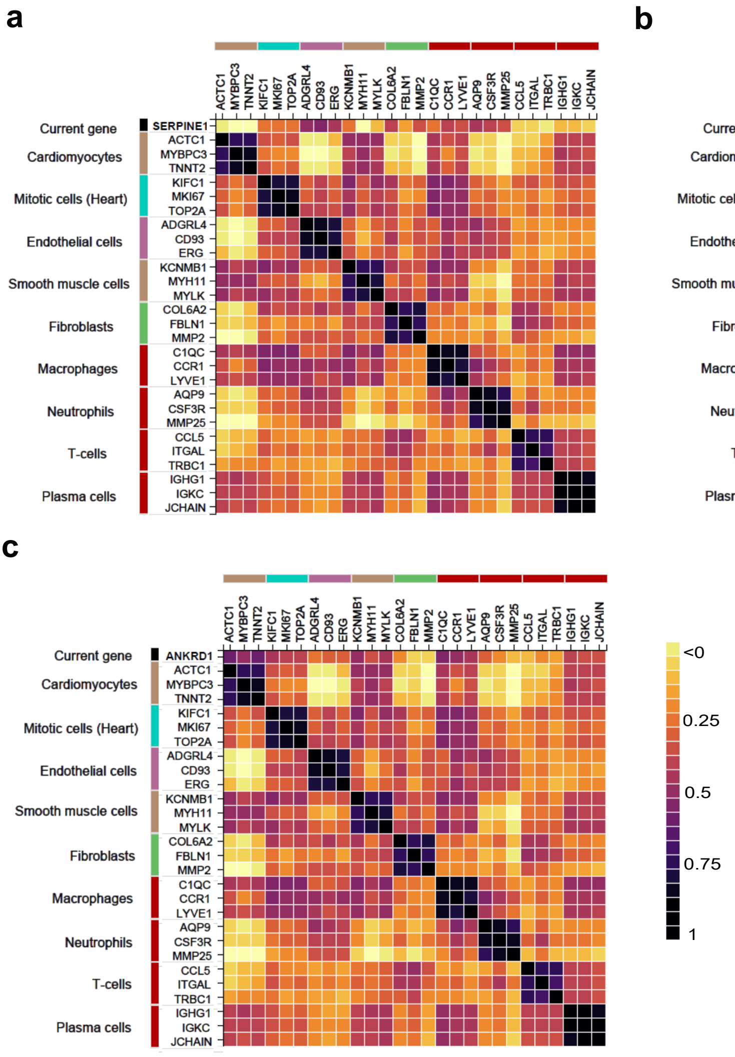

Supplementary Figure 12. The Heatmaps of correlation matrix with reference transcripts in heart muscle. The correlation matrix of, **a**, *SERPINE1* (https://v23.proteinatlas.org/ENSG00000106366-SERPINE1/tissue+cell+type/heart+muscle), **b**, *IL7R* (https://v23.proteinatlas.org/ENSG00000168685-IL7R/tissue+cell+type/heart+muscle), and **c**, *ANKRD1* (https://v23.proteinatlas.org/ENSG00000148677-ANKRD1/tissue+cell+type/heart+muscle), with 3 virtual reference transcripts for each cell type in the heart muscle tissue is shown. The darker squares show higher correlation values.(courtesy of Human Protein Atlas, https://www.proteinatlas.org/)[26].

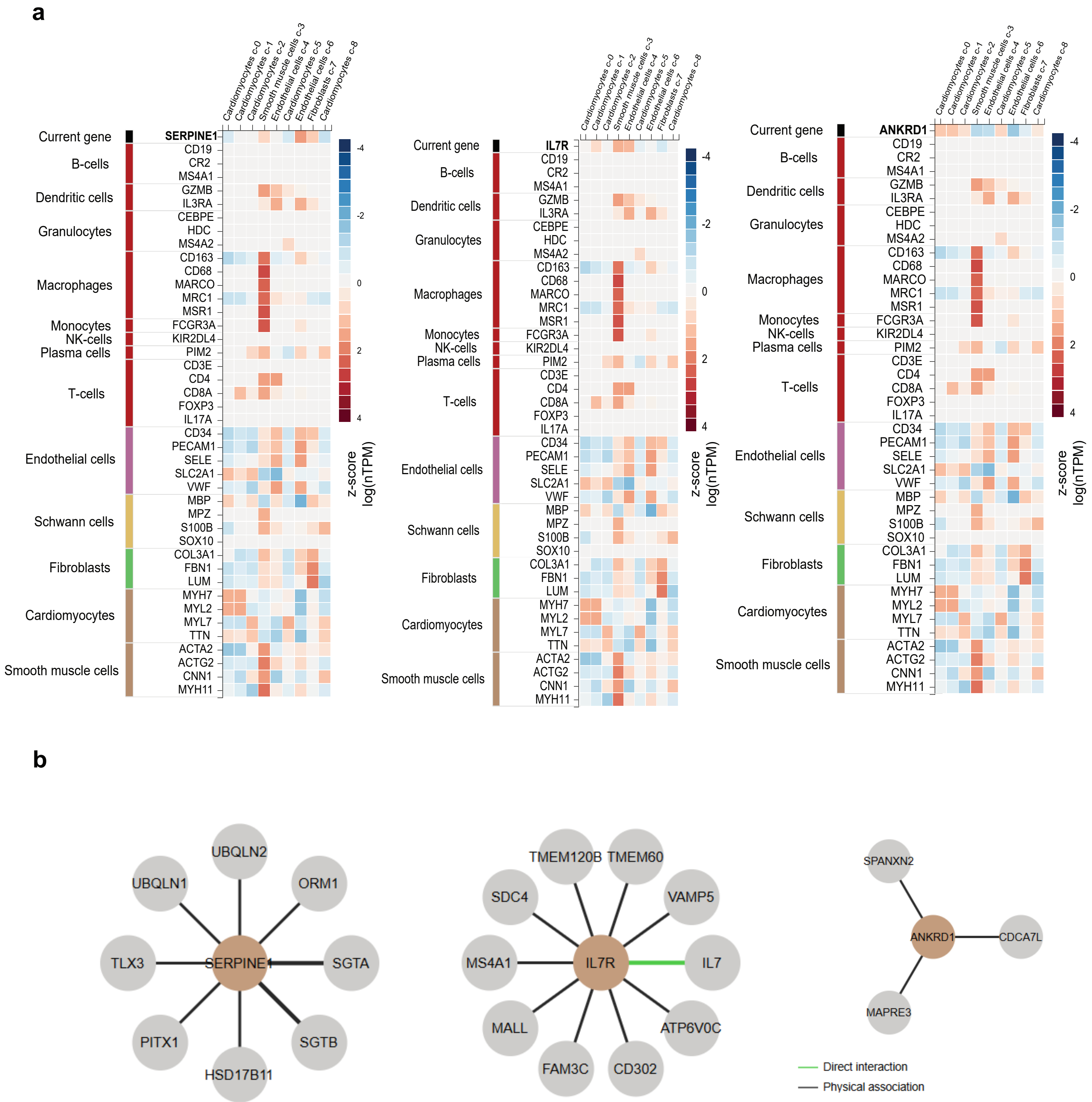

Supplementary Figure 13. Expression of *SERPINE1*, *IL7R*, and *ANKRD1*, and their protein-protein interactions. (**a**) Expression of *SERPINE1* (https://v23.proteinatlas.org/ENSG00000106366-SERPINE1/single+cell+type/heart+muscle), *IL7R* (https://v23.proteinatlas.org/ENSG00000168685-IL7R/single+cell+type/heart+muscle), and *ANKRD1* (https://v23.proteinatlas.org/ENSG00000148677-ANKRD1/single+cell+type/heart+muscle), and the well-known cell type marker in different single-cell type clusters of the heart tissue are displayed. (**b**) protein-protein interactions of SERPINE1 (https://v23.proteinatlas.org/ENSG00000106366-SERPINE1/interaction), IL7R (https://v23.proteinatlas.org/ENSG00000168685-IL7R/interaction), and ANKRD1 (https://v23.proteinatlas.org/ENSG00000148677-ANKRD1/interaction). (Courtesy of Human Protein Atlas, https://www.proteinatlas.org/)[26].

a

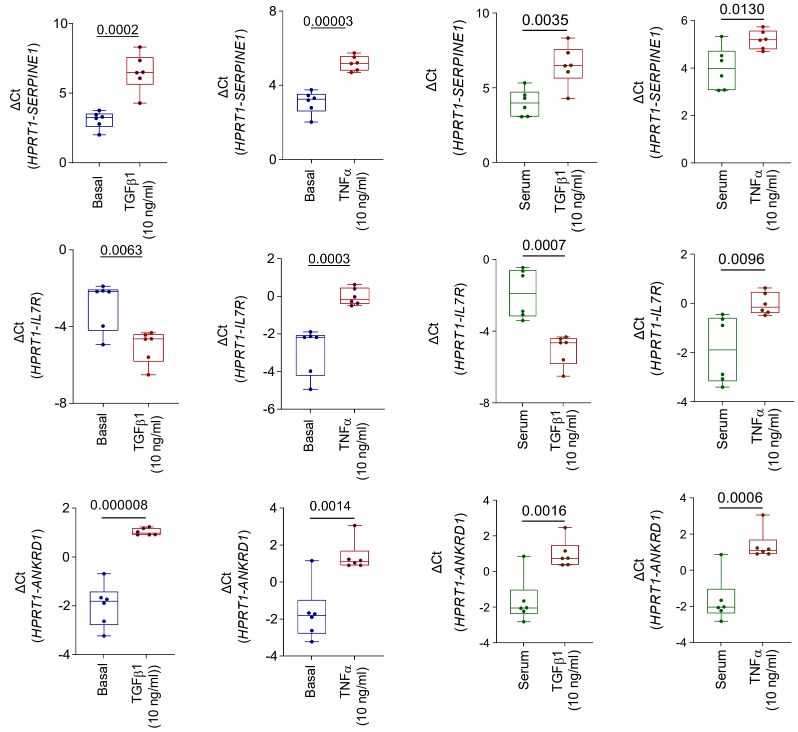

b

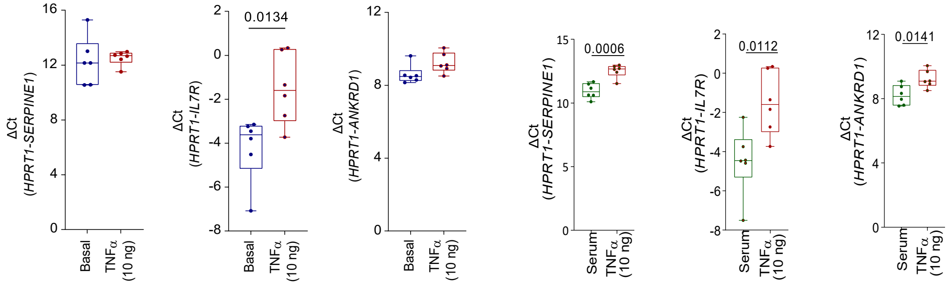

Supplementary Figure 14. Screening of *SERPINE1*, *IL7R*, and *ANKRD1* expression in cardiac cells exposed to pro-fibrotic and pro-inflammatory stimuli. Relative mRNA expression levels of *SERPINE1*, *IL7R*, and *ANKRD1* in **a**, human cardiac fibroblasts (HCFs) and **b**, human cardiac microvascular endothelial cells (HCMECs) after stimulation with TGF-β1 and TNF-α for 48 hours were compared to serum or basal medium by qRT-PCR. *HPRT1* was used as the endogenous control and data are presented as mean ± SEM (n = 3, biological replicates each with two technical replicates; two-tailed unpaired t-test). Boxes show the interquartile range (IQR, 25th to 75th percentile), and the central band represents the median. The whiskers extend to 1.5 times the IQR above and below the box.

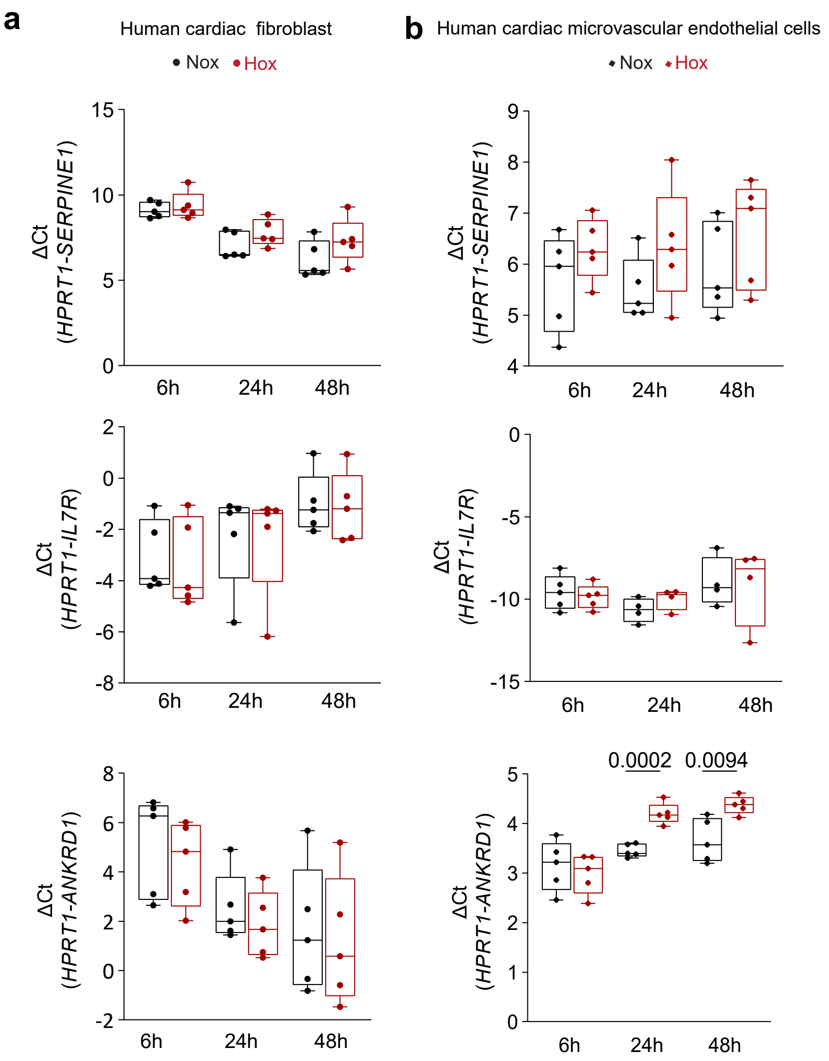

Supplementary Figure 15. Screening of *SERPINE1*, *IL7R*, and *ANKRD1* expression in cardiac cells exposed to hypoxic conditions. Relative mRNA expression levels of *SERPINE1*, *IL7R*, and *ANKRD1* in **a**, HCFs and **b**, HCMECs after exposure to normoxia or hypoxia for 6, 24 and 48 hours. HPRT1 was used as the endogenous control. Data are presented as mean ± SEM. (n = 5 biological replicates for HCFs; n = 3 biological replicates with only two having two technical replicates (for *IL7R* at 24h and 48h, only one biological replicate has two technical replicates) for HCMECs ; two-tailed unpaired t-test). Boxes show the interquartile range (IQR, 25th to 75th percentile), and the central bands indicate the median. The whiskers extend to 1.5 times the IQR above and below the box.

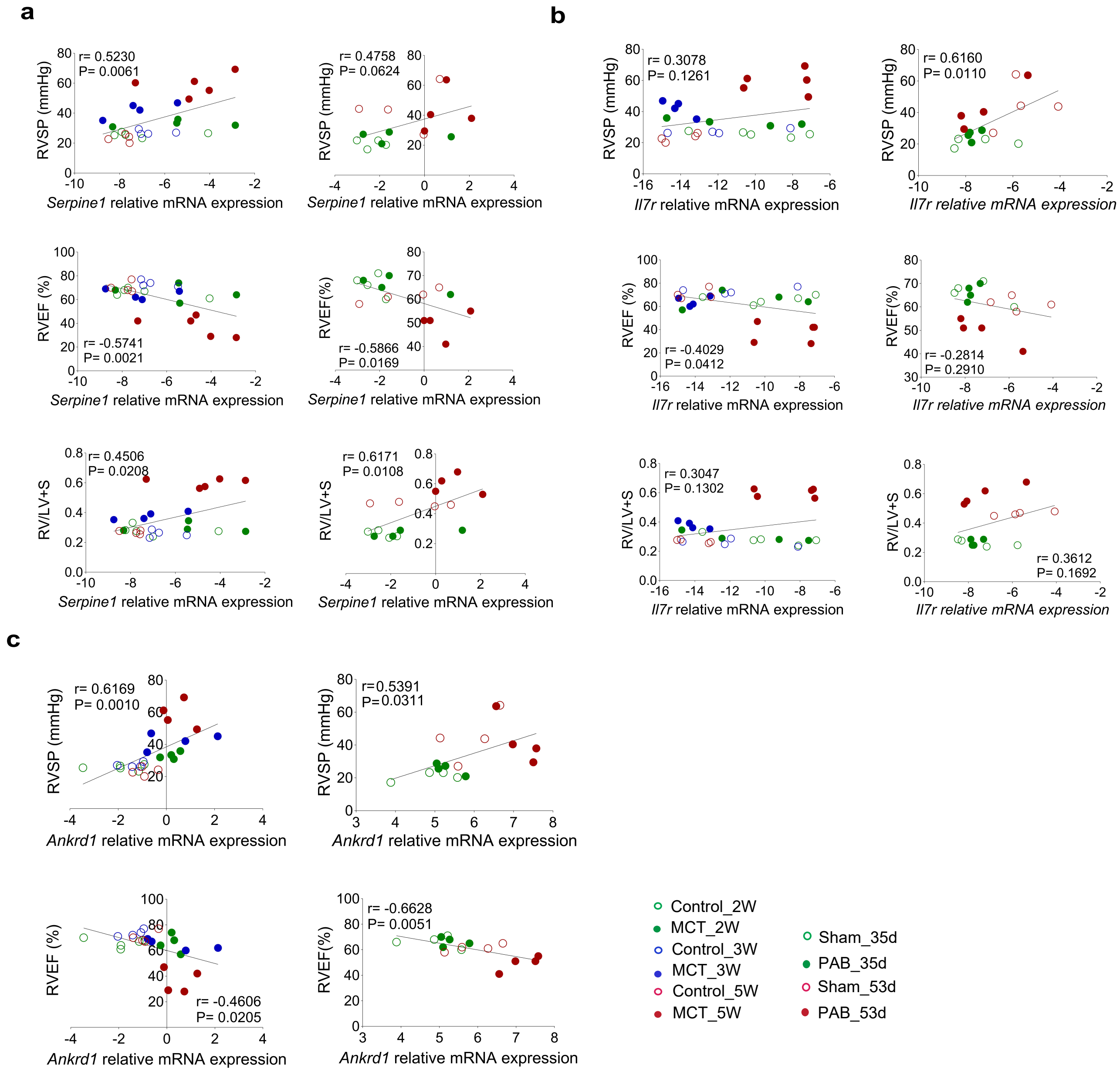

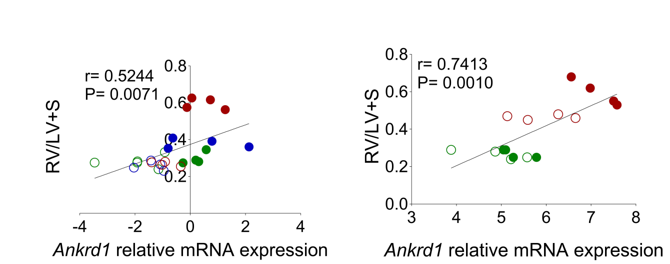

Supplementary Figure 16. Correlations between *Serpine1*, *Il7R,* and *Ankrd1* mRNA levels and the RV hemodynamics parameters in rat models of MCT-induced PH and PAB. Correlation between **a**, *Serpine1* **b**, *Il7R*, and **c**, *Ankrd1* mRNA levels and the RV hemodynamics parameters including RVEF, RV/LV + S, and RVSP are shown for rat models of MCT-induced PH and PAB.

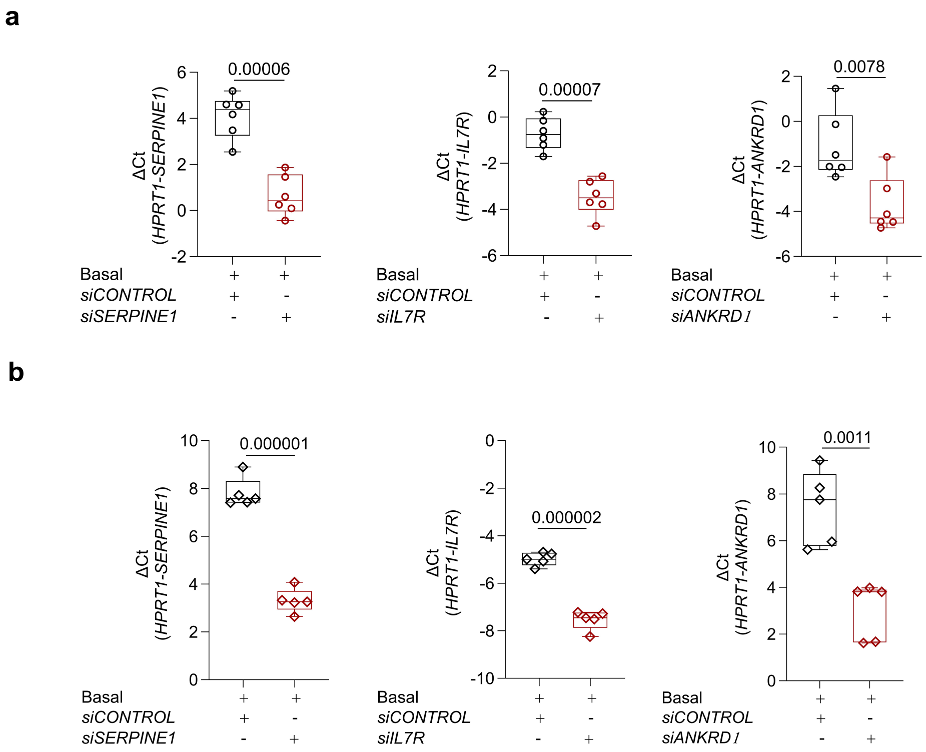

Supplementary Figure 17. siRNA transfection attenuates the expression of *SERPINE1*, *IL7R*, and *ANKRD1* in HCFs and HCMECs. **a**, HCFs and **b**, HCMECs were transfected with *siCONTROL*, *siSERPINE1*, *siIL7R*, and *siANKRD1* for 48 hours. mRNA expression was determined by qPCR and data are presented as mean ± SEM (*n* = 3 biological replicates, each with two technical replicates for HCFs; *n* = 3 biological replicates with only two having two technical replicates for HCMECs; two-tailed unpaired t-test). *HPRT1* was used as an endogenous control. Boxes show the interquartile range (IQR, 25th to 75th percentile), and the central bands indicate the median. The whiskers extend to 1.5 times the IQR above and below the box.

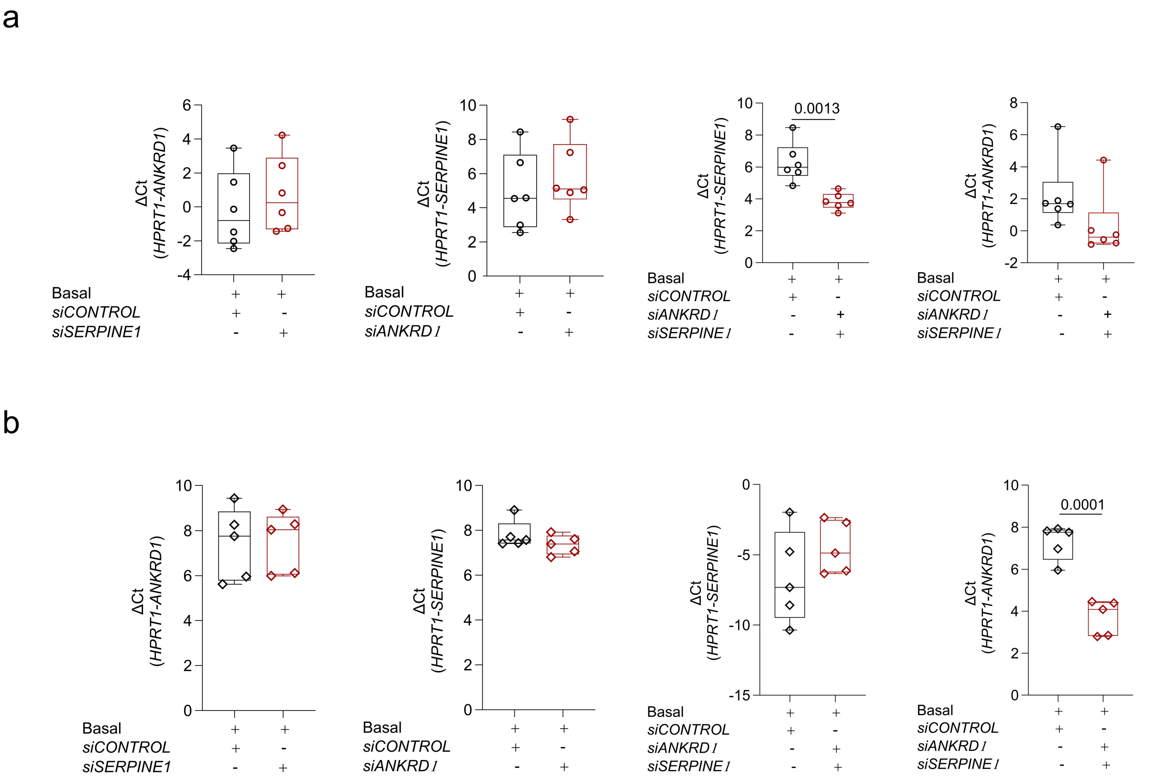

Supplementary Figure 18. Effects of siRNA-mediated knockdown of *SERPINE1* and *ANKRD1* in HCFs and HCMECs. a, HCFs and **b**, HCMECs were transfected with siRNA SMART pools against *SERPINE1*, *ANKRD1*, *ANKRD1/SERPINE1*, and scrambled siRNA for 48 hours. The mRNA expression was determined by qPCR and Data is presented as mean ± SEM (*n* = 3 biological replicates, each with two technical replicates for HCFs; *n* = 3 biological replicates with only two having two technical replicates for HCMECs; two-tailed unpaired t-test). *HPRT1* was used as endogenous control. Boxes show the interquartile range (IQR, 25th to 75th percentile), and the central bands indicate the median. The whiskers extend to 1.5 times the IQR above and below the box.

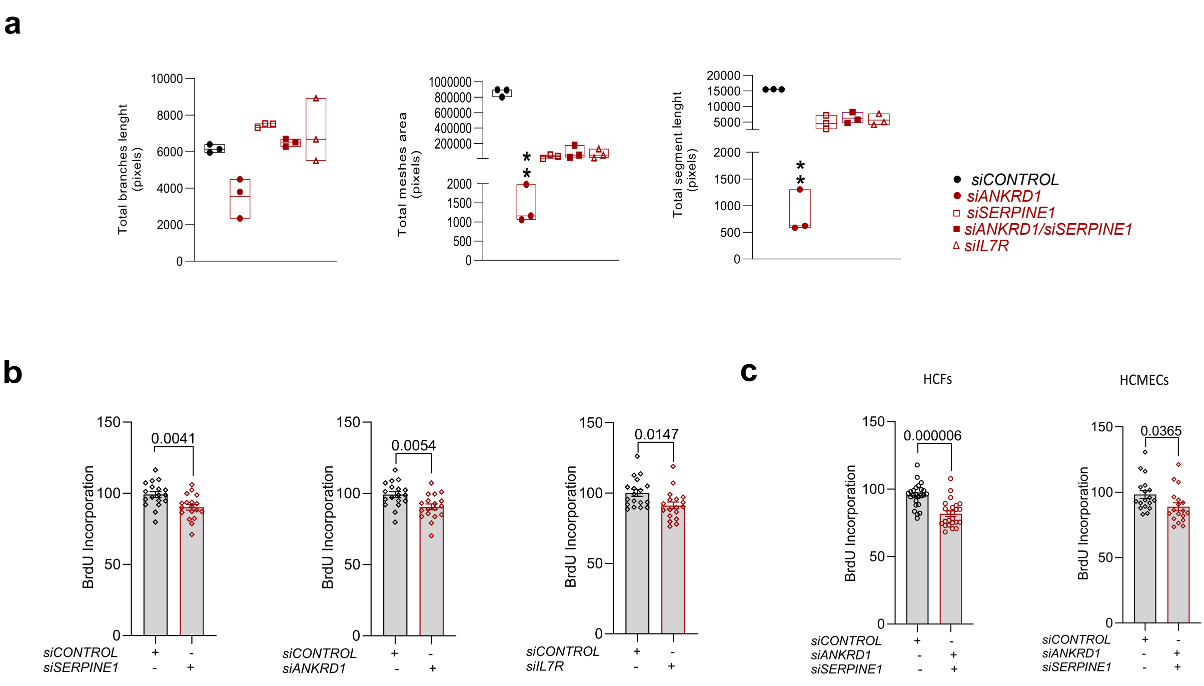

Supplementary Figure 19. Effects of siRNA-mediated knockdown of *SERPINE1*, *ANKRD1*, and *IL7R* on tube formation and proliferation in HCFs and HCMECs. a, HCMECs were transfected with siRNA against *SERPINE1*, *ANKRD1*, *ANKRD1/SERPINE1*, *IL7R*, and scrambled siRNA for 48 h, followed by tube formation assay for 24 h. Total branches length, total meshes area, and total segment length were analyzed and quantified using the Image J software. Data are presented as mean ± SEM (*n* = 3 biological replicates; Kruskal-Wallis test, ***P* = 0.0021 compared to scrambled siRNA. Boxes show the interquartile range (IQR, 25th to 75th percentile), and the central bands indicate the median. The whiskers extend to 1.5 times the IQR above and below the box. **b**, HCMECs were transfected with siRNA for 48 h, and proliferation was measured using BrdU incorporation assay. Data are presented as mean ± SEM (*n* = 3 biological replicates and six technical replicates; two-tailed unpaired t-test). **c**, Cell proliferation was measured in HCFs and HCMECs after 48 hours of double knockdown of ANKRD1 and SERPINE1 followed by BrdU incorporation assay. Data is presented as mean ± SEM (*n* = 4 biological replicates and six technical replicates for HCFs; *n* = 3 biological replicates and six technical replicates for HCMECs; two-tailed unpaired t-test).
