# Supplementary material for: Transcriptional changes of the extracellular matrix in chronic thromboembolic pulmonary hypertension govern right ventricle remodeling and recovery
Source: Nat Cardiovasc Res. 2025 Jul 4;4(7):857–75. doi: 10.1038/s44161-025-00672-8 (PMC12259468; doi:10.1038/s44161-025-00672-8)
Supplement: Supplementary file 2 — Reporting Summary [file 44161_2025_672_MOESM2_ESM.pdf]

Corresponding author(s): Christoph Liebetrau, Soni Savai Pullamsetti

Last updated by author(s): Mar 27, 2025

Reporting Summary

Nature Portfolio wishes to improve the reproducibility of the work that we publish. This form provides structure for consistency and transparency in reporting. For further information on Nature Portfolio policies, see our Editorial Policies and the Editorial Policy Checklist.

Statistics

For all statistical analyses, confirm that the following items are present in the figure legend, table legend, main text, or Methods section.

n/a	Confirmed	
☐	☒	The exact sample size (*n*) for each experimental group/condition, given as a discrete number and unit of measurement
☐	☒	A statement on whether measurements were taken from distinct samples or whether the same sample was measured repeatedly
☐	☒	The statistical test(s) used AND whether they are one- or two-sided<br>*Only common tests should be described solely by name; describe more complex techniques in the Methods section.*
☒	☐	A description of all covariates tested
☐	☒	A description of any assumptions or corrections, such as tests of normality and adjustment for multiple comparisons
☐	☒	A full description of the statistical parameters including central tendency (e.g. means) or other basic estimates (e.g. regression coefficient) AND variation (e.g. standard deviation) or associated estimates of uncertainty (e.g. confidence intervals)
☐	☒	For null hypothesis testing, the test statistic (e.g. *F*, *t*, *r*) with confidence intervals, effect sizes, degrees of freedom and *P* value noted<br>*Give P values as exact values whenever suitable.*
☒	☐	For Bayesian analysis, information on the choice of priors and Markov chain Monte Carlo settings
☐	☒	For hierarchical and complex designs, identification of the appropriate level for tests and full reporting of outcomes
☐	☒	Estimates of effect sizes (e.g. Cohen's *d*, Pearson's *r*), indicating how they were calculated

Our web collection on statistics for biologists contains articles on many of the points above.

Software and code

Policy information about availability of computer code

Data collection

The RV and septum tissue collection for patients with CTEPH and Controls, as well as for the rat animal models are described in detail in the method part of the manuscript. The acquisition of the histopathological, immunohistological, immunofluorescence, and light microscopy images are described in the method part of the manuscript.

Data analysis

The analysis of RNA-seq and the statistical analysis of each experiment are described in detail in the method part of the manuscript. The batch correction was applied to all the RNA-seq data analyses except for Fig. 4, Extended Data Fig 4, and 5 due to the small sample size.
The Human Cardiac Cell Atlas, version 2 (https://www.heartcellatlas.org/index.html) was used to identify the cell types expressing the SERPINE1, IL7R, and ANKRD1. The expression of SERPINE1, IL7R, and ANKRD1 in 704,296 individual cardiac cell types in 12 different cardiac cell types is shown. In addition, the human heart atlas data, generated using Visium technology, were used to map the expression of SERPINE1, IL7R, and ANKRD1 in three normal human right ventricle tissues (> 55 years old) in 5039 cells and in four normal human septal tissues (> 45 years old) in 8643 cells.
The human protein atlas data (courtesy of Human Protein Atlas, https://www.proteinatlas.org/) were used and the correlation matrix of SERPINE1, ANKRD1, and IL7R was analyzed with three virtual reference transcripts for each cell type in myocardial tissue. In addition, the expressions of SERPINE1, ANKRD1, and IL7R and the cell type markers were mapped in different single cell type clusters of cardiac tissue and the interaction networks were examined based on the protein-protein interactions of the IntAct database for SERPINE1, IL7R and ANKRD1.
To explore the transcriptional signature of LV and RV failure, we utilized two publicly available datasets (PMID: 30419824 and PMID: 33787284). The normalized read counts (PMID: 30419824) were obtained from GSE116250 and re-analyzed using the R package limma (PMID: 25605792) with default settings.
For the rat animal models: mCT heart images were segmented by using Analyze 12 software (Analyze Direct, Mayo Clinic); Hemodynamic measurements: data were collected and analyzed using the PowerLab data acquisition system (MPVS-Ultra Single Segment Foundation System, AD Instruments) and LabChart 7 for Windows software.

For the PAB banding and de-banding mouse model, we re-analyzed the publicly available data set (PMID: 31738411) using Transcriptome Analysis Console (TAC, Thermo Fisher Scientific) with DEG thresholds P< 0.05 and fold change < -2 or >2.
The Venn diagrams were generated using Bioinformatics & Evolutionary Genomics (https://bioinformatics.psb.ugent.be/webtools/Venn/), and the Pathway enrichment analysis was carried out using Metascape (www.metascape.org).
The graphical elements are created with Biorender.com. and Extended Fig 10 was designed by Adobe Illustrator (v29.03, Adobe Systems Inc., San Jose, CA, USA).
To quantify the fibrosis in the RV and septum of CTEPH patients, the CellSens Standard software version 4.3 (Evident Scientific, Japan) was used. For the analysis of fibrotic area fractions, a variable number of sections per patient was analyzed to give a tissue area and the corresponding areas of the region of interest (ROI). Areas were summed up per patient, and the area fraction of the ROI per patient was calculated by dividing the summed areas (ROI area/tissue area). Differences in the area fractions between the different risk groups were analyzed in R 4.1.1 (R Core Team (2024). R: A Language and Environment for Statistical Computing. R Foundation for Statistical Computing, Vienna, Austria. https://www.R-project.org/) using a weighted generalized linear model of the quasi-binomial family, using the total tissue areas per patient as weights. The area fractions in RV and septum were compared using a paired-sample t-test on the log fractions, using the total tissue area per patient as weights.
The cross-sectional area of cardiomyocytes and tissue vascularization was assessed using Olympus CellSens Entry 2.3 software.
The clinical information of the patients with CTEPH at prePEA and postPEA in both the exploratory (at A-prePEA) and confirmatory (at B-prePEA and B-postPEA) cohorts is extensively provided in the Supplementary Tables, Extended Data Figures, and within the manuscript.
The remaining data in this study are provided in the methods, supplementary information, and Source data section of the manuscript.

For manuscripts utilizing custom algorithms or software that are central to the research but not yet described in published literature, software must be made available to editors and reviewers. We strongly encourage code deposition in a community repository (e.g. GitHub). See the Nature Portfolio guidelines for submitting code & software for further information.

Data

Policy information about availability of data

All manuscripts must include a data availability statement. This statement should provide the following information, where applicable:
- Accession codes, unique identifiers, or web links for publicly available datasets
- A description of any restrictions on data availability
- For clinical datasets or third party data, please ensure that the statement adheres to our policy

Gene expression profiling data have been deposited in the NCBI's gene expression omnibus repository (GEO) under accession number GSE249697. The dataset can be accessed at: https://www.ncbi.nlm.nih.gov/geo/query/acc.cgi?acc=GSE249697.
The code repository for the RNA-seq analysis is accessible at: https://github.com/loosolab/Code_Jafari_et_al_2024_cteph_rv_reverse_remodeling

Research involving human participants, their data, or biological material

Policy information about studies with human participants or human data. See also policy information about sex, gender (identity/presentation), and sexual orientation and race, ethnicity and racism.

Reporting on sex and gender

Both males and females have entered the study. The sex of the participants was not considered in this study, and it was defined by self-report as well as by the person who filled the study questionnaire. The sex of each patient with CTEPH in both the exploratory and confirmatory cohorts, as well as in control group, is provided in the Supplementray Table.

Reporting on race, ethnicity, or other socially relevant groupings

The reporting on race, ethnicity, or other socially relevant groupings was not considered in this study. However, all the CTEPH patients included in the study were Caucasian.

Population characteristics

The demographic characteristics and hemodynamic parameters of the patients with CTEPH in both the exploratory and confirmatory cohort are extensivly detailed in the Supplementary Tables, Extended Data, and within the manuscript.
The age range of the 96 CTEPH patinets was 20.1-80.02 years. and the age range of the control group was 23.17 to 54.39 years. The CTEPH cohort consisted of 26 females and 69 males, with one patinet's sex not reported. The control group consist of 7 males and 3 females.

Recruitment

In this study, the participants who were treated by pulmonary endarterectomy (PEA) at Kerckhoff Heart and Thorax Centre between 2016 and 2020 were included. Clinical examination, echocardiography, 12-lead electrocardiogram, laboratory tests, 6-minute walk test, ventilation-perfusion-scan, computed tomography angiography, right-left-heart catheterization, and pulmonary angiography were assessed for all patients before PEA. The final diagnosis of CTEPH was made according to the guidelines in symptomatic patients after three months of effective anticoagulation, with a mean pulmonary arterial pressure (mPAP) ≥25 mmHg at rest and typical obstructive pulmonary vascular lesions on imaging diagnostics. All patients were presented in an interdisciplinary CTEPH conference to define the therapeutic concept and approve the feasibility of surgical PEA as the first-line therapy. The RV biopsies (A-prePEA, n=14 and B-prePEA, n=88) were obtained from patients who underwent the PEA. The septum biopsies (B-prePEA_septum; n = 3) were taken one day before PEA. Additionally, the septum biopsy (B-postPEA; n = 28) was taken 12 months after PEA.
Tissue samples from healthy controls were collected from the same patient (n = 10) for both RV and septum. The tissue samples for control were collected in the Department of Heart Failure and Transplantology, National Institute of Cardiology, Warsaw, Poland. Healthy human hearts were obtained from organ donor patients (Control, n = 10) whose hearts were not used for transplantation due to technical reasons (e.g., donor/recipient incompatibility). The donors did not have any relevant previous cardiological history or any abnormalities in ECG and echocardiography (LV dimensions/contractility within normal ranges). The tissue samples from the ventricular free wall were taken (avoiding scarred, fibrotic, or adipose tissue,

endocardium, epicardium, or coronary vessels).

Ethics oversight

All the patients including those with CTEPH in both exploratory and confirmatory cohorts, were given written informed consent. This study was approved by the ethics board of the Justus Liebig University of Giessen (AZ 44/ 14, 144/ 11, 145/ 11, 146/ 11, 199/ 15) and is in accordance with the declaration of Helsinki. For the 10 control subjects, all experimental procedures were conducted in accordance with the ethical standards of the responsible institutional and national committee on human experimentation, as outlined in the Helsinki Declaration (1975). Written informed consent was obtained from all patients involved in the study according to the protocol approved by the Local Ethics Committees of the National Institute of Cardiology, Warsaw, Poland (approval number: IK-NPIA-0021-14/1426/18).

Note that full information on the approval of the study protocol must also be provided in the manuscript.

Field-specific reporting

Please select the one below that is the best fit for your research. If you are not sure, read the appropriate sections before making your selection.

☒ Life sciences ☐ Behavioural & social sciences ☐ Ecological, evolutionary & environmental sciences

For a reference copy of the document with all sections, see nature.com/documents/nr-reporting-summary-flat.pdf

Life sciences study design

All studies must disclose on these points even when the disclosure is negative.

Sample size

The sample sizes of the human biopsies from RV and septum, as well as the sample size for the rat RV, were determined based on the availability of the tissue samples.
In rat animal models, the sample size was pre-established based on the right ventricular systolic pressure (RVSP) and right ventricular hypertrophy (RV/LV+S).
Sample sizes for the microscopy analyses and for all the in vivo and in vitro experimental models are indicated in each figure.

Data exclusions

RNA-seq data is available for 24 septum biopsies (B-postPEA_septum), of which 22 are paired with corresponding RV biopsies. However, in our manuscript, we presented data for 21 patients [B-postPEA _septum(n=21) and B-prePEA_RV (n=21)]. Patient Nr. 96 was excluded due to its identification as an outlier in the PCA plot. The decision to exclude this patient was made to minimize data variation (Supplementray Table). For animal studies (PAB/Sham and MCT/Control rats) no exclusion criteria were pre-established. One sample from the Control_3W has been removed due to an extremely low RNA concentration.

Replication

All the biological and technical replicates related to in vitro experimental models are indicated in the respective figure legend. The rat animal studies were performed in independent biological experiments.
A total of 96 CTEPH patients included in this study, divided into an exploratory cohort (n = 14) and a confirmatory cohort (n = 89). Seven patients with RV biopsies were present in both cohorts. Additionally, One patient from the exploratory cohort, who had only a septum biopsy, was also included in the confirmatory cohort. For the RNA-seq analysis, 115 cardiac tissues from CTEPH patients were analysed, along with 10 control RV biopsies and 10 septum biopsies from the same patients.
In the exploratory cohort (A-prePEA_RV, n = 14) the RV tissue from 14 patients was sequenced. In the confirmatory cohort, out of 88 RV biopsy samples, RNA -seq was performed on 71 samples and histological analysis on 43 samples, with 26 samples overlapping between the two datasets. The allocation to RNA-seq or histology was not based on any pre-selection, bias, or stratification criteria, but rather determined solely by the amount of tissue available from each biopsy. RNA-seq data were available for 24 septum biopsies (B-postPEA_septum), of which 22 are paired with corresponding RV biopsies. However, in the manuscript, we presented data for 21 patients [B-postPEA_septum (n = 21) and B-prePEA_RV (n = 21)]. The following patients were excluded:
-Patient Nr. 96 (Pat 96-FU) was excluded due to its identification as an outlier in the PCA plot.
-Patient Nr. 83 (Pat 83-FU) was excluded due to the lack of a corresponding RV biopsy.
-Patient Nr.9 (Pat 9-FU) was excluded because there was no corresponding RV in the confirmatory cohort. This patient's RV was investigated in the exploratory cohort.
For three patients (IDs: 33, 72, and 91) the septum biopsy before PEA (B-prePEA_septum, n = 3; Pat-33-PreBL, Pat-72-PreBL, Pat-91-PreBL) and their corresponding septum biopsy after PEA (B-postPEA_septum, n = 3; Pat-33-FU, Pat-72-FU, Pat-91-FU) were sequenced. Additionally, the RV biopsies of these patients before PEA (B-prePEA_RV, n = 3; Pat 33-BL, Pat 72-BL, Pat 91-BL and their septum biopsy (B-postPEA_septum, n = 3, Pat 33-FU, Pat 72-FU, Pat 91-FU) were sequenced.
For histological analysis, we included 47 RV biopsies (B-prePEA_RV, n = 43) with 26 RV tissues common between sequenced RV samples. 13 septum biopsies (n = 13), with 10 patients having sequenced tissue. For the vascularization, we included 7 septum biopsies from the same patients. 7 RV and 7 septum biopsies from the same patient and all these patients were common with the previously sequenced RV and septum biopsies. All the biological replicates related to the human-derived data are reported in the respective figure legend and the methods part of the manuscript.

Randomization

No randomization was applied to the participants of this study. The CTEPH patients were grouped based on their clinical parameters.
In rat animal models the animals were assigned to the study group based on the level of right ventricular hypertrophy (RV/LV+S) and right ventricular ejection fraction (RVEF).

Blinding

All the human biospecimens were processed in a standardized manner by experienced staff, who were blinded to the clinical data. The RNA-seq data were performed blinded on the RV and septum of the subjects. Histopathological, immunohistological, immunofluorescence and light microscopy procedures were also blinded during data collection and analysis.
In rat animal models, the data were acquired and analyzed in a blind fashion. Researchers were blinded to the studied groups either during

mCT acquisition and analyses or invasive hemodynamics measurements.

Reporting for specific materials, systems and methods

We require information from authors about some types of materials, experimental systems and methods used in many studies. Here, indicate whether each material, system or method listed is relevant to your study. If you are not sure if a list item applies to your research, read the appropriate section before selecting a response.

Materials & experimental systems

n/a	Involved in the study
☐	☒ Antibodies
☐	☒ Eukaryotic cell lines
☐	☐ Palaeontology and archaeology
☐	☒ Animals and other organisms
☐	☒ Clinical data
☐	☐ Dual use research of concern
☐	☐ Plants

Methods

n/a	Involved in the study
☐	☐ ChIP-seq
☐	☐ Flow cytometry
☐	☐ MRI-based neuroimaging

Antibodies

Antibodies used

CD34 monoclonal antibody Novocastra™ (1:250, # NCL-L-END, Clone QBEnd/10, Leica Biosystems, Newcastle Ltd) and the BOND Polymer Refine Detection System (# DS9800, Leica Biosystems, Newcastle Ltd)
PAI1 monoclonal antibody (SERPINE1, 1:200, #MA1-40224, Clone MA-33H1F7, ThermoFisher)
COL1A1 polyclonal antibody (1:500, #PA5-29569, ThermoFisher)
CD127 polyclonal antibody (IL7R, 1:200, #PA5-102399, ThermoFisher)
Alpha-smooth muscle actin antibody (αSMA, 1:2000, #NB300-978, Novus)
CARP polyclonal antibody (ANKRD1, 1:400, # PA5-101170, ThermoFisher)
cardiac troponin T monoclonal antibody (13-11) (cTnT, 1:1000, #MA5 12960, Clone 13-11, ThermoFisher)
secondary antibodies:
anti-mouse 594, 1:500; #A21203, ThermoFisher
anti-rabbit 488, 1:500, #A11008, ThermoFisher
anti-rabbit 594, 1:500, #A32740, ThermoFisher
anti-goat 488, 1:500, #A21467, ThermoFisher
anti-mouse 488, 1:1000, #A11029, ThermoFisher

Validation

Anti-CD34 antibody (DOI: 10.1111/j.1365-2559.1990.tb00713.x)
All the antibodies were validated by the corresponding manufacturers:
Anti-PAI1 antibody (https://www.thermofisher.com/antibody/product/PAI1-Antibody-clone-MA-33H1F7-Monoclonal/MA1-40224)
Anti-COL1A1 antibody (https://www.thermofisher.com/antibody/product/COL1A1-Antibody-Polyclonal/PA5-29569)
Anti-CD127 antibody (https://www.thermofisher.com/antibody/product/CD127-Antibody-Polyclonal/PA5-102399)
Anti-Alpha-smooth muscle actin antibody (https://www.novusbio.com/products/alpha-smooth-muscle-actin-antibody_nb300-978?srsltid=AfmBOoqZPIJVkLIAPGfiOmKAIVxk16OUTceBBwUqsRHvTKK8tfq4qucL)
Anti-CARP antibody (https://www.thermofisher.com/antibody/product/CARP-Antibody-Polyclonal/PA5-101170)
Anti-cTnT (https://www.thermofisher.com/antibody/product/Cardiac-Troponin-T-Antibody-clone-13-11-Monoclonal/MA5-12960)
Anti-mouse 594 (https://www.thermofisher.com/antibody/product/Donkey-anti-Mouse-IgG-H-L-Highly-Cross-Adsorbed-Secondary-Antibody-Polyclonal/A-21203)
Anti-rabbit 488 (https://www.thermofisher.com/antibody/product/Goat-anti-Rabbit-IgG-H-L-Cross-Adsorbed-Secondary-Antibody-Polyclonal/A-11008)
Anti-rabbit 594 (https://www.thermofisher.com/antibody/product/Goat-anti-Rabbit-IgG-H-L-Highly-Cross-Adsorbed-Secondary-Antibody-Polyclonal/A32740)
Anti-goat 488 (https://www.thermofisher.com/antibody/product/Chicken-anti-Goat-IgG-H-L-Cross-Adsorbed-Secondary-Antibody-Polyclonal/A-21467)
Anti-mouse 488 (https://www.thermofisher.com/antibody/product/Goat-anti-Mouse-IgG-H-L-Highly-Cross-Adsorbed-Secondary-Antibody-Polyclonal/A-11029)

Eukaryotic cell lines

Policy information about cell lines and Sex and Gender in Research

Cell line source(s)

Primary human cardiac fibroblasts were purchased from ScienCell Research Laboratories and primary human cardiac microvascular endothelial cells were purchased from PromoCell GmbH.

Authentication

None of the cells were authenticated.

Mycoplasma contamination	Cells were regularly tested for mycoplasma by PCR and were tested negative.
Commonly misidentified lines (See ICLAC register)	No commonly misidentified cell lines were used in this study.

Palaeontology and Archaeology

Specimen provenance	*Provide provenance information for specimens and describe permits that were obtained for the work (including the name of the issuing authority, the date of issue, and any identifying information). Permits should encompass collection and, where applicable, export.*
Specimen deposition	*Indicate where the specimens have been deposited to permit free access by other researchers.*
Dating methods	*If new dates are provided, describe how they were obtained (e.g. collection, storage, sample pretreatment and measurement), where they were obtained (i.e. lab name), the calibration program and the protocol for quality assurance OR state that no new dates are provided.*

☐ Tick this box to confirm that the raw and calibrated dates are available in the paper or in Supplementary Information.

Ethics oversight	*Identify the organization(s) that approved or provided guidance on the study protocol, OR state that no ethical approval or guidance was required and explain why not.*

Note that full information on the approval of the study protocol must also be provided in the manuscript.

Animals and other research organisms

Policy information about studies involving animals; ARRIVE guidelines recommended for reporting animal research, and Sex and Gender in Research

Laboratory animals	The RV tissue was obtained from pulmonary artery banding (PAB)/Sham operated and monocrotaline (MCT)-injected/Control rats. The strain of the animals was Sprague-Dawley rats. In PAB/Sham rat models the hemodynamics measurements and RV tissues were obtained from animals on days 35 and 53 after PAB/sham operation. In MCT/Control rats the hemodynamics measurements and RV tissues collection were performed after 2, 3, and 5 weeks of MCT injection.
Wild animals	This study did not involve wild animals.
Reporting on sex	The finding of this study was applied to only male rat animal models.
Field-collected samples	This study did not involve field-collected samples.
Ethics oversight	The animal study protocols were conducted in accordance with the National Institute of Health Guidelines on the Use of Laboratory Animals. The study protocols were approved by the University Animal Care Committee and the Federal Authorities for Animal Research of the Regierungspräsidium Giessen (GI 20/10 Nr G92/2017 RP Giessen), Hessen, Germany.

Note that full information on the approval of the study protocol must also be provided in the manuscript.

Clinical data

Policy information about clinical studies

All manuscripts should comply with the ICMJE guidelines for publication of clinical research and a completed CONSORT checklist must be included with all submissions.

Clinical trial registration	Not applicable, as the study was observational.
Study protocol	The study population and clinical work-up of this study are explained in the methods section of the manuscript. For the patients with CTEPH the study was approved by the ethics board of the Justus Liebig University of Giessen (AZ 44 / 14, 144 / 11, 145 / 11, 146 / 11, 199 / 15) and in accordance with the declaration of Helsinki. For the control participants, the study protocol was approved by the Local Ethics Committees of the National Institute of Cardiology, Warsaw, Poland (approval number: IK-NPIA-0021-14/1426/18).
Data collection	The human RV and septum acquisition from the RV and septum of patients with CTEPH and control subjects were explained in detail in the Methods section of the revised manuscript.
Outcomes	In this prospective study, patients with CTEPH, are divided into two distinct cohorts: an exploratory cohort and a confirmatory cohort. The patients in each cohort were risk stratified based on their clinical parameters and the European Society of Cardiology (ESC) and the European Respiratory Society (ERS) guidelines using a three-strata model into a moderate, intermediate, and severe risk group. In addition, this study included a subgroup of patients, with postPEA septum biopsies collected 12 months after PEA, and all were matched with prePEA counterparts. Additionally, in the confirmatory cohort, in the subgroup of 3 patients, with the biopsies before PEA from RV (B-prePEA_RV, n = 3) and 12 months after PEA from the septum (B-postPEA_septum, n = 3), the biopsy was obtained from the septum before PEA (B-prePEA_septum, n = 3). The clinical parameters of all the patients with CTEPH are shown in the Supplementary Tables.

Dual use research of concern

Policy information about dual use research of concern

Hazards

Could the accidental, deliberate or reckless misuse of agents or technologies generated in the work, or the application of information presented in the manuscript, pose a threat to:

No	Yes	
☐	☐	Public health
☐	☐	National security
☐	☐	Crops and/or livestock
☐	☐	Ecosystems
☐	☐	Any other significant area

Experiments of concern

Does the work involve any of these experiments of concern:

No	Yes	
☐	☐	Demonstrate how to render a vaccine ineffective
☐	☐	Confer resistance to therapeutically useful antibiotics or antiviral agents
☐	☐	Enhance the virulence of a pathogen or render a nonpathogen virulent
☐	☐	Increase transmissibility of a pathogen
☐	☐	Alter the host range of a pathogen
☐	☐	Enable evasion of diagnostic/detection modalities
☐	☐	Enable the weaponization of a biological agent or toxin
☐	☐	Any other potentially harmful combination of experiments and agents

Plants

Seed stocks	*Report on the source of all seed stocks or other plant material used. If applicable, state the seed stock centre and catalogue number. If plant specimens were collected from the field, describe the collection location, date and sampling procedures.*
Novel plant genotypes	*Describe the methods by which all novel plant genotypes were produced. This includes those generated by transgenic approaches, gene editing, chemical/radiation-based mutagenesis and hybridization. For transgenic lines, describe the transformation method, the number of independent lines analyzed and the generation upon which experiments were performed. For gene-edited lines, describe the editor used, the endogenous sequence targeted for editing, the targeting guide RNA sequence (if applicable) and how the editor was applied.*
Authentication	*Describe any authentication procedures for each seed stock used or novel genotype generated. Describe any experiments used to assess the effect of a mutation and, where applicable, how potential secondary effects (e.g. second site T-DNA insertions, mosiacism, off-target gene editing) were examined.*

ChIP-seq

Data deposition

☐ Confirm that both raw and final processed data have been deposited in a public database such as GEO.

☐ Confirm that you have deposited or provided access to graph files (e.g. BED files) for the called peaks.

Data access links<br>*May remain private before publication.*	*For "Initial submission" or "Revised version" documents, provide reviewer access links. For your "Final submission" document, provide a link to the deposited data.*
Files in database submission	*Provide a list of all files available in the database submission.*
Genome browser session<br>(e.g. UCSC)	*Provide a link to an anonymized genome browser session for "Initial submission" and "Revised version" documents only, to enable peer review. Write "no longer applicable" for "Final submission" documents.*

Methodology

Replicates | *Describe the experimental replicates, specifying number, type and replicate agreement.*

Sequencing depth | *Describe the sequencing depth for each experiment, providing the total number of reads, uniquely mapped reads, length of reads and whether they were paired- or single-end.*

Antibodies | *Describe the antibodies used for the ChIP-seq experiments; as applicable, provide supplier name, catalog number, clone name, and lot number.*

Peak calling parameters | *Specify the command line program and parameters used for read mapping and peak calling, including the ChIP, control and index files used.*

Data quality | *Describe the methods used to ensure data quality in full detail, including how many peaks are at FDR 5% and above 5-fold enrichment.*

Software | *Describe the software used to collect and analyze the ChIP-seq data. For custom code that has been deposited into a community repository, provide accession details.*

Flow Cytometry

Plots

Confirm that:

☐ The axis labels state the marker and fluorochrome used (e.g. CD4-FITC).

☐ The axis scales are clearly visible. Include numbers along axes only for bottom left plot of group (a 'group' is an analysis of identical markers).

☐ All plots are contour plots with outliers or pseudocolor plots.

☐ A numerical value for number of cells or percentage (with statistics) is provided.

Methodology

Sample preparation | *Describe the sample preparation, detailing the biological source of the cells and any tissue processing steps used.*

Instrument | *Identify the instrument used for data collection, specifying make and model number.*

Software | *Describe the software used to collect and analyze the flow cytometry data. For custom code that has been deposited into a community repository, provide accession details.*

Cell population abundance | *Describe the abundance of the relevant cell populations within post-sort fractions, providing details on the purity of the samples and how it was determined.*

Gating strategy | *Describe the gating strategy used for all relevant experiments, specifying the preliminary FSC/SSC gates of the starting cell population, indicating where boundaries between "positive" and "negative" staining cell populations are defined.*

☐ Tick this box to confirm that a figure exemplifying the gating strategy is provided in the Supplementary Information.

Magnetic resonance imaging

Experimental design

Design type | *Indicate task or resting state; event-related or block design.*

Design specifications | *Specify the number of blocks, trials or experimental units per session and/or subject, and specify the length of each trial or block (if trials are blocked) and interval between trials.*

Behavioral performance measures | *State number and/or type of variables recorded (e.g. correct button press, response time) and what statistics were used to establish that the subjects were performing the task as expected (e.g. mean, range, and/or standard deviation across subjects).*

Acquisition

Imaging type(s) — *Specify: functional, structural, diffusion, perfusion.*

Field strength — *Specify in Tesla*

Sequence & imaging parameters — *Specify the pulse sequence type (gradient echo, spin echo, etc.), imaging type (EPI, spiral, etc.), field of view, matrix size, slice thickness, orientation and TE/TR/flip angle.*

Area of acquisition — *State whether a whole brain scan was used OR define the area of acquisition, describing how the region was determined.*

Diffusion MRI ☐ Used ☐ Not used

Preprocessing

Preprocessing software — *Provide detail on software version and revision number and on specific parameters (model/functions, brain extraction, segmentation, smoothing kernel size, etc.).*

Normalization — *If data were normalized/standardized, describe the approach(es): specify linear or non-linear and define image types used for transformation OR indicate that data were not normalized and explain rationale for lack of normalization.*

Normalization template — *Describe the template used for normalization/transformation, specifying subject space or group standardized space (e.g. original Talairach, MNI305, ICBM152) OR indicate that the data were not normalized.*

Noise and artifact removal — *Describe your procedure(s) for artifact and structured noise removal, specifying motion parameters, tissue signals and physiological signals (heart rate, respiration).*

Volume censoring — *Define your software and/or method and criteria for volume censoring, and state the extent of such censoring.*

Statistical modeling & inference

Model type and settings — *Specify type (mass univariate, multivariate, RSA, predictive, etc.) and describe essential details of the model at the first and second levels (e.g. fixed, random or mixed effects; drift or auto-correlation).*

Effect(s) tested — *Define precise effect in terms of the task or stimulus conditions instead of psychological concepts and indicate whether ANOVA or factorial designs were used.*

Specify type of analysis: ☐ Whole brain ☐ ROI-based ☐ Both

Statistic type for inference — *Specify voxel-wise or cluster-wise and report all relevant parameters for cluster-wise methods.*

(See Eklund et al. 2016)

Correction — *Describe the type of correction and how it is obtained for multiple comparisons (e.g. FWE, FDR, permutation or Monte Carlo).*

Models & analysis

n/a	Involved in the study
☐	☐ Functional and/or effective connectivity
☐	☐ Graph analysis
☐	☐ Multivariate modeling or predictive analysis

Functional and/or effective connectivity — *Report the measures of dependence used and the model details (e.g. Pearson correlation, partial correlation, mutual information).*

Graph analysis — *Report the dependent variable and connectivity measure, specifying weighted graph or binarized graph, subject- or group-level, and the global and/or node summaries used (e.g. clustering coefficient, efficiency, etc.).*

Multivariate modeling and predictive analysis — *Specify independent variables, features extraction and dimension reduction, model, training and evaluation metrics.*
